# Synthesis of the Dimeric Diarylheptanoids Alpinidinoid C and Officinine B Enabled by Blue-Light-Mediated Triple-Minisci-Type Alkylation

**DOI:** 10.1021/acs.orglett.4c03227

**Published:** 2024-10-10

**Authors:** Daniel C. Schultz, Upendra Rathnayake, Renn A. Duncan, Alexa E. Richardson, Aaron M. Bender

**Affiliations:** Warren Center for Neuroscience Drug Discovery and Department of Pharmacology, Vanderbilt University, Nashville, Tennessee 37232, United States

## Abstract

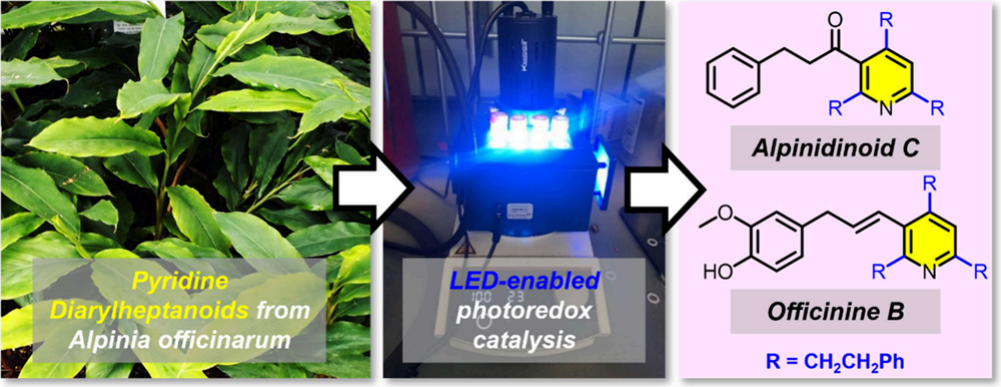

The first syntheses of the *Alpinia officinarum* natural products alpinidinoid C and officinine B are reported. These unusual dimeric diarylheptanoids were accessed from a 3-substituted pyridine intermediate via a blue-light-mediated, triple-Minisci-type alkylation. Very few reports utilize *N*-(acyloxy)phthalimides (NAPs) in the construction of natural products, and the syntheses reported herein highlight the power of this methodology toward the orthogonal construction of highly substituted arenes.

*Alpinia officinarum* Hance is a plant in the ginger family (Zingiberaceae) which is widely cultivated in Southeast Asia.^1^ The rhizomes of *A. officinarum* have both culinary and medicinal applications; these rhizomes (galangal) are prized for both their highly aromatic scent as well as their utility in traditional Chinese medicine for the relief of stomach pain, cold symptoms, and inflammation.^2^ Among other chemical constituents, monomeric and dimeric diarylheptanoids are characteristic isolates of *A. officinarum* rhizomes, and compounds in this class are known to elicit a range of interesting biological responses including antioxidant, antibacterial and anticancer activity.^3^ Recently, Liu and colleagues found that the dextrorotatory enantiomer of the dimeric diarylheptanoid alpinidinoid A (**1**, Figure 1) significantly reduced neuronal apoptosis through activation of the AKT/mTOR signaling pathway.^4^

**Figure 1:**
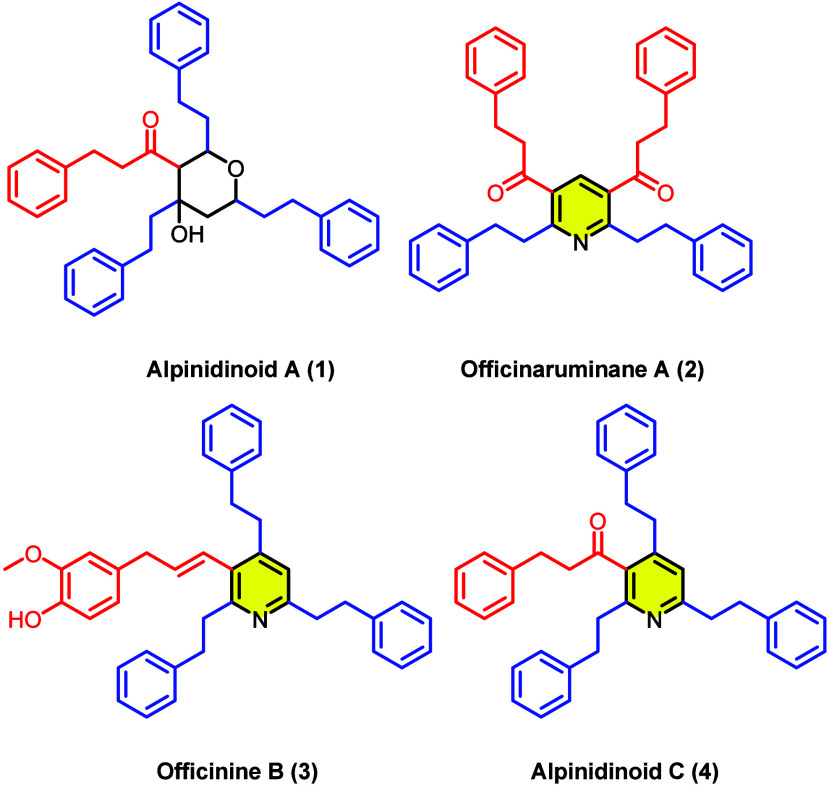
Structures of selected dimeric diarylheptanoids from *Alpinia officinarum*.

Among the many dimeric diarylheptanoids that have been isolated from *A. officinarum*, we were intrigued by a series of reports describing diarylheptanoid monomers connected through a pyridine linkage (**2**–**4**, Figure 1).^4,5^ This type of linkage is rare in the context of the diarylheptanoid dimers, and to our knowledge only 3 representative analogs are currently known: officinaruminane A (**2**), officinine B (**3**), and the recently reported alpinidinoid C (**4**, see Figure 1).^4,5^ Additionally, although the first member of this pyridine-linked family of compounds, officinaruminane A (**2**), was first disclosed in 2010,^5a^ to date there are no reported syntheses of any compound in this class. Intrigued by the structural uniqueness of these compounds among natural products, and the absence of any reported syntheses, we sought to complete the first total synthesis of officinine B (**3**) and alpinidinoid C (**4**). We envisioned that both compounds would be accessible through a divergent route from a common substituted pyridine intermediate.

Compounds **3** and **4** both feature a densely substituted pyridine core, with a conserved 2,4,6-triphenethyl substitution pattern. **3** and **4** diverge, however, with respect to their substitution at the 3-position: officinine B (**3**) features an olefin substitution, whereas alpinidinoid C (**4**) features a phenylpropane pendant which is oxidized as the ketone at the linkage position. We imagined that the dense and asymmetric C-linked substitution pattern decorating both compounds would challenge selective pendant installation via traditional C–C cross-coupling reactions and sought to develop an approach that would initially install the conserved 2,4,6-triphenethyl substitution while leaving a common handle at the 3-position. Such an approach would rely on the innate electronic character of a simple, monosubstituted pyridine starting material, as opposed to a stepwise cross-coupling strategy (which would require the generation of complex, densely halogenated pyridines with no guarantee of selectivity in the cross-coupling reactions).

The Minisci reaction is a powerful method by which to form C–C bonds with electron-deficient heteroaromatic compounds.^6^ Historically, this reaction is driven by radicals derived from carboxylic acids via silver-mediated oxidative decarboxylation,^6b^ but a number of reports have dramatically expanded the scope and versatility of this reaction since the seminal publication. Boronic acids, trifluoroborates, sulfinates, alcohols, alkyl halides and other functional groups can now be leveraged as coupling partners for Minisci-type heteroarene substitutions, and visible-light-mediated metallaphotoredox chemistry has proven particularly useful for these kinds of transformations.^6a,7^ We therefore wondered if a simple, electron-poor pyridine, electronically predisposed to a Minisci-type substitution reaction, could serve as a means to generate a common 2,4,6-triphenethyl-substituted intermediate by which to access **3** and **4** (Figure 2A).

**Figure 2:**
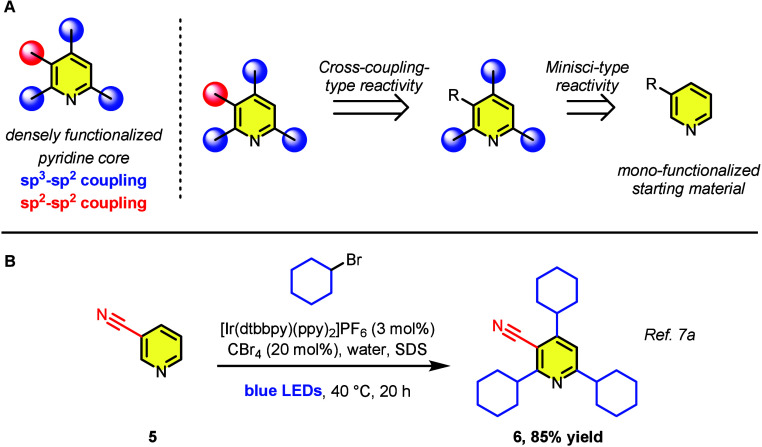
(A) Retrosynthetic strategy to access tetrasubstituted pyridines via a triple-Minisci-type alkylation. (B) Known example of a triple-Minisci-type alkylation on 3-cyanopyridine (**5**).^7a^

Despite the utility of the Minisci reaction, we were aware that our proposed one-pot sp^3^-sp^2^ alkylation strategy faced a number of challenges at the outset. Such a reaction would not only require a starting material that could accommodate this kind of rare triple alkylation, but would also need to be amenable to substitution with a challenging primary radical species.^7a,7b^ Additionally, substrates that are electronically predisposed to Minisci alkylations *a priori* are often rendered unreactive after an initial alkylation step.^7c,7e^ To our knowledge, there currently exists only a single report in the literature which describes a high-yielding triple alkylation of a pyridine under Minisci-type conditions.^7a^ This report, which utilizes photoredox catalysis in SDS-micelles, describes the synthesis of 2,4,6-tricyclohexane-substituted compound **6** via 3-cyanopyridine (**5**; see Figure 2B). Unfortunately, in our hands, similar conditions led to no observable 2,4,6-triphenethylnicotinonitrile (**7**) using (2-bromoethyl)benzene (see Table 1, entry 1). This lack of reactivity could be a result of the established difficulty in affecting this kind of substitution via primary radicals,^7a,7b^ and/or differences in LED wattage/reaction setup between laboratories (see the Supporting Information).

**Table 1:**
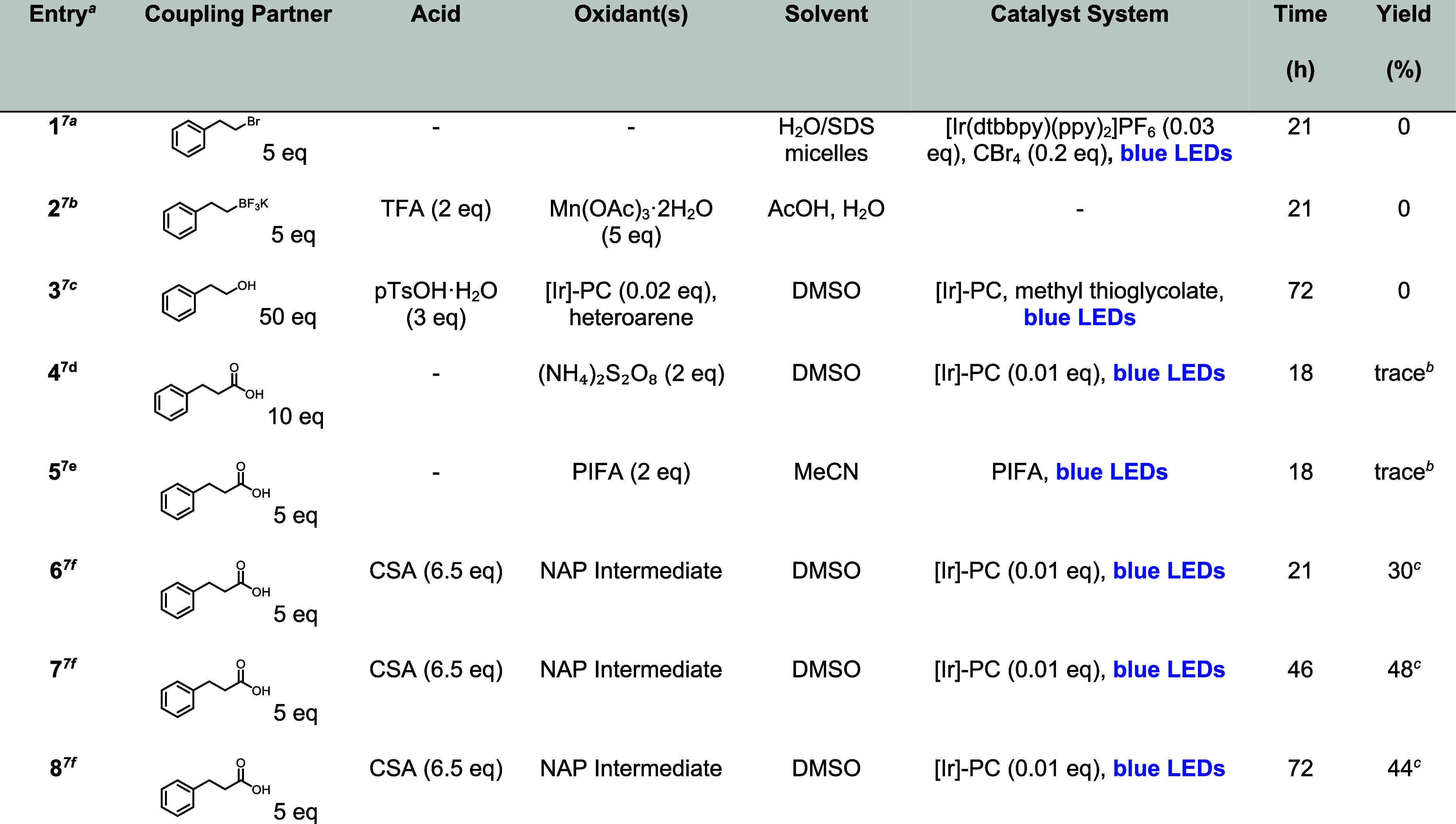
Triple Alkylation of 3-Cyanopyridine (**5**) to Give 2,4,6-Triphenethylnicotinonitrile (**7**) Using Known Minisci-Type Alkylation Strategies

a See the Supporting Information for additional experimental details.

b Trace amounts of product observable by LCMS.

c Isolated yield after column chromatography.

A wide variety of known (and highly robust) conditions^7b-e^ also failed to give the desired product (or gave only trace product) in this context, although in many cases appreciable amounts of mono and disubstituted products were observed (even despite prolonged reaction times and large excesses of coupling partners/reagents; see Table 1 and the Supporting Information for further details). However, we were ultimately gratified to find that modified conditions described in a recent report by Sherwood and colleagues,^7f^ which utilizes radical species derived from *in situ*-generated *N*-(acyloxy)phthalimides (NAPs), gave desired tetrasubstituted pyridine **7** in 30% yield after stirring with the (Ir[dF(CF_3_)ppy]_2_(dtbpy))PF_6_ photocatalyst ([Ir]-PC) under blue LEDs for 21 h (Table 1, entry 6). We were encouraged by the fact that a one-pot installation of three pendants could be achieved in appreciable yield and were further gratified to find that extension of the reaction time (46 and 72 h) was beneficial (48% and 44% yields respectively, Table 1). Excitingly, these conditions also proved successful using 3-bromopyridine (**9**) as a starting material, as well as methyl ester **11** (the former is an unprecedented example of a triple sp^3^-sp^2^ C–H functionalization around a halogenated heteroarene). Additionally, these conditions could also be leveraged toward the synthesis of a pentasubstituted pyridine (**14**), albeit in diminished yield (Scheme 1). We envisioned that the resulting tetrasubstituted intermediates could be used as a handle by which to access one or more of the diarylheptanoid natural products.

**Scheme 1:**
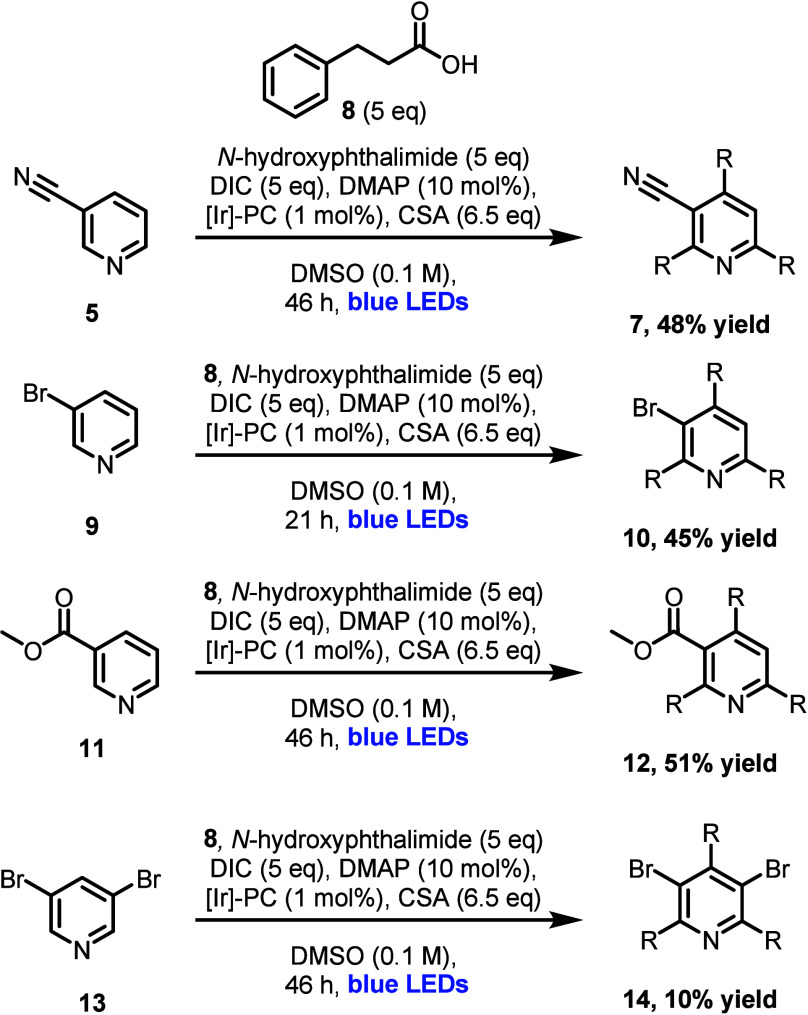
Synthesis of Pyridines **7**, **10**, **12**, and **14** via *N*-(Acyloxy)phthalimides (NAPs) R = CH_2_CH_2_Ph.

All initial efforts to complete the synthesis of alpinidinoid C (**4**) via ketone synthesis from cyanopyridine **7** were unsuccessful. Grignard chemistry, even under highly forcing conditions, returned only starting material or trace amounts of **4**. Attempted reactions starting from bromopyridine **10** were similarly challenging; no evidence of natural product **4** under both Weinreb-Nahm ketone synthesis and direct C–H activation photoredox conditions^8^ was observed. Surmising that the steric encumbrance afforded by the flanking phenethyl substituents was limiting reactivity, we decided to revise our synthetic strategy toward alpinidinoid C (**4**) by inverting the synthetic sequence. A Grignard reaction of 3-cyanopyridine **5** proved successful, affording known ketone **16** in 42% yield.^9^ We were delighted to find that subjection of **16** to the *in situ*-generated NAP conditions proved successful; alpinidinoid C (**4**) was ultimately isolated in 19% yield (see Scheme 2). The spectral and analytical data for **4** matched those of the natural isolate in all respects (see the Supporting Information). Although this yield is modest, this route afforded 36 mg of pure alpinidinoid C (**4**) after only 2 steps, a marked improvement on the initial isolation from the natural source (4.5 mg of **4** was isolated from ∼20 kg of plant matter).^4,10^ Along with **4**, the 4,6-dialkylated (**17**) and 2,6-dialkylated products (**18**) were isolated in 21% and 15% yields, respectively. Both dialkylated products were resubjected to the standard reaction conditions to give **4** in 8−24% yield (Scheme 2).

**Scheme 2:**
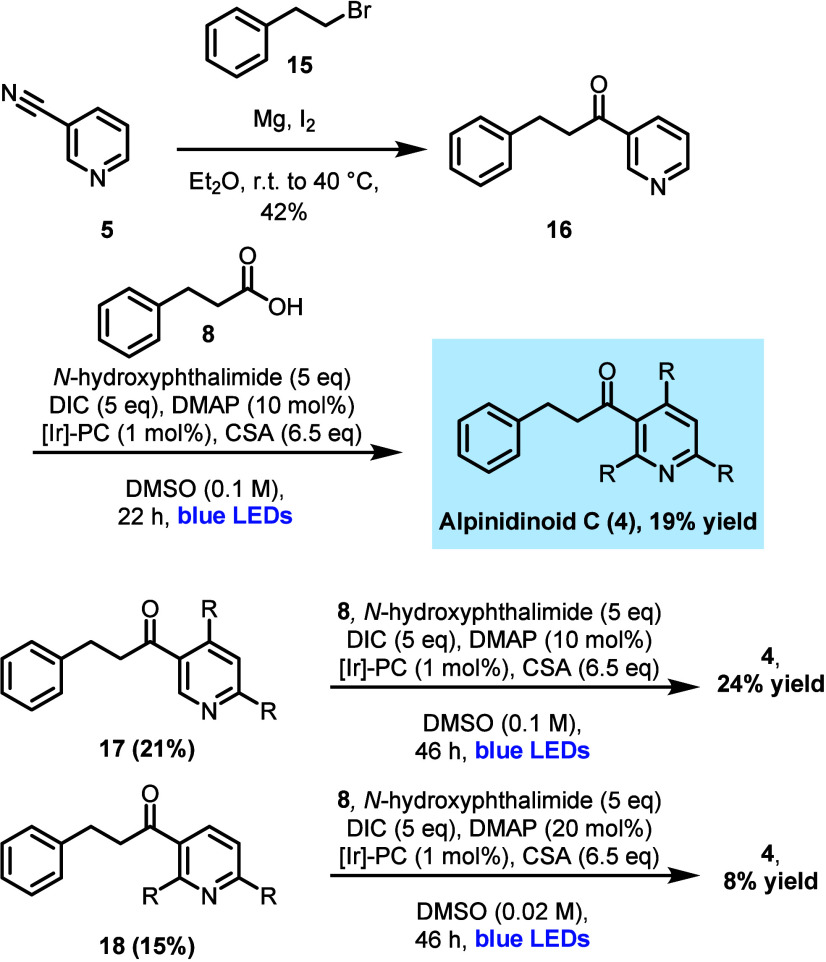
Synthesis of Alpinidinoid C (**4**) R = CH_2_CH_2_Ph.

Toward the synthesis of officinine B (**3**), bromopyridine **10** was subjected to the Mizoroki-Heck conditions described by Namsa-aid and Ruchirawat,^11^ using widely and cheaply available eugenol (**19**) as a coupling partner. Although a successful coupling was observed by LCMS (overlapping peaks with diagnostic *m*/*z* = 554), all attempts to separate the resulting isomeric mixture of products were unfortunately unsuccessful (Scheme 3A). Indeed, olefin isomerization of these types of 1,3-diarylpropyl systems has been reported under Mizoroki-Heck conditions and can significantly impede product isolation.^12^ Attempts to suppress this isomerization through the use of silver salts^13^ were also unfruitful.

**Scheme 3:**
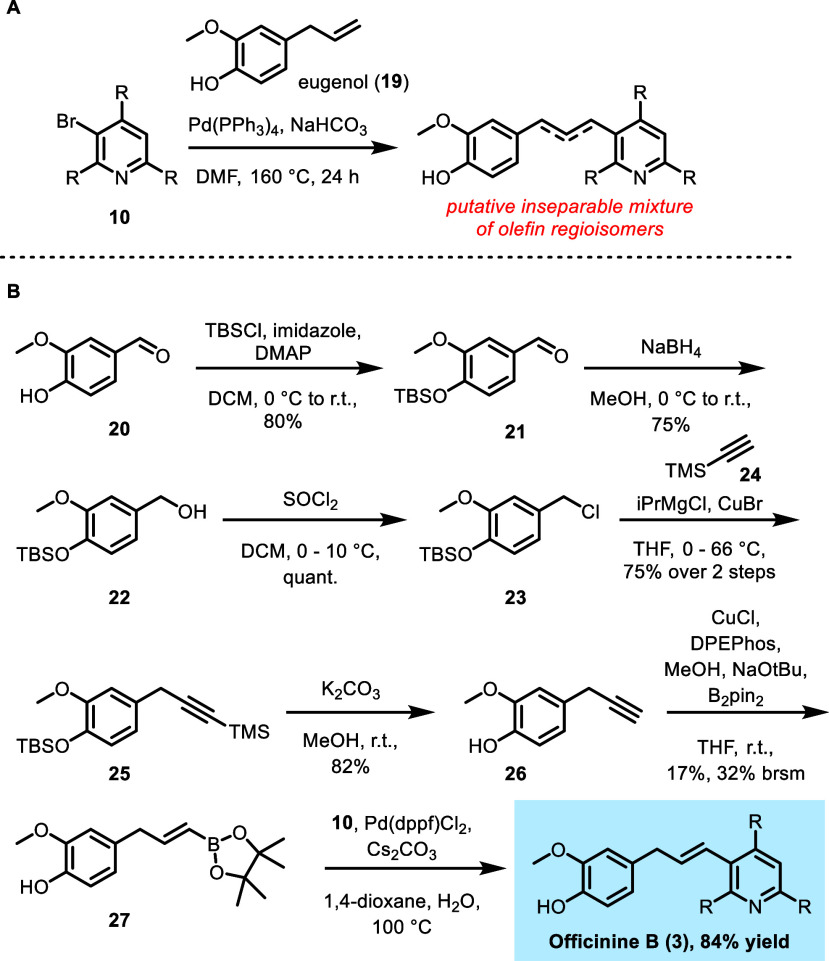
Synthesis of Officinine B (**3**) R = CH_2_CH_2_Ph. (A) Mizoroki-Heck reaction of **10** and **19** gave an inseparable mixture of putative olefin isomers. (B) Synthesis of officinine B (**3**) using a Suzuki-Miyaura cross-coupling approach.

Surmising that an alternative Suzuki-Miyaura cross-coupling approach would circumvent olefin isomerization, we next turned our attention to the synthesis of an *E*-alkenyl boronic ester derivative of eugenol (Scheme 3B). Vanillin (**20**) was first protected as the TBS ether to give aldehyde **21** and reduced to give benzylic alcohol **22**. Compound **22** was then converted to benzyl chloride **23**, which was subsequently converted to TMS alkyne **25** with iPrMgCl and CuBr in high yield over the 4-step sequence.^14^ Global silyl deprotection yielded alkyne **26**, which was converted to pinacol boronic ester **27** as the *E* isomer.^15^ As an alternative route, eugenol (**19**) can also first be protected as the TBS ether, followed by Upjohn dihydroxylation/oxidation, and boronic ester synthesis via boron-Wittig reaction (see the Supporting Information for full experimental details).

At this stage, **27** was smoothly coupled to tetrasubstituted bromopyridine **10** to give officinine B (**3**) in 84% yield, with no apparent olefin isomerization byproducts. The spectral and analytical data for **3** matched those of the natural isolate in all respects (see the Supporting Information). This revised sequence afforded 20 mg of pure officinine B (**3**), an improvement on the 5 mg of compound isolated from ∼17 g of raw plant matter.^5b^

In conclusion, we have accomplished the first syntheses of alpinidinoid C (**4**) and officinine B (**3**) using a blue-light-mediated Minisci-type reaction approach. A one-pot, triple C–H alkylation strategy from a simple pyridine precursor allowed for the rapid synthesis of these dimeric diarylheptanoids and is a rare example of heteroarene polyalkylation using a primary radical species. Although examples exist which use this type of NAP-mediated approach to create semisynthetic natural products via late-stage derivatization,^7f,16^ the syntheses described herein are among very few reports which utilize this methodology toward the synthesis of a natural product itself (particularly with respect to heteroarene functionalization).^16b,17^ The synthesis of alpinidinoid C (**4**) is also noteworthy for its brevity (2 total steps toward a densely and asymmetrically functionalized pyridine), and represents 100% ideality as defined by Gaich and Baran (all synthetic steps construct C–C skeletal bonds).^18^ It is our hope that these concise routes toward alpinidinoid C (**4**) and officinine B (**3**) will enable a more complete understanding of the pharmacology of these and related diarylheptanoids, and reveal possible opportunities for medicinal chemistry. 2,4,6-Trisubstituted pyridines have also found applications in contexts outside of the diarylheptanoid natural products. Structures of this type are known to be useful iridium chelators for organic luminescent devices, have applications in fluorous phase chemistry, and can transport protons across bilayer membranes.^19^ Follow up studies around this broadly applicable methodology are ongoing in our laboratory and will be reported in due course.

## Data Availability

The data underlying this study are available in the published article and its Supporting Information.

## Supporting Information Available

Experimental procedures and NMR spectroscopy data (PDF)
