# Supplementary material for: Synthesis of the Dimeric Diarylheptanoids Alpinidinoid C and Officinine B Enabled by Blue-Light-Mediated Triple-Minisci-Type Alkylation
Source: Org Lett. 2024 Oct 10;26(42):9028–33. doi: 10.1021/acs.orglett.4c03227 (PMC11519911; doi:10.1021/acs.orglett.4c03227)

Supporting Information

Synthesis of the Dimeric Diarylheptanoids Alpinidinoid C and Officinine B Enabled by Blue-Light-Mediated Triple-Minisci-Type Alkylation

Daniel C. Schultz, Upendra Rathnayake, Renn A. Duncan, Alexa E. Richardson, Aaron M. Bender [*]

*Corresponding author email: aaron.bender@vanderbilt.edu

Warren Center for Neuroscience Drug Discovery and Department of Pharmacology, Vanderbilt University, Nashville, Tennessee 37232, United States

Chemistry **S2**

General Experimental Procedures S2

Conditions Screen for Triple-Minisci-Type Alkylation S3

Synthesis of Intermediates **7**, **10**, **12** and **14** S6

Synthesis of Natural Product Alpinidinoid C (**4**) S10

Synthesis of Natural Product Officinine B (**3**) (Route 1) S14

Synthesis of Natural Product Officinine B (**3**) (Route 2) S18

Copies of NMR Spectra S22

General Experimental Procedures

All reactions were carried out employing standard chemical techniques. Solvents used for reactions and extraction were ACS grade, and HPLC grade solvents were used for purification. All reagents were purchased from commercial sources and were used without further purification. Photocatalysts $[Ir(dtbbpy)(ppy)_2]PF_6$ (CAS 676525-77-2) and $(Ir[dF(CF_3)ppy]_2(dtbpy))PF_6$ (870987-63-6; [Ir]-PC) were purchased from Strem Chemicals. For all reactions requiring heat, a heating block equipped with temperature control was used for reaction setup.

All NMR spectra were recorded on a 400 MHz Bruker AV-400 instrument. ^1H chemical shifts are reported as δ values in ppm relative to the residual solvent peak ($CDCl_3$ = 7.26, CD_3CN = 1.94, Acetone-d_6 = 2.05). Data are reported as follows: chemical shift, multiplicity (br = broad, s = singlet, d = doublet, t = triplet, q = quartet, p = pentet, dd = doublet of doublets, ddd = doublet of doublet of doublets, td = triplet of doublets, m = multiplet), coupling constant, and integration. ^{13}C chemical shifts are reported as δ values in ppm relative to the residual solvent peak ($CDCl_3$ = 77.16, CD_3CN = 1.32, Acetone-d_6 = 29.84).

LCMS data were obtained on a Waters QDa (Performance) SQ MS with ESI source. MS parameters were as follows: cone voltage: 15 V, capillary voltage: 0.8 kV, probe temperature: 600° C. Samples were introduced via an Acquity I-Class PLUS UPLC comprised of a BSM, FLSM, CH-A, and PDA. UV absorption was generally observed at 215 nm and 254 nm; 4 nm bandwidth. Column: Phenomenex EVO C18, 1.0 x 50 mm, 1.7 um. Column temperature: 55° C. Flow rate: 0.4 mL/min. Default gradient: 5% to 95% CH_3CN (0.05% TFA) in water (0.05% TFA) over 1.4 min, hold at 95% CH_3CN for 0.1 min.

High resolution mass spectra were obtained on an Agilent 6540 UHD Q-TOF with ESI source. MS parameters were as follows: fragmentor: 150, capillary voltage: 3500 V, nebulizer pressure: 60 psig, drying gas flow: 13 L/min, drying gas temperature: 275° C. Samples were introduced via an Agilent 1290 UHPLC comprised of a G4220A binary pump, G4226A ALS, G1316C TCC, and G4212A DAD with ULD flow cell. UV absorption was observed at 215 nm and 254 nm with a 4 nm bandwidth. Column: Agilent Zorbax Extend C18, 1.8 µm, 2.1 x 50 mm. Gradient conditions: 5% to 95% CH_3CN in water (0.1% formic acid) over 1 min, hold at 95% CH_3CN for 0.1 min, 0.5 mL/min, 40° C.

Automated flash column chromatography was performed on a Biotage Isolera 1 or a Teledyne ISCO CombiFlash system.

Melting points were recorded on an OptiMelt automated melting point system by Stanford Research Systems.

Blue light-mediated chemistry was conducted using the EvoluChemTM PhotoRedOx Box equipped with Kessil LED lamp (HepatoChem HCK1006-01-016). LED specifications: Kessil product A160WE (Tuna Blue, 40W power consumption, 10,000-20,000K spectrum range). Reactions were conducted in borosilicate glass approximately 6 cm away from light source under maximum intensity setting without external heating; internal temperature typically ~ 46 °C).

***Safety statement**: No unexpected or unusually high safety hazards were encountered. LiTMP is pyrophoric and should be handled with the appropriate care.*

Conditions Screen for Triple-Minisci-Type Alkylation

Table S1. Full Conditions Screen for Triple-Minisci-Type Alkylation of Nicotinonitrile

Entry[a]	Coupling Partner	Acid	Oxidant(s)	Solvent	Catalyst System	Time (h)	Yield (%)
1	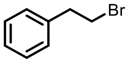	-	-	water/SDS micelles	[Ir(dtbbpy)(ppy)$_2$]PF$_6$, CBr$_4$, **blue LEDs**	21	0
2	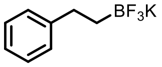	TFA	Mn(OAc)$_3$·2H$_2$O	AcOH, water	-	21	0
3	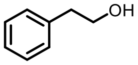	pTsOH·H$_2$O	[Ir]-PC, heteroarene	DMSO	[Ir]-PC, methyl thioglycolate, **blue LEDs**	72	0
4	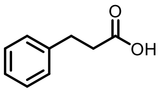	-	$(NH_4)_2S_2O_8$	DMSO	[Ir]-PC, **blue LEDs**	18	trace
5	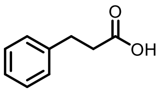	-	PIFA	MeCN	PIFA, **blue LEDs**	18	trace
6	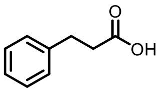	CSA	NAP Intermediate	DMSO	[Ir]-PC, **blue LEDs**	21	30
7	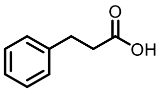	CSA	NAP Intermediate	DMSO	[Ir]-PC, **blue LEDs**	46	48
8	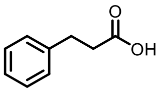	CSA	NAP Intermediate	DMSO	[Ir]-PC, **blue LEDs**	72	44

Entry 1.[1] Nicotinonitrile (30 mg, 0.29 mmol, 1.0 eq), carbon tetrabromide (19 mg, 0.058 mmol, 0.2 eq), [Ir(dtbbpy)(ppy)$_2$]PF$_6$ (7.9 mg, 0.009 mmol, 0.03 eq, CAS 676525-77-2), and sodium dodecyl sulfate (208 mg, 0.72 mmol, 2.5 eq) were combined in a vial, which was sealed and placed under an inert atmosphere. Water (14 mL) was then added via syringe, and the resulting reaction mixture was stirred under vacuum for ~5 min. (2-Bromoethyl)benzene (0.20 mL, 1.44 mmol, 5.0 eq) was then added via syringe. The resulting reaction mixture was stirred under an N_2 atmosphere under blue LED irradiation for 21 h, after which time LCMS indicated no evidence of desired product formation. LCMS analysis of crude reaction mixture:

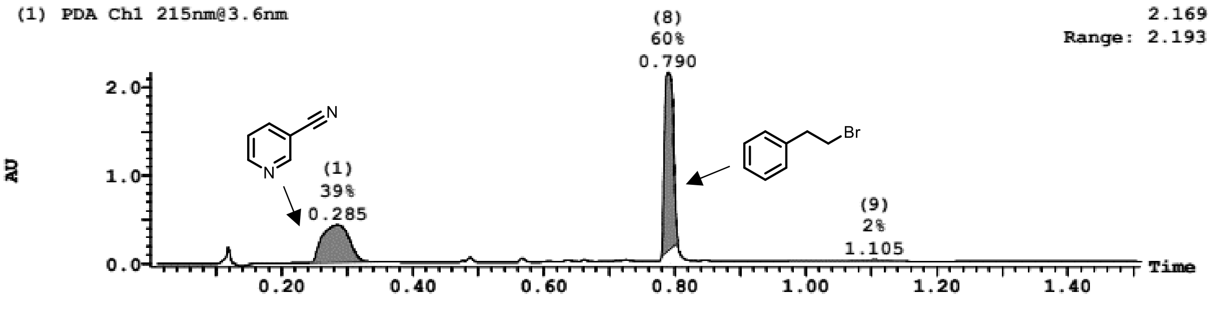

Entry 2.[2] Nicotinonitrile (30 mg, 0.29 mmol, 1.0 eq) and potassium phenethyltrifluoroborate (306 mg, 1.44 mmol, 5.0 eq) were combined in AcOH (2 mL) and water (2 mL), and TFA (0.044 mL, 0.58 mmol, 2 eq) was added. The resulting mixture was stirred and

sonicated until it became homogenous, after which time $Mn(OAc)_3 \cdot 2H_2O$ (391 mg, 1.44 mmol, 5.0 eq) was added. The resulting reaction mixture was stirred at 50 °C for 21 h, after which time LCMS indicated no evidence of desired product formation. LCMS analysis of crude reaction mixture:

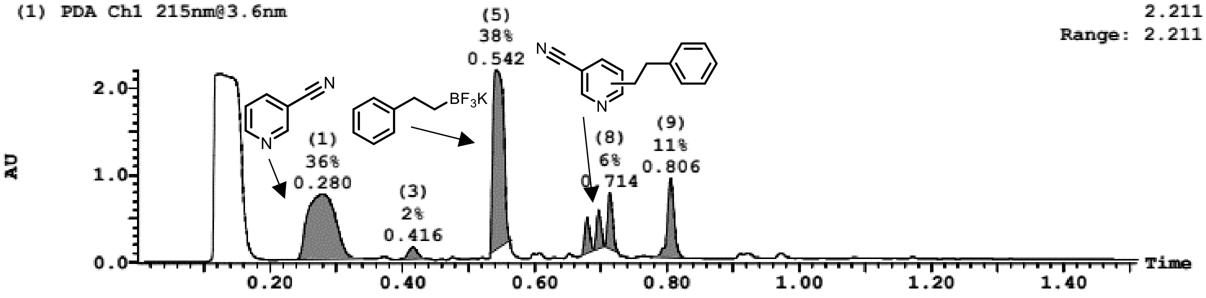

Entry 3.[3] Nicotinonitrile (30 mg, 0.29 mmol, 1.0 eq), p-TsOH·H_2O (164 mg, 0.86 mmol, 3.0 eq), $(Ir[dF(CF_3)ppy]_2(dtbpy))PF_6$ (6.5 mg, 0.006 mmol, 0.02 eq, CAS 870987-63-6), and 2-phenylethan-1-ol (1.7 mL, 14.4 mmol, 50.0 eq) were combined in DMSO (2.9 mL), and the resulting mixture was placed briefly under vacuum, and then degassed by bubbling N_2 through solution for ~10 min. Methyl thioglycolate (4 drops) was then added. The resulting reaction mixture was sealed with Parafilm and stirred under blue LED irradiation for 3 days, after which time LCMS indicated no evidence of desired product formation. LCMS analysis of crude reaction mixture:

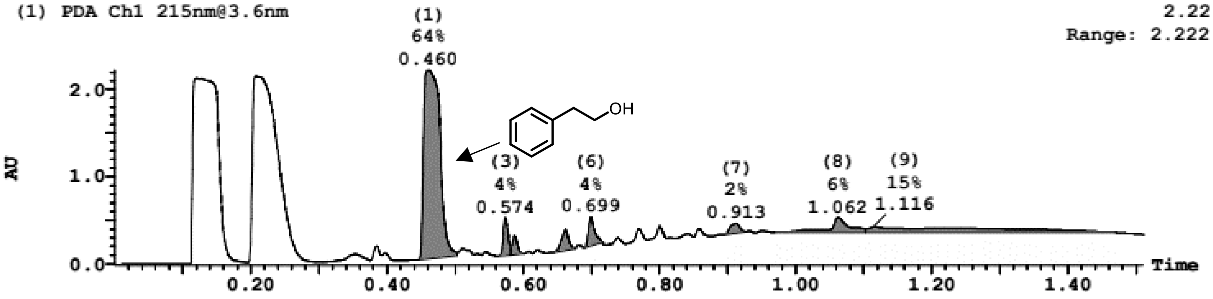

Entry 4.[4] Nicotinonitrile (30 mg, 0.29 mmol, 1.0 eq), 3-phenylpropanoic acid (433 mg, 2.9 mmol, 10.0 eq), ammonium persulfate (132 mg, 0.58 mmol, 2.0 eq) and $(Ir[dF(CF_3)ppy]_2(dtbpy))PF_6$ (3.2 mg, 0.003 mmol, 0.01 eq, CAS 870987-63-6) were combined in a vial, which was sealed and placed under an N_2 atmosphere. DMSO (2.9 mL) was then added, and the vial was sealed with Parafilm. The resulting reaction mixture was stirred under blue LED irradiation for 18 h, after which time LCMS indicated trace formation of desired triple alkylated product. LCMS analysis of crude reaction mixture:

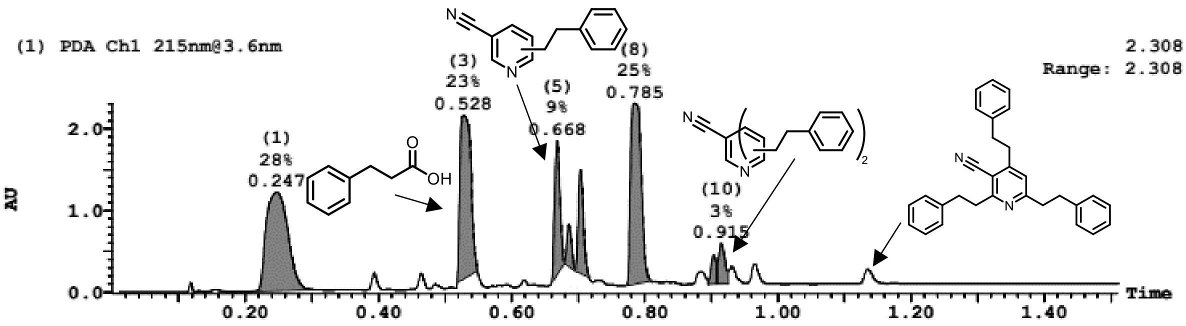

Entry 5.[5] Nicotinonitrile (30 mg, 0.29 mmol, 1.0 eq), 3-phenylpropanoic acid (216 mg, 1.44 mmol, 5.0 eq), and PIFA (248 mg, 0.58 mmol, 2.0 eq) were combined in a vial, which was sealed and placed under an N_2 atmosphere. MeCN (2.9 mL) was then added, and the vial was sealed with Parafilm. The resulting reaction mixture was stirred under blue LED irradiation for 18 h, after which time LCMS indicated trace formation of desired triple alkylated product. LCMS analysis of crude reaction mixture:

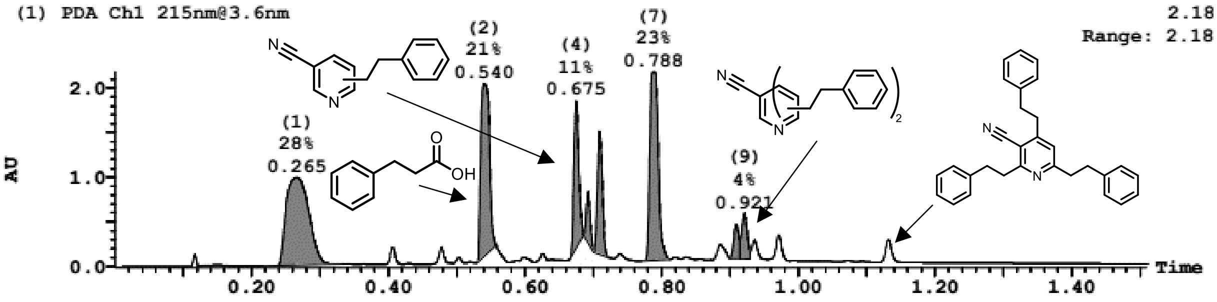

References

1. Santos, M. S.; Cybularczyk-Cecotka, M.; König, B.; Giedyk, M. Minisci C-H Alkylation of Heteroarenes Enabled by Dual Photoredox/Bromide Catalysis in Micellar Solutions. *Chem. Eur. J.* **2020**, *26*, 15323-15329.

2. Molander, G. A.; Colombel, V.; Braz, V. A. Direct Alkylation of Heteroaryls Using Potassium Alkyl- and Alkoxymethyltrifluoroborates. *Org. Lett*. **2011**, *13*, 1852-1855.

3. Jin, J.; MacMillan, D. W. C. Alcohols as Alkylating Agents in Heteroarene C-H Functionalization. *Nature* **2015**, *525*, 87-90.

4. Garza-Sanchez, R. A.; Tlahuext-Aca, A.; Tavakoli, G.; Glorius, F. Visible Light-Mediated Direct Decarboxylative C-H Functionalization of Heteroarenes. *ACS Catal*. **2017**, *7*, 4057-4061.

5. Zhang, X.-Y.; Weng, W.-Z.; Liang, H.; Yang, H.; Zhang, B. Visible-Light-Initiated, Photocatalyst-Free Decarboxylative Coupling of Carboxylic Acids with *N*-Heterocycles. *Org. Lett*. **2018**, *20*, 4686-4690.

Synthesis of Intermediates 7, 10, 12 and 14

Reactions were conducted using a modified procedure as described by Sherwood *et al*.[1]

2,4,6-Triphenethylnicotinonitrile (7)

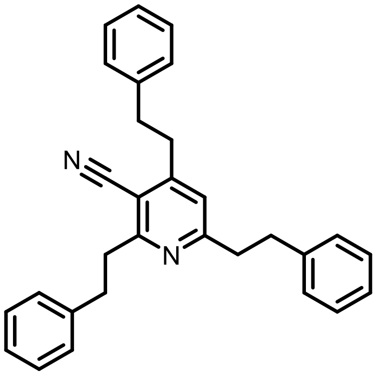

To a vial was added 3-phenylpropanoic acid (238 mg, 1.59 mmol, 5.0 eq), *N*-hydroxyphthalimide (260 mg, 1.58 mmol, 5.0 eq), and 4-dimethylaminopyridine (4.2 mg, 34.4 µmol, 0.1 eq), which were dissolved in 1.6 mL anhydrous DMSO. The solution was degassed with N_2 for 4 min, then *N*,*N'*-diisopropylcarbodiimide (245 µL, 1.58 mmol, 5.0 eq) was added. The mixture was stirred at r.t. for 21 h. In a separate vial was prepared a solution of 10-camphor-10-sulfonic acid (β) (479 mg, 2.06 mmol, 6.5 eq), nicotinonitrile (33.1 mg, 0.32 mmol, 1.0 eq), and (Ir[dF(CF_3)ppy]$_2$(dtbpy))PF_6 (3.8 mg, 3.38 µmol, 0.01 eq) in 1.0 mL anhydrous DMSO. This solution was transferred to the initial reaction mixture, with 0.6 mL anhydrous DMSO used to wash the vial. The mixture was degassed with N_2 for 4 min, then the vial was sealed and irradiated under blue LED light at maximum intensity for 46 h. The mixture was then diluted with 25 mL DCM, washed with 2x25 mL portions of sat. $NaHCO_3$ solution, and concentrated. The crude material was purified via reverse-phase automated flash column chromatography (Teledyne ISCO, 50 g HPC18 RediSep Rf Gold Column, liquid loading with DMSO, 40-90% MeCN in 0.5 mL/L aqueous NH_4OH). The desired fractions were combined and concentrated, then diluted with sat. $NaHCO_3$ solution and extracted multiple times with DCM. The organic layers were combined and concentrated to afford the title compound as a clear oil (64.0 mg, 48% yield).

^{1}H NMR (400 MHz, CD_3CN) δ 7.30 – 7.23 (m, 6H), 7.22 – 7.09 (m, 9H), 6.92 (s, 1H), 3.24 – 3.19 (m, 2H), 3.07 – 3.00 (m, 4H), 3.00 – 2.94 (m, 4H), 2.87 – 2.81 (m, 2H).

^{13}C NMR (101 MHz, CD_3CN) δ 165.2, 164.6, 155.4, 142.3, 142.0, 141.3, 129.5, 129.5, 129.4, 129.4, 129.3, 127.3, 127.0, 127.0, 122.1, 117.1, 107.6, 40.5, 39.4, 36.8, 36.5, 35.7, 35.5. Note: one quaternary C missing (likely buried under CH signals).

HRMS (TOF, ESI) $[M+H]^+$ calcd for $C_{30}H_{29}N_2$ 417.2325; found 417.2333.

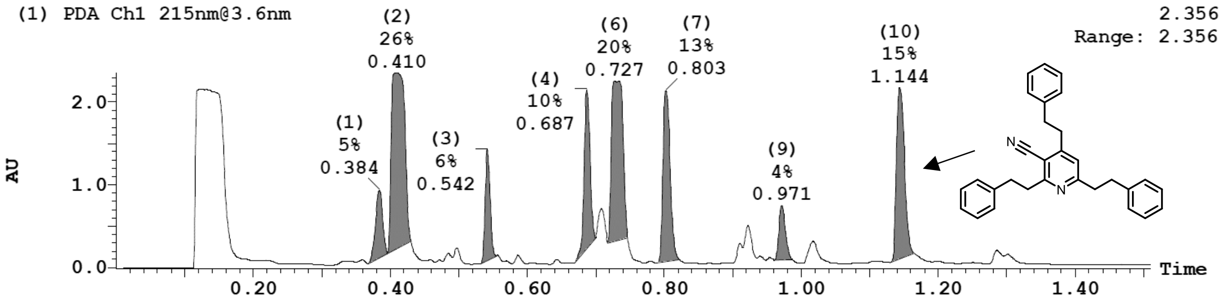

3-Bromo-2,4,6-triphenethylpyridine (10)

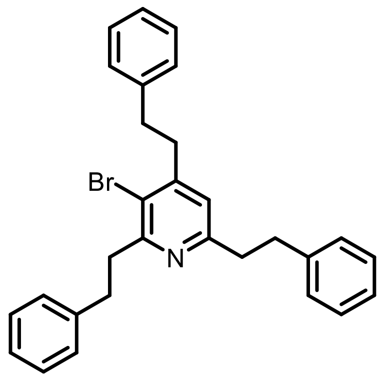

To a vial was added 3-phenylpropanoic acid (239 mg, 1.59 mmol, 5.0 eq), *N*-hydroxyphthalimide (261 mg, 1.60 mmol, 5.0 eq), and 4-dimethylaminopyridine (4.4 mg, 36.0 µmol, 0.1 eq), which were dissolved in 1.6 mL anhydrous DMSO. The solution was degassed with N_2 for 4 min, then *N*,*N'*-diisopropylcarbodiimide (245 µL, 1.58 mmol, 5.0 eq) was added. The mixture was stirred at r.t. for 21 h. In a separate vial was prepared a solution of camphor-10-sulfonic acid (β) (477 mg, 2.05 mmol, 6.5 eq), 3-bromopyridine (30.9 µL, 0.32 mmol, 1.0 eq), and (Ir[dF(CF_3)ppy]$_2$(dtbpy))PF_6 (3.6 mg, 3.21 µmol, 0.01 eq) in 1.0 mL anhydrous DMSO. This solution was transferred to the initial reaction mixture, with 0.6 mL anhydrous DMSO used to wash the vial. The mixture was degassed with N_2 for 4 min, then the vial was sealed and irradiated under blue LED light at maximum intensity for 21 h. The mixture was then diluted with 25 mL DCM, washed with 2x25 mL portions of sat. $NaHCO_3$ solution, and concentrated. The crude material was purified via reverse-phase automated flash column chromatography (Teledyne ISCO, 50 g HPC18 RediSep Rf Gold Column, liquid loading with DMSO, 40-95% MeCN in 0.5 mL/L aqueous NH_4OH) to afford the title compound as a golden-yellow oil that becomes a gel upon standing (66.6 mg, 45% yield). *On a 1 mmol scale with 48 h reaction time under otherwise identical reaction conditions, the title compound was isolated in 39% yield.*

^{1}H NMR (400 MHz, CD_3CN) δ 7.28 – 7.17 (m, 8H), 7.17 – 7.09 (m, 7H), 6.79 (s, 1H), 3.21 - 3.15 (m, 2H), 3.01 - 2.95 (m, 2H), 2.93 – 2.85 (m, 6H), 2.81 – 2.75 (m, 2H).

^{13}C NMR (101 MHz, CD_3CN) δ 159.9, 159.6, 151.1, 142.8, 142.7, 142.0, 129.49, 129.46, 129.4, 129.3, 129.3, 127.1, 126.9, 126.8, 123.6, 121.4, 40.5, 39.7, 38.9, 36.1, 35.8, 35.0. Note: One quaternary C missing (likely buried under CH signals).

HRMS (TOF, ESI) $[M+H]^+$ calcd for $C_{29}H_{29}BrN$ 470.1478; found 470.1484.

Methyl 2,4,6-triphenethylnicotinate (12)

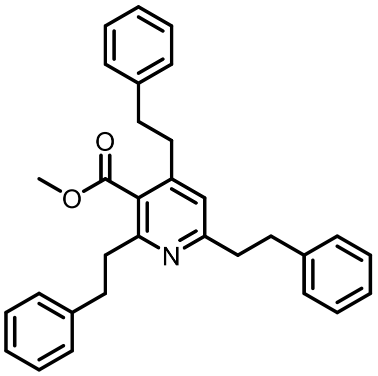

To a vial was added 3-phenylpropanoic acid (235 mg, 1.56 mmol, 5.0 eq), *N*-hydroxyphthalimide (255 mg, 1.56 mmol, 5.0 eq), and 4-dimethylaminopyridine (4.0 mg, 32.7 µmol, 0.1 eq), which were dissolved in 1.6 mL anhydrous DMSO. The solution was degassed with N_2 for 4 min, then *N*,*N'*-diisopropylcarbodiimide (242 µL, 1.56 mmol, 5.0 eq) was added. The mixture was stirred at r.t. for 21 h. In a separate vial was prepared a solution of 10-camphor-10-sulfonic acid (β) (475 mg, 2.05 mmol, 6.5 eq), methyl pyridine-3-carboxylate (43.9 mg, 0.32 mmol, 1.0 eq), and (Ir[dF(CF_3)ppy]$_2$(dtbpy))PF_6 (4.0 mg, 3.6 µmol, 0.01 eq) in 1.0 mL anhydrous DMSO. This solution was transferred to the initial reaction mixture, with 0.6 mL anhydrous DMSO used to wash the vial. The mixture was degassed with N_2 for 4 min, then the vial was sealed and irradiated under blue LED light at maximum intensity for 46 h. The mixture was then diluted with 25 mL DCM, washed with 2x25 mL portions of sat. $NaHCO_3$ solution, and concentrated. The crude material was purified via reverse-phase automated flash column chromatography (Teledyne ISCO, 50 g HPC18 RediSep Rf Gold Column, liquid loading with DMSO, 50-90% MeCN in 0.5 mL/L aqueous NH_4OH). The desired fractions were combined and concentrated, then diluted with sat. $NaHCO_3$ solution and extracted multiple times with 3:1 $CHCl_3$/IPA. The organic layers were combined and concentrated to afford the title compound as a pale yellow oil (73.6 mg, 51% yield).
^{1}H NMR (400 MHz, CD_3CN) δ 7.30 – 7.23 (m, 6H), 7.22 – 7.12 (m, 9H), 6.87 (s, 1H), 3.86 (s, 3H), 3.04 – 2.96 (m, 8H), 2.80 – 2.76 (m, 4H).
^{13}C NMR (101 MHz, CD_3CN) δ 170.2, 162.5, 158.0, 149.4, 142.8, 142.7, 142.1, 129.5, 129.4, 129.4, 129.4, 129.3, 129.3, 127.5, 127.1, 126.7, 122.3, 53.0, 40.2, 38.9, 37.3, 36.2, 36.0, 36.0. Note: one quaternary C missing (likely buried under CH signals).
HRMS (TOF, ESI) $[M+H]^+$ calcd for $C_{31}H_{32}NO_2$ 450.2428; found 450.2426.

3,5-Dibromo-2,4,6-triphenethylpyridine (14)

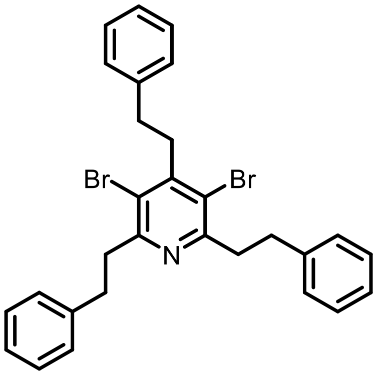

To a vial was added 3-phenylpropanoic acid (234 mg, 1.55 mmol, 5.0 eq), *N*-hydroxyphthalimide (255 mg, 1.56 mmol, 5.0 eq), and 4-dimethylaminopyridine (4.0 mg, 32.7 µmol, 0.1 eq), which were dissolved in 1.6 mL anhydrous DMSO. The solution was degassed with N_2 for 4 min, then *N*,*N'*-diisopropylcarbodiimide (241 µL, 1.55 mmol, 5.0 eq) was added. The mixture was stirred at r.t. for 21 h. In a separate vial was prepared a solution of 10-camphor-10-sulfonic acid (β) (476 mg, 2.05 mmol, 6.5 eq), 3,5-dibromopyridine (74.8 mg, 0.32 mmol, 1.0 eq), and (Ir[dF(CF_3)ppy]$_2$(dtbpy))PF_6 (4.0 mg, 3.6 µmol, 0.01 eq) in 1.0 mL anhydrous DMSO. This solution was transferred to the initial reaction mixture, with 0.6 mL anhydrous DMSO used to wash the vial. The mixture was degassed with N_2 for 4 min, then the vial was sealed and irradiated under blue LED light at maximum intensity for 46 h. The mixture was then diluted with 25 mL DCM, washed with 2x25 mL portions of sat. $NaHCO_3$ solution, and concentrated. The crude material was purified via reverse-phase automated flash column chromatography (Teledyne ISCO, 30 g HPC18 RediSep Rf Gold Column, liquid loading with DMSO, 50-100% MeCN in 0.5 mL/L aqueous NH_4OH). The desired fractions were combined and concentrated to afford the title compound as a pale yellow solid (17.7 mg, 0.032 mmol, 10% yield).

^{1}H NMR (400 MHz, Acetone-*d$_6$*) δ 7.36 – 7.22 (m, 13H), 7.22 – 7.16 (m, 2H), 3.35 – 3.29 (m, 2H), 3.29 – 3.23 (m, 4H), 3.08 – 3.02 (m, 4H), 2.86 – 2.77 (m, 2H).

^{13}C NMR (101 MHz, Acetone-*d$_6$*) δ 158.56, 150.09, 142.55, 141.71, 129.55, 129.47, 129.33, 129.28, 127.37, 126.94, 121.64, 40.92, 40.37, 34.85, 34.22.

HRMS (TOF, ESI) $[M+H]^+$ calcd for $C_{29}H_{28}Br_2N$ 548.0583; found 548.0578.

Melting point: 83 – 85 °C

References

1. Sherwood, T. C.; Li, N.; Yazdani, A. N.; Dhar, T. G. M. Organocatalyzed, Visible-Light Photoredox-Mediated, One-Pot Minisci Reaction Using Carboxylic Acids via *N*-(Acyloxy)phthalimides. *J. Org. Chem*. **2018**, *83*, 3000-3012.

Synthesis of Natural Product Alpinidinoid C (4)

3-Phenyl-1-(pyridin-3-yl)propan-1-one (16)

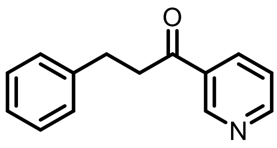

To an oven dried scintillation vial, freshly ground Mg turnings (330 mg, 13.6 mmol, 14.0 eq) and a fleck of I_2 were added under an N_2 atmosphere. The vial was gently heated with a heat gun until the I_2 partially sublimed and then gradually cooled back to r.t. To this vial a solution of (2-bromoethyl)benzene (500 mg, 2.70 mmol, 2.8 eq) in dry diethyl ether (0.75 mL) was added, and the resulting solution was stirred for 3 h at r.t. Then, the formed Grignard reagent was carefully transferred to a solution of nicotinonitrile (100 mg, 0.96 mmol, 1.0 eq) in dry diethyl ether (1.25 mL) under N_2. The resulting solution was heated to 40 °C and stirred until the nicotinonitrile was completely consumed by LCMS. Upon completion, the reaction was cooled to 0 °C, and quenched with saturated aqueous NH_4Cl. The organic layer was separated, and the aqueous layer was extracted with DCM (2x10 mL). The combined organic layers were dried over Na_2SO_4, concentrated under vacuum, and purified via normal phase chromatography on silica gel (0-100% EtOAc in hexanes) to afford the title compound as a colorless oil (86 mg, 42% yield).
^{1}H NMR (400 MHz, $CDCl_3$) δ 9.16 (dd, *J* = 2.3, 0.9 Hz, 1H), 8.77 (dd, *J* = 4.8, 1.7 Hz, 1H), 8.22 (dt, *J* = 8.0, 2.0 Hz, 1H), 7.41 (ddd, *J* = 8.0, 4.8, 0.9 Hz, 1H), 7.34 – 7.27 (m, 2H), 7.27 – 7.19 (m, 3H), 3.36 – 3.28 (m, 2H), 3.09 (t, *J* = 7.6 Hz, 2H).
^{13}C NMR (101 MHz, $CDCl_3$) δ 198.1, 153.5, 149.6, 140.8, 135.4, 132.1, 128.6, 128.4, 126.3, 123.7, 40.8, 29.8.
HRMS (TOF, ESI) $[M+H]^+$ calcd for $C_{14}H_{14}NO$ 212.1070; found 212.1066.
Note: NMR data align with literature.[1]

Alpinidinoid C (4)

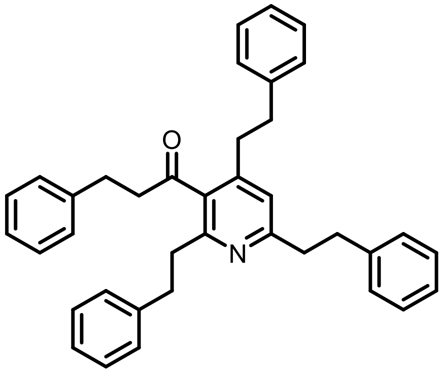

To a vial was added 3-phenylpropanoic acid (275 mg, 1.83 mmol, 5.0 eq), *N*-hydroxyphthalimide (300 mg, 1.84 mmol, 5.0 eq), and 4-dimethylaminopyridine (4.7 mg, 0.039 mmol, 0.1 eq), which were dissolved in anhydrous DMSO (1.8 mL). The solution was degassed with N_2 for 5 min, then *N*,*N'*-diisopropylcarbodiimide (282 µL, 1.82 mmol, 5.0 eq) was added, and the reaction was stirred at r.t. for 21 h. In a separate vial was prepared a solution of camphor-10-sulfonic acid (β) (550.4 mg, 2.37 mmol, 6.5 eq), 3-phenyl-1-(pyridin-3-yl)propan-1-one (77.0 mg, 0.365 mmol, 1.0 eq), and $(Ir[dF(CF_3)ppy]_2(dtbpy))PF_6$ (4.9 mg, 4.37 µmol, 0.01 eq) in 1.3 mL

anhydrous DMSO, which was transferred to the initial reaction, with 0.5 mL anhydrous DMSO used to wash the second vial. The mixture was degassed with N_2 for 5 min, then the vial was sealed and irradiated under blue LED light at maximum intensity for 22 h. The mixture was diluted with 25 mL DCM, washed with 2x25 mL portions of aqueous $NaHCO_3$, and concentrated. The crude material was purified via reverse-phase automated flash column chromatography (Teledyne ISCO, 50 g HPC18 RediSep Rf Gold Column, liquid loading with DMSO, 45-95% MeCN in 0.5 mL/L aqueous NH_4OH) to afford the title compound as a yellow oil that became a gel upon standing (35.9 mg, 19% yield).
^{1}H NMR (400 MHz, Acetone-*d*$_6$) δ 7.30 – 7.20 (m, 12H), 7.20 – 7.12 (m, 4H), 7.12 – 7.06 (m, 4H), 6.97 (s, 1H), 3.07 – 3.01 (m, 6H), 2.99 – 2.93 (m, 2H), 2.87 – 2.76 (m, 6H), 2.67 – 2.60 (m, 2H).
^{13}C NMR (101 MHz, Acetone-*d*$_6$) δ 207.9, 161.4, 155.6, 147.2, 142.7, 142.7, 141.9, 135.8, 129.5, 129.3, 129.3, 129.2, 129.1, 126.9, 126.8, 126.7, 126.7, 121.8, 47.4, 40.4, 38.2, 37.4, 36.2, 36.0, 35.3, 29.87 (assigned by HSQC).
HRMS (TOF, ESI) $[M+H]^+$ calcd for $C_{38}H_{38}NO$ 524.2948; found 524.2944.

All data were in excellent agreement with the natural isolate.[2]

1-(4,6-Diphenethylpyridin-3-yl)-3-phenylpropan-1-one (17)

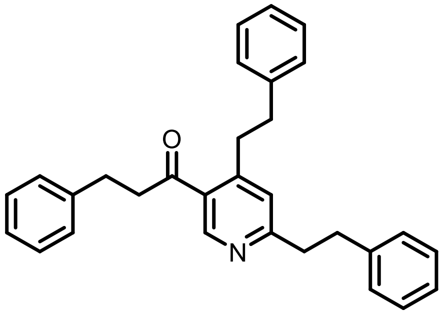

Isolated from the above reaction as a pale yellow gel (32.3 mg, 21% yield).
^{1}H NMR (400 MHz, Acetone-*d*$_6$) δ 8.90 (s, 1H), 7.32 – 7.14 (m, 15H), 7.12 (s, 1H), 3.31 – 3.25 (m, 2H), 3.10 – 2.97 (m, 8H), 2.81 – 2.75 (m, 2H).
^{13}C NMR (101 MHz, Acetone-*d*$_6$) δ 202.3, 164.6, 151.5, 150.4, 142.4, 142.3, 142.2, 132.3, 129.4, 129.3, 129.3, 129.2, 129.2, 129.1, 126.8, 126.8, 126.7, 125.9, 43.7, 40.5, 37.8, 36.0, 36.0, 30.7.
HRMS (TOF, ESI) $[M+H]^+$ calcd for $C_{30}H_{30}NO$ 420.2322; found 420.2320.

1-(2,6-Diphenethylpyridin-3-yl)-3-phenylpropan-1-one (18)

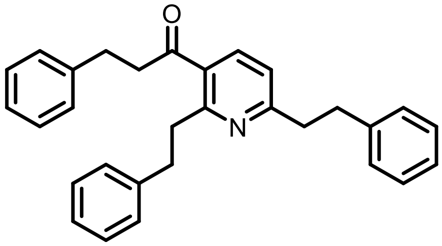

Isolated from the above reaction as a pale yellow oil that became a gel upon standing (22.8 mg, 15% yield).
^{1}H NMR (400 MHz, Acetone-*d*$_6$) δ 7.98 (d, *J* = 8.0 Hz, 1H), 7.30 – 7.21 (m, 10H), 7.21 – 7.14 (m, 5H), 7.12 (d, *J* = 8.0 Hz, 1H), 3.27 – 3.21 (m, 2H), 3.17 – 3.05 (m, 6H), 3.01 – 2.91 (m, 4H).

^{13}C NMR (101 MHz, Acetone-d_6) δ 202.9, 163.8, 160.3, 143.0, 142.5, 142.2, 137.4, 132.1, 129.4, 129.3, 129.3, 129.2, 129.1, 129.1, 126.8, 126.7, 126.6, 120.8, 43.6, 40.4, 38.8, 36.5, 35.9, 30.7. **HRMS (TOF, ESI)** $[M+H]^+$ calcd for $C_{30}H_{30}NO$ 420.2322; found 420.2317.

Synthesis of Alpinidinoid C (4) from Re-subjection of 17 and 18 to Reaction Conditions

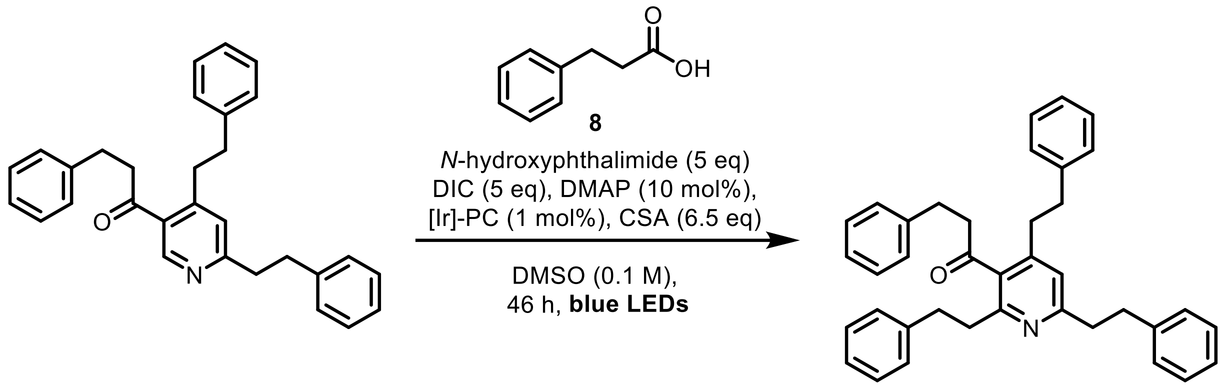

17 **4**

To a vial was added 3-phenylpropanoic acid (53.5 mg, 0.36 mmol, 5.0 eq), 4-dimethylaminopyridine (1.5 mg, 0.01 mmol, 0.1 eq), and *N*-hydroxyphthalimide (58.5 mg, 0.36 mmol, 5.0 eq), which were dissolved in anhydrous DMSO (0.7 mL). The solution was degassed with N_2 for 4 min, then *N*,*N'*-diisopropylcarbodiimide (55 µL, 0.36 mmol, 5.0 eq) was added. The solution was stirred at r.t. for 21 h. To a second vial was added 1-(4,6-diphenethylpyridin-3-yl)-3-phenylpropan-1-one (28.9 mg, 0.07 mmol, 1.0 eq), camphor-10-sulfonic acid (β) (104 mg, 0.45 mmol, 6.5 eq), and $(Ir[dF(CF_3)ppy]_2(dtbpy))PF_6$ (0.77mg, 0.001 mmol, 0.01 eq) in anhydrous DMSO (0.5 mL). The solution was sonicated before adding to the initial reaction, with 0.3 mL anhydrous DMSO used to wash the second vial. The solution was degassed with N_2 for 4 min, then the vial was re-capped and irradiated under blue LED light at maximum intensity for 46 h. Upon completion, the solution was diluted with DCM, washed with aqueous $NaHCO_3$ solution, and concentrated. The crude material was purified via reverse-phase automated flash column chromatography (Teledyne ISCO, 15.5 g HPC18 RediSep Rf Gold Column, liquid loading with DMSO, 50-95% MeCN in 0.5 mL/L aq NH_4OH) to afford the title compound as a yellow oil (8.5 mg, 24% yield). NMR spectra matched the original synthesis/natural isolates of **4**.[2]

In a similar fashion, 10.8 mg of **18** was subjected to the reaction conditions with the following modifications: reaction was run with 0.2 eq DMAP with a reaction concentration of 0.02 M to give 1.1 mg **4** in 8% yield (yellow oil) after purification by column chromatography (Teledyne ISCO, HPC18 RediSep Rf Gold Column, 50-95% MeCN in 0.5 mL/L aq NH_4OH).

References

1. Das, J.; Vellakkaran, M.; Banerjee, D. Nickel-Catalyzed Alkylation of Ketone Enolates: Synthesis of Monoselective Linear Ketones. *J. Org. Chem*. **2019**, *84*, 769-779.

2. Liu, H.; Wang, X.; Shi, Q.; Li, L.; Zhang, Q.; Wu, Z.-L.; Huang, X.-J.; Zhang, Q.-W.; Ye, W.-C.; Wang, Y.; Shi, L. Dimeric Diarylheptanoids with Neuroprotective Activities from Rhizomes of *Alpinia officinarum*. *ACS Omega* **2020**, *5*, 10167-10175.

Synthesis of Natural Product Officinine B (3) (Route 1)

4-((*Tert*-butyldimethylsilyl)oxy)-3-methoxybenzaldehyde (21)

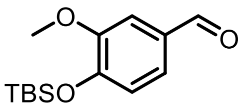

To a vial was added vanillin (1.00 g, 6.59 mmol, 1.0 eq), imidazole (901 mg, 13.2 mmol, 2.0 eq), and 4-dimethylaminopyridine (9.8 mg, 80.2 µmol, 0.01 eq), which were dissolved in anhydrous DCM (6.5 mL). The solution was cooled to 0 °C. *Tert*-butyldimethylsilyl chloride (1.46 g, 9.69 mmol, 1.5 eq) was added, and the solution was stirred for 4 h while being allowed to slowly warmed to r.t. The reaction was quenched with water and extracted several times with DCM. The organic layers were combined and concentrated, then the crude material was purified via automated flash column chromatography (Teledyne ISCO, 40g column, 0-10% EtOAc in hexanes) to afford the title compound as a yellow oil (1.40 g, 5.27 mmol, 80% yield).
^{1}H NMR (400 MHz, $CDCl_3$) δ 9.85 (s, 1H), 7.40 (d, *J* = 1.9 Hz, 1H), 7.37 (dd, *J* = 8.0, 1.9 Hz, 1H), 6.96 (d, *J* = 8.0 Hz, 1H), 3.87 (s, 3H), 1.00 (s, 9H), 0.19 (s, 6H).
^{13}C NMR (101 MHz, $CDCl_3$) δ 191.2, 151.8, 151.5, 131.1, 126.4, 120.9, 110.2, 55.6, 25.7, 18.7, -4.4.
HRMS (TOF-ESI) $[M+H]^+$ calcd for $C_{14}H_{23}O_3Si$ 267.1411; found 267.1408.
Note: NMR data align with literature.[1]

(4-((*Tert*-butyldimethylsilyl)oxy)-3-methoxyphenyl)methanol (22)

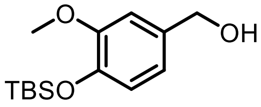

A solution of 4-((*tert*-butyldimethylsilyl)oxy)-3-methoxybenzaldehyde (1.41 g, 5.28 mmol, 1.0 eq) in anhydrous methanol (21 mL) was added to a 100 mL round bottom flask. The solution was cooled to 0 °C, then sodium borohydride (309 mg, 8.15 mmol, 1.5 eq) was added in three portions over the course of 10 min. The mixture was stirred for 2 h while being allowed to slowly warm to r.t., then the reaction was quenched with 25 mL water, extracted with 3x25 mL portions of DCM, filtered over anhydrous $MgSO_4$, and concentrated to afford the title compound as a yellow oil (1.06 g, 3.94 mmol, 75% yield).
^{1}H NMR (400 MHz, $CDCl_3$) δ 6.90 (d, *J* = 1.6 Hz, 1H), 6.83 (d, *J* = 8.0 Hz, 1H), 6.79 (dd, *J* = 8.0, 1.8 Hz, 1H), 4.61 (d, *J* = 3.6 Hz, 2H), 3.82 (s, 3H), 1.56 (t, *J* = 4.9 Hz, 1H, partially buried under water signal), 0.99 (s, 9H), 0.15 (s, 6H).
^{13}C NMR (101 MHz, $CDCl_3$) δ 151.2, 144.8, 134.6, 121.0, 119.7, 111.3, 65.6, 55.6, 25.9, 18.6, -4.5.
HRMS (TOF-ESI) $[M-OH]^+$ calcd for $C_{14}H_{23}O_2Si$ 251.1462; found 251.1461.
Note: NMR data align with literature.[2]

***Tert*-butyl(4-(chloromethyl)-2-methoxyphenoxy)dimethylsilane (23)**

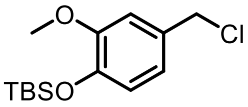

A heat gun-dried 100 mL round bottom flask was charged with a solution of (4-((*tert*-butyldimethylsilyl)oxy)-3-methoxyphenyl)methanol (712 mg, 2.65 mmol, 1.0 eq) in anhydrous DCM (17 mL) under N_2, then was cooled to 0 °C. Thionyl chloride (580 µL, 7.95 mmol, 3.0 eq) was added dropwise, and the solution was stirred for 3.6 h (temperature maintained under 10 °C). The solution was concentrated to afford the title compound as a red-gold oil in quantitative yield, which was taken directly to the next step without purification.
^{1}H NMR (400 MHz, $CDCl_3$) δ 6.88 (d, *J* = 1.9 Hz, 1H), 6.83 (dd, *J* = 8.1, 2.0 Hz, 1H), 6.80 (d, *J* = 8.0 Hz, 1H), 4.55 (s, 2H), 3.82 (s, 3H), 0.99 (s, 9H), 0.15 (s, 6H).
^{13}C NMR (101 MHz, $CDCl_3$) δ 151.2, 145.5, 131.0, 121.4, 121.0, 112.7, 55.6, 46.9, 25.8, 18.6, -4.5.

***Tert*-butyl(2-methoxy-4-(3-(trimethylsilyl)prop-2-yn-1-yl)phenoxy)dimethylsilane (25)**

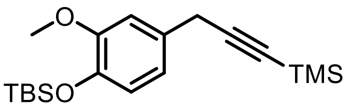

To an oven-dried 50 mL round bottom flask under N_2 was added ethynyltrimethylsilane (560 µL, 4.04 mmol, 4.5 eq) and anhydrous THF (8 mL). The solution was cooled to 0 °C, then isopropylmagnesium chloride (2.0 mL, 4.00 mmol, 4.4 eq) was added dropwise. The solution was stirred at this temperature for 0.5 h, then was warmed to r.t. and stirred for 1 h. At this time, CuBr (77.9 mg, 0.54 mmol, 0.6 eq) was added, and the mixture was stirred at r.t. for 30 min. At this time, a solution of *tert*-butyl(4-(chloromethyl)-2-methoxyphenoxy)dimethylsilane (259 mg, 0.90 mmol, 1.0 eq) in anhydrous THF (0.9 mL) was added dropwise, and the mixture was heated to 66 °C overnight. After 19 h, the reaction was cooled to r.t., quenched with 15 mL aqueous NH_4Cl, extracted with 3x15 mL portions of DCM, and concentrated. The crude material was purified via automated flash column chromatography (Teledyne ISCO, 12g column, 0-10% EtOAc in hexanes) to afford the title compound as a red-gold oil (237 mg, 0.679 mmol, 75% yield).
^{1}H NMR (400 MHz, $CDCl_3$) δ 6.88 (d, *J* = 1.4 Hz, 1H), 6.79 (d, *J* = 8.0 Hz, 1H), 6.77 – 6.74 (m, 1H), 3.80 (s, 3H), 3.59 (s, 2H), 0.99 (s, 9H), 0.19 (s, 9H), 0.15 (s, 6H).
^{13}C NMR (101 MHz, $CDCl_3$) δ 151.0, 143.7, 129.7, 120.9, 120.1, 112.0, 104.9, 87.0, 55.5, 25.9, 25.9, 18.6, 0.3, -4.5.
HRMS (TOF-ESI) – no observed ionization.

2-Methoxy-4-(prop-2-yn-1-yl)phenol (26)

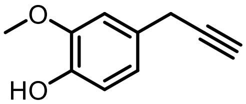

To a vial containing *tert*-butyl(2-methoxy-4-(3-(trimethylsilyl)prop-2-yn-1-yl)phenoxy)dimethylsilane (232 mg, 0.66 mmol, 1.0 eq) was added anhydrous MeOH (6.5 mL), followed by K_2CO_3 (556 mg, 4.02 mmol, 6.0 eq). The mixture was stirred at r.t. for 3.3 h, then was quenched with DOWEX 50WX-200 ion exchange resin, extracted three times with DCM, and

concentrated. The crude material was purified via automated flash column chromatography (Teledyne ISCO, 40g column, 0-25% EtOAc in hexanes) to afford the title compound as a light yellow oil (88.0 mg, 0.543 mmol, 82% yield).
^{1}H NMR (400 MHz, $CDCl_3$) δ 6.89 – 6.80 (m, 3H), 5.51 (s, 1H), 3.90 (s, 3H), 3.56 – 3.52 (m, 2H), 2.18 (t, J = 2.7 Hz, 1H).
^{13}C NMR (101 MHz, $CDCl_3$) δ 146.7, 144.6, 128.0, 120.7, 114.5, 110.6, 82.5, 70.4, 56.1, 24.6.
HRMS (TOF-ESI) $[M\text{-}H^-]^+$ calcd for $C_{10}H_9O_2$ 161.0597; found 161.0595.
Note: Terminal alkynes typically exhibit an $[M\text{-}1]^+$ major ion peak, as is seen here.[3]

(*E*)-2-Methoxy-4-(3-(4,4,5,5-tetramethyl-1,3,2-dioxaborolan-2-yl)allyl)phenol (27)

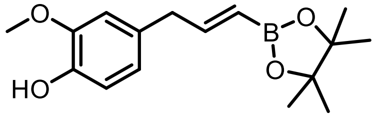

To a dry vial under N_2 was added CuCl (11.5 mg, 0.12 mmol, 0.2 eq), B_2pin_2 (151 mg, 0.59 mmol, 1.1 eq), NaOtBu (10.3 mg, 0.11 mmol, 0.2 eq), and bis[(2-diphenylphosphino)phenyl] ether (38.3 mg, 71 µmol, 0.1 eq), followed by anhydrous THF (4.4 mL). The mixture was stirred at r.t. for 11 min, then a solution of 2-methoxy-4-(prop-2-yn-1-yl)phenol (88.1 mg, 0.54 mmol, 1.0 eq) and anhydrous MeOH (44 µL, 1.09 mmol, 2.0 eq) in anhydrous THF (1.0 mL) was added. The vial headspace was evacuated and filled 3x with N_2, then the mixture was stirred at r.t. for 17 h. The reaction was then diluted with DCM, filtered through PTFE, concentrated, and purified via automated flash column chromatography (Teledyne ISCO, 12g column, 0-100% DCM in hexanes) to afford the title compound as a white solid (27.0 mg, 93 µmol, 17% yield; 32% brsm).
^{1}H NMR (400 MHz, $CDCl_3$) δ 6.85 – 6.81 (m, 1H), 6.74 (dt, J = 17.8, 6.3 Hz, 1H), 6.68 – 6.64 (m, 2H), 5.53 (s, 1H), 5.43 (dt, J = 17.8, 1.6 Hz, 1H), 3.85 (s, 3H), 3.40 (dd, J = 6.3, 1.4 Hz, 2H), 1.25 (s, 12H).
^{13}C NMR (101 MHz, $CDCl_3$) δ 153.0, 146.6, 144.1, 131.0, 121.7, 119.5, 114.4, 111.6, 83.3, 56.0, 42.1, 24.9.
HRMS (TOF-ESI) $[M+H]^+$ calcd for $C_{16}H_{24}BO_4$ 291.1765; found 291.1767.
Melting point: 97 – 99 °C
Note: NMR characterization data aligns with literature, though previously the *E* isomer was not separated from its *Z* counterpart.[4]

Officinine B (3)

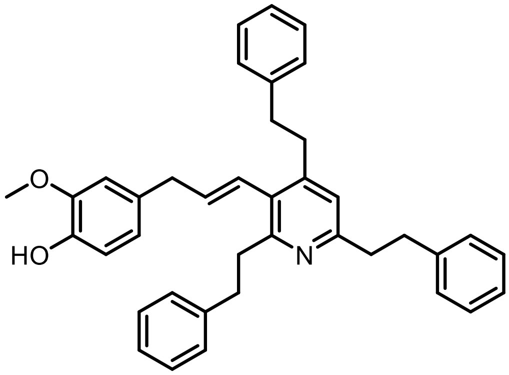

To a vial was added 3-bromo-2,4,6-triphenethylpyridine (20.2 mg, 43 µmol, 1.0 eq), Cs_2CO_3 (63.2 mg, 0.19 mmol, 4.5 eq), and Pd(dppf)Cl_2 (3.9 mg, 5.3 µmol, 0.1 eq), followed by a solution of (*E*)-2-methoxy-4-(3-(4,4,5,5-tetramethyl-1,3,2-dioxaborolan-2-yl)allyl)phenol (27.0 mg,

93 µmol, 2.2 eq) in 1,4-dioxane (0.9 mL), then water (0.15 mL). The mixture was degassed with N_2 for 5 min, then was sealed and heated to 100 °C for 23 h. The reaction was then cooled to r.t., diluted with water, neutralized with aqueous NH_4Cl, extracted multiple times with DCM and 3:1 chloroform:IPA solution, and concentrated. The crude material was purified via automated flash column chromatography (Teledyne ISCO, 12g column, 0-100% DCM in hexanes, then 0-15% EtOAc in DCM) to afford the title compound as a yellow oil (20.0 mg, 36 µmol, 84% yield).
^{1}H NMR (400 MHz, $CDCl_3$) δ 7.33 – 7.15 (m, 11H), 7.13 – 7.08 (m, 2H), 7.04 – 7.00 (m, 2H), 6.85 (d, *J* = 8.0 Hz, 1H), 6.73 (dd, *J* = 8.0, 1.9 Hz, 1H), 6.73 (s, 1H), 6.69 (d, *J* = 1.8 Hz, 1H), 6.22 (dt, *J* = 16.0, 1.2 Hz, 1H), 5.71 (dt, *J* = 16.0, 6.7 Hz, 1H), 5.70 (br s, 1H), 3.70 (s, 3H), 3.47 (dd, *J* = 6.7, 1.0 Hz, 2H), 3.15 – 3.08 (m, 2H), 3.08 – 3.01 (m, 4H), 3.01 – 2.94 (m, 2H), 2.85 – 2.77 (m, 2H), 2.76 – 2.68 (m, 2H).
^{13}C NMR (101 MHz, $CDCl_3$) δ 158.6, 158.5, 149.0, 146.8, 144.3, 142.2, 141.9, 141.4, 136.0, 131.7, 130.2, 128.7, 128.6, 128.54, 128.45, 128.42, 128.37, 126.2, 126.0, 125.91, 125.87, 121.44, 121.2, 114.6, 111.2, 55.8, 39.8, 39.7, 38.1, 36.6, 36.3, 36.0, 35.4.n
HRMS (TOF-ESI) $[M+H]^+$ calcd for $C_{39}H_{40}NO_2$ 554.3054; found 554.3056.

All data were in excellent agreement with the natural isolate.[5]

References

1. Shirai, T.; Kumihashi, K.; Sakasai, M.; Kusuoku, H.; Shibuya, Y.; Ohuchi, A. Identification of a Novel TRPM8 Agonist from Nutmeg: A Promising Cooling Compound. *ACS Med. Chem. Lett.* **2017**, *8*, 715–719.

2. Jadhav, A. H.; Kim, H. A Mild, Efficient, and Selective Deprotection of Tert-Butyldimethylsilyl (TBDMS) Ethers Using Dicationic Ionic Liquid as a Catalyst. *Tetrahedron Lett.* **2012**, *53*, 5338–5342.

3. *Structure Determination of Organic Compounds: Tables of Spectral Data*; Springer Berlin Heidelberg: Berlin, Heidelberg, 2009. https://doi.org/10.1007/978-3-540-93810-1.

4. Hemelaere, R.; Carreaux, F.; Carboni, B. Synthesis of Alkenyl Boronates from Allyl-Substituted Aromatics Using an Olefin Cross-Metathesis Protocol. *J. Org. Chem.* **2013**, *78*, 6786–6792.

5. Zhao, L.; Liang, J. Y.; Qu, W. A Novel Dimeric Diarylheptanoid from the Rhizomes of Alpinia Officinarum. *Chem. Nat. Compd.* **2012**, *48*, 836–838.

Synthesis of Natural Product Officinine B (3) (Route 2)

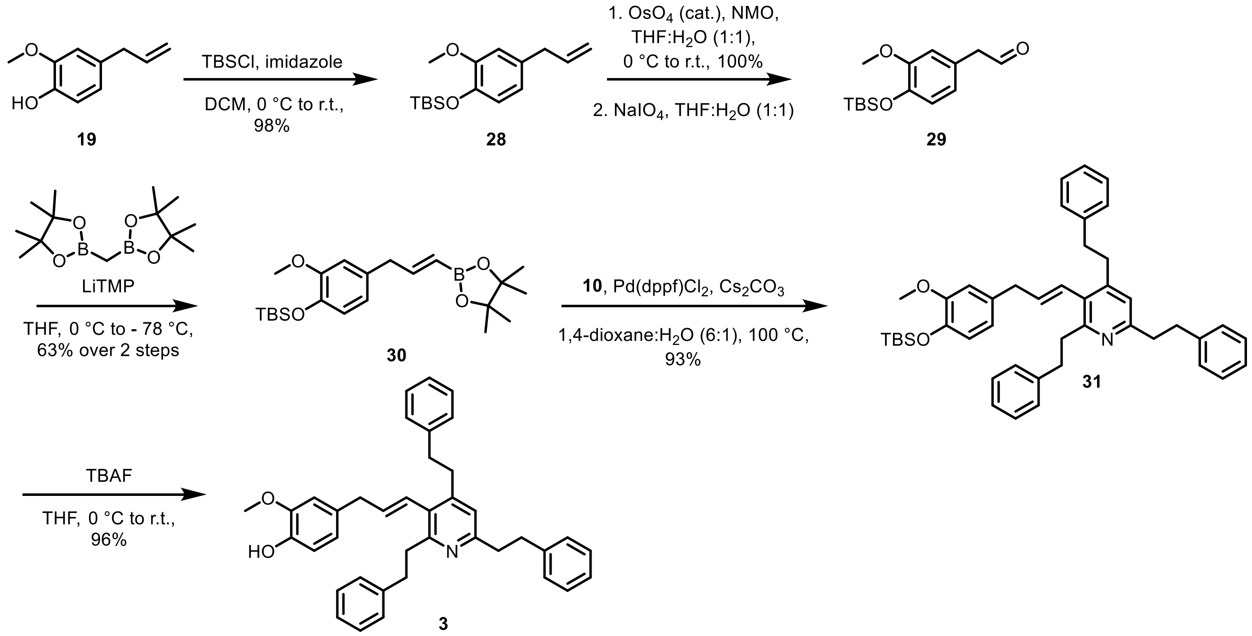

(4-Allyl-2-methoxyphenoxy)(*tert*-butyl)dimethylsilane (28)

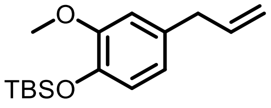

To a solution of eugenol (500 mg, 0.467 mL, 3.05 mmol, 1.0 eq) in anhydrous DCM (3 mL) was added imidazole (314 mg, 4.62 mmol, 1.5 eq) and 4-dimethylaminopyridine (4.0 mg, 0.03 mmol, 0.01 eq) under an N_2 atmosphere. The solution was cooled to 0 °C, using a water-ice bath and *tert*-butyldimethylsilyl chloride (TBSCl, 555 mg, 3.68 mmol, 1.2 eq) was added and the resulting mixture was stirred at r.t. for 5 h. The reaction was diluted with water (3 mL), the organic phase was separated, and the aqueous layer was extracted with DCM (3x5 mL). The combined organic layers were concentrated under vacuum. The resulting crude residue was purified by flash column chromatography (Teledyne ISCO solid loading, 0-70% EtOAc in hexanes) to afford the title compound as a colorless oil (832 mg, 2.99 mmol, 98% yield).

^{1}H NMR (400 MHz, $CDCl_3$) δ 6.77 (d, *J* = 7.9 Hz, 1H), 6.68 (d, *J* = 2.1 Hz, 1H), 6.63 (dd, *J* = 8.0, 2.1 Hz, 1H), 5.97 (ddt, *J* = 16.8, 10.2, 6.7 Hz, 1H), 5.12 – 5.01 (m, 2H), 3.79 (s, 3H), 3.33 (d, *J* = 6.7 Hz, 2H), 1.00 (s, 9H), 0.15 (s, 6H).

^{13}C NMR (101 MHz, $CDCl_3$) δ 150.9, 143.4, 138.0, 133.6, 120.9, 120.8, 115.6, 112.7, 55.6, 40.0, 25.9, 18.6, -4.5.

HRMS (TOF-ESI) $[M+H]^+$ calcd for $C_{16}H_{27}O_2Si$ 279.1775; found 279.1769.

2-(4-((*Tert*-butyldimethylsilyl)oxy)-3-methoxyphenyl)acetaldehyde (29)

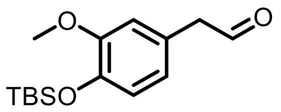

To a solution of (4-allyl-2-methoxyphenoxy)(*tert*-butyl)dimethylsilane (134 mg, 0.48 mmol, 1.0 eq) in THF (2.3 mL) and water (2.3 mL) was added osmium tetroxide (2.5% wt in *tert*-butanol, 489 mg, 0.6 mL, 0.05 mmol, 0.1 eq) and 4-methylmorpholine *N*-oxide (67.6 mg, 0.58 mmol, 1.2 eq) at 0 °C. The resulting mixture was allowed to warm to r.t. and stirred for 1 h. Upon completion of the dihydroxylation, the reaction was quenched with sat. aqueous $Na_2S_2O_3$ and diluted with DCM (10 mL). The organic phase was separated, and the aqueous layer was extracted with DCM (3x10 mL) followed by 3:1 chloroform/IPA solution (10 mL). The combined organic layers were concentrated under vacuum. The resulting crude residue was purified by flash column chromatography (Teledyne ISCO solid loading, 0-100% EtOAc in hexanes) to afford the dihydroxylation product as a white solid (150.3 mg, 0.48 mmol, 100% yield).
^{1}H NMR (400 MHz, $CDCl_3$) δ 6.78 (d, *J* = 7.9 Hz, 1H), 6.70 (d, *J* = 2.1 Hz, 1H), 6.65 (dd, *J* = 8.0, 2.1 Hz, 1H), 3.96 – 3.85 (m, 1H), 3.79 (s, 3H), 3.67 (dd, *J* = 11.2, 3.2 Hz, 1H), 3.50 (dd, *J* = 11.2, 6.9 Hz, 1H), 2.77 – 2.61 (m, 2H), 2.13 (s, 2H), 0.99 (s, 9H), 0.14 (s, 6H).
^{13}C NMR (101 MHz, $CDCl_3$) δ 151.1, 143.9, 131.1, 121.6, 121.1, 113.3, 73.2, 66.2, 55.6, 39.6, 25.8, 18.6, -4.5;
HRMS (TOF-ESI) $[M+H]^+$ calcd for $C_{16}H_{29}O_4Si$ 313.1830; found 313.1821.
Melting point: 66 – 67 °C

To a solution of 3-(4-((*tert*-butyldimethylsilyl)oxy)-3-methoxyphenyl)propane-1,2-diol (120 mg, 0.38 mmol, 1.0 eq) in THF (1.6 mL) and water (1.6 mL) was added sodium periodate (124 mg, 0.58 mmol, 1.5 eq) in one portion. The resulting mixture was stirred at r.t. for 1 h. Upon complete consumption of the starting material, the reaction was diluted with water (10 ml) and DCM (10 mL). The organic phase was separated, and the aqueous layer was extracted with DCM (3x10 mL). The combined organic layer was washed with water (~20 ml) to ensure the complete removal of any remaining periodate and concentrated under vacuum (while maintaining the water bath temperature of the rotary evaporator at 30 °C until it reached ~10% of the original volume). The remaining solvent was removed using a stream of nitrogen to afford the title compound (108 mg) as a colorless oil. This was immediately taken to the next step without further purification or stored in dry ice (-78.5 °C) until used. Quantitative yield was assumed.
The product was confirmed by low resolution mass spectrometry: LRMS (SQ MS with ESI) $[M+H]^+$ calcd for $C_{15}H_{25}O_3Si$ 281.1; found 281.2.
Note: Purification of the 1,2-diol prior to oxidative cleavage with sodium periodate is important. Once the oxidative cleavage was performed on the crude, complete degradation of the desired aldehyde to the one carbon lower homolog aldehyde was observed. This phenomenon was previously observed and reported by Cossy *et al.*[1]

(*E*)-*Tert*-butyl(2-methoxy-4-(3-(4,4,5,5-tetramethyl-1,3,2-dioxaborolan-2-yl)allyl)phenoxy)dimethylsilane (30)

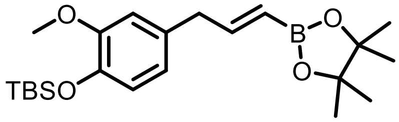

The boron−Wittig reaction was performed following a modified reported procedure.[2] An oven dried vial, charged with freshly prepared LiTMP[3] (68.0 mg, 0.46 mmol, 1.2 eq) under N_2 atmosphere was cooled to 0 °C using a water-ice bath. Anhydrous THF (0.5 mL) was added to the reaction followed by gentle stirring. A solution of bis[(pinacolato)boryl]methane (124 mg, 0.46 mmol, 1.2 eq) in anhydrous THF (1.0 mL) was added dropwise and stirred at 0 °C for 5 min. Then the reaction was cooled to -78 °C using a dry ice-acetone bath and a solution of 2-(4-((*tert*-butyldimethylsilyl)oxy)-3-methoxyphenyl)acetaldehyde (108 mg, 0.38 mmol, 1.0 eq) in anhydrous THF (0.5 mL) was added dropwise and allowed to stir at -78 °C for 4 h. Once the aldehyde was completely consumed, the reaction was allowed to warm to r.t. and concentrated under vacuum. The resulting crude residue was purified by flash column chromatography (Teledyne ISCO solid loading, 0-100% DCM in hexanes) to afford the title compound as a clear oil (98.0 mg, 0.24 mmol, 63% yield over 2 steps).
^{1}H NMR (400 MHz, $CDCl_3$) δ 6.80 – 6.69 (m, 2H), 6.64 (d, *J* = 2.1 Hz, 1H), 6.61 (dd, *J* = 8.0, 2.1 Hz, 1H), 5.44 (dt, *J* = 17.8, 1.7 Hz, 1H), 3.77 (s, 3H), 3.40 (dd, *J* = 6.3, 1.8 Hz, 2H), 1.26 (s, 12H), 0.99 (s, 9H), 0.14 (s, 6H).
^{13}C NMR (101 MHz, $CDCl_3$) δ 153.1, 150.9, 143.5, 132.6, 121.2, 120.9, 119.5, 113.1, 83.2, 55.6, 42.2, 25.9, 24.9, 18.6, -4.5.
HRMS (TOF-ESI) $[M+H]^+$ calcd for $C_{22}H_{38}BO_4Si$ 405.2631; found 405.2636.

(*E*)-3-(3-(4-((*Tert*-butyldimethylsilyl)oxy)-3-methoxyphenyl)prop-1-en-1-yl)-2,4,6-triphenethylpyridine (31)

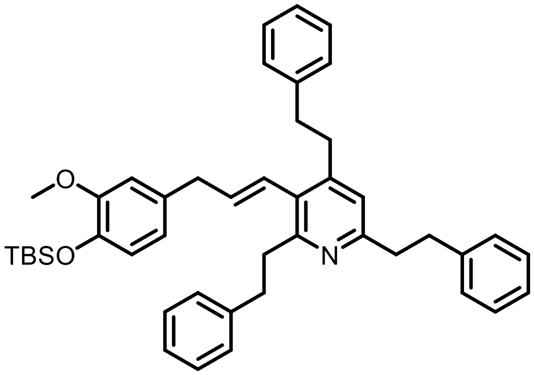

To a solution of 3-bromo-2,4,6-triphenethylpyridine (25.0 mg, 0.05 mmol, 1.0 eq) in 1,4-dioxane (0.455 mL) and water (0.075 mL) was added (*E*)-*tert*-butyl(2-methoxy-4-(3-(4,4,5,5-tetramethyl-1,3,2-dioxaborolan-2-yl)allyl)phenoxy)dimethylsilane (43.0 mg, 0.11 mmol, 2.0 eq), cesium carbonate (52.3 mg, 0.16 mmol, 3.0 eq), and Pd(dppf)Cl_2 (7.8 mg, 0.01 mmol, 0.2 eq) under an N_2 atmosphere. The resulting solution was stirred at 100 °C until the starting material was completely consumed (~ 6 h). Upon completion, the reaction mixture was diluted with DCM (5 mL) and sat. aqueous $NaHCO_3$ (2 mL). The organic layer was separated, and the aqueous layer was extracted with DCM (3x5 mL). The combined organic layers were concentrated under vacuum. The resulting crude residue was purified by flash column chromatography (Teledyne ISCO solid loading, 0-100% EtOAc in hexanes) to afford the title compound as a clear oil (33.0 mg, 0.05 mmol, 93% yield).
^{1}H NMR (400 MHz, $CDCl_3$) δ 7.32 – 7.16 (m, 11H), 7.12 (d, *J* = 6.8 Hz, 2H), 7.03 (d, *J* = 6.8 Hz, 2H), 6.77 – 6.62 (m, 4H), 6.20 (d, *J* = 16.0 Hz, 1H), 5.68 (dt, *J* = 16.1, 6.7 Hz, 1H), 3.65 (s, 3H), 3.45 (d, *J* = 6.6 Hz, 2H), 3.11 (dd, *J* = 9.9, 5.6 Hz, 2H), 3.06 – 2.93 (m, 6H), 2.85 – 2.77 (m, 2H), 2.76 – 2.67 (m, 2H), 1.00 (s, 9H), 0.14 (s, 6H).

^{13}C NMR (101 MHz, CDCl$_3$) δ 158.7, 158.5, 151.1, 148.8, 143.6, 142.3, 142.0, 141.4, 135.9, 133.2, 130.2, 128.7, 128.7, 128.5, 128.5, 128.4, 128.4, 126.2, 125.98, 125.97, 125.9, 121.1, 121.0, 120.9, 112.7, 55.5, 39.9, 39.6, 38.1, 36.6, 36.3, 35.9, 35.4, 25.9, 18.6, -4.5.
HRMS (TOF-ESI) $[M+H]^+$ calcd for $C_{45}H_{54}NO_2Si$ 668.3918; found 668.3912.

Officinine B (3)

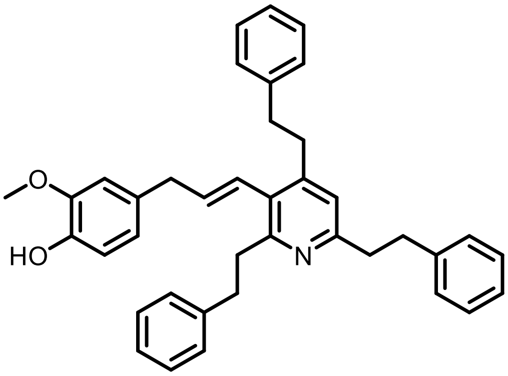

To a solution of (*E*)-3-(3-(4-((*tert*-butyldimethylsilyl)oxy)-3-methoxyphenyl)prop-1-en-1-yl)-2,4,6-triphenethylpyridine (22.0 mg, 0.03 mmol, 1.0 eq) in anhydrous THF (0.25 mL) was added tetrabutylammonium fluoride (1.0 M in THF, 0.05 mL, 0.05 mmol, 1.5 eq) at 0 °C under an N_2 atmosphere. The resulting mixture was allowed to warm to r.t. and stirred for 2 h. Upon completion, the reaction was quenched with sat. aqueous NH_4Cl (~0.050 mL) and diluted with DCM (3 mL). The organic layer was separated, and the aqueous layer was extracted with DCM (3x3 mL). The combined organic layers were concentrated under vacuum. The resulting crude residue was purified by flash column chromatography (Teledyne ISCO solid loading, 0-50% EtOAc in hexanes) to afford the title compound as a yellow oil (17.5 mg, 0.03 mmol, 96% yield).

All data were in excellent agreement with the material isolated from Route 1, and the natural isolate.[4]

References

1. Belotti, D.; Andreatta, G.; Pradaux, F.; BouzBouz, S.; Cossy, J. Degradation of Aldehydes to One Carbon Lower Homologs. *Tetrahedron Lett*. **2003**, *44*, 3613-3615.

2. Coombs, J. R.; Zhang, L.; Morken, J. P., Synthesis of Vinyl Boronates from Aldehydes by a Practical Boron–Wittig Reaction. *Org. Lett.* **2015**, *17*, 1708-1711.

3. Le, T. V.; Daugulis, O., In situ Ortho-Lithiation/Functionalization of Pentafluorosulfanyl Arenes. *Chem. Commun.* **2022**, *58*, 537-540.

4. Zhao, L.; Liang, J. Y.; Qu, W. A Novel Dimeric Diarylheptanoid from the Rhizomes of Alpinia Officinarum. *Chem. Nat. Compd.* **2012**, *48*, 836–838.

Copies of NMR Spectra

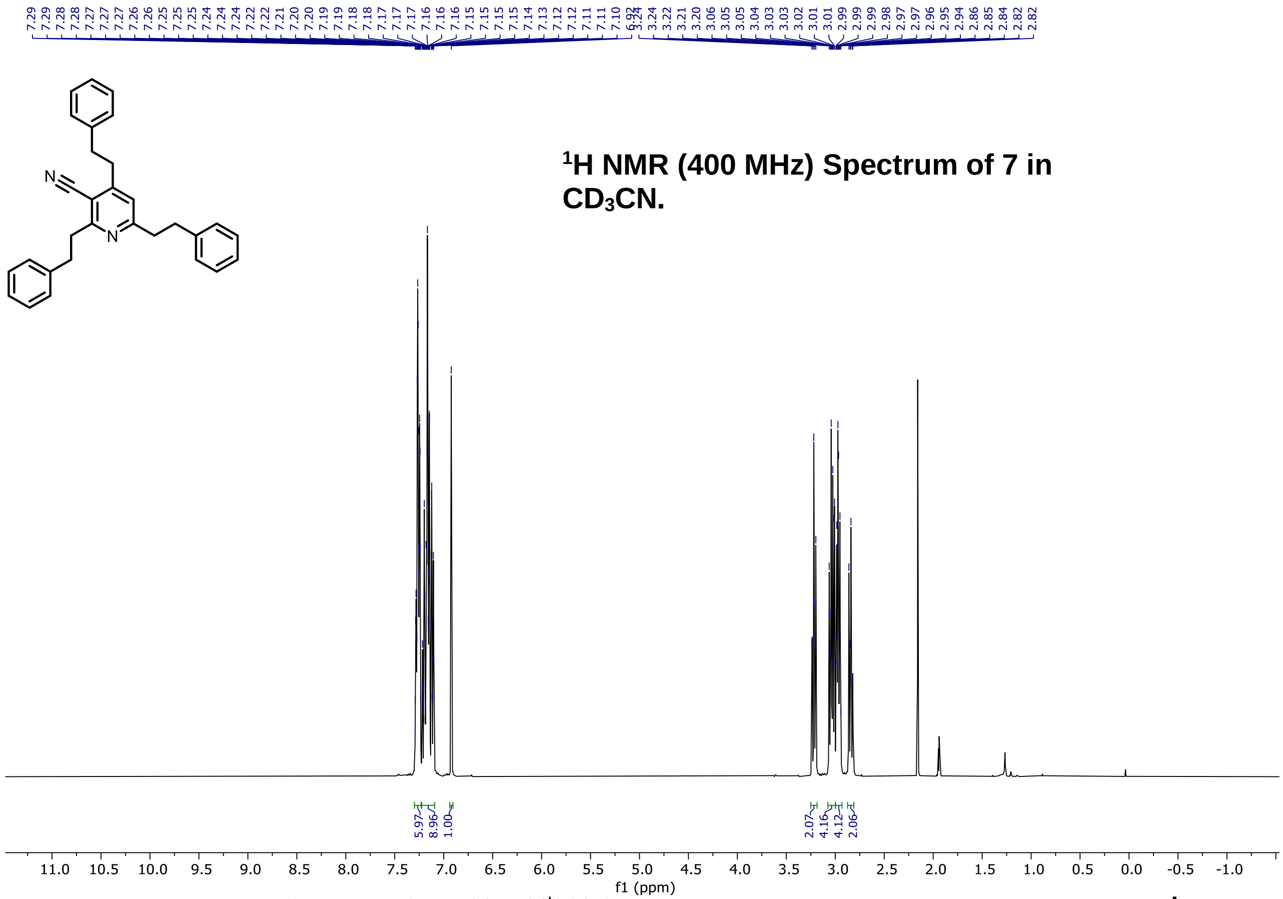

^1H NMR (400 MHz) Spectrum of 7 in CD_3CN.

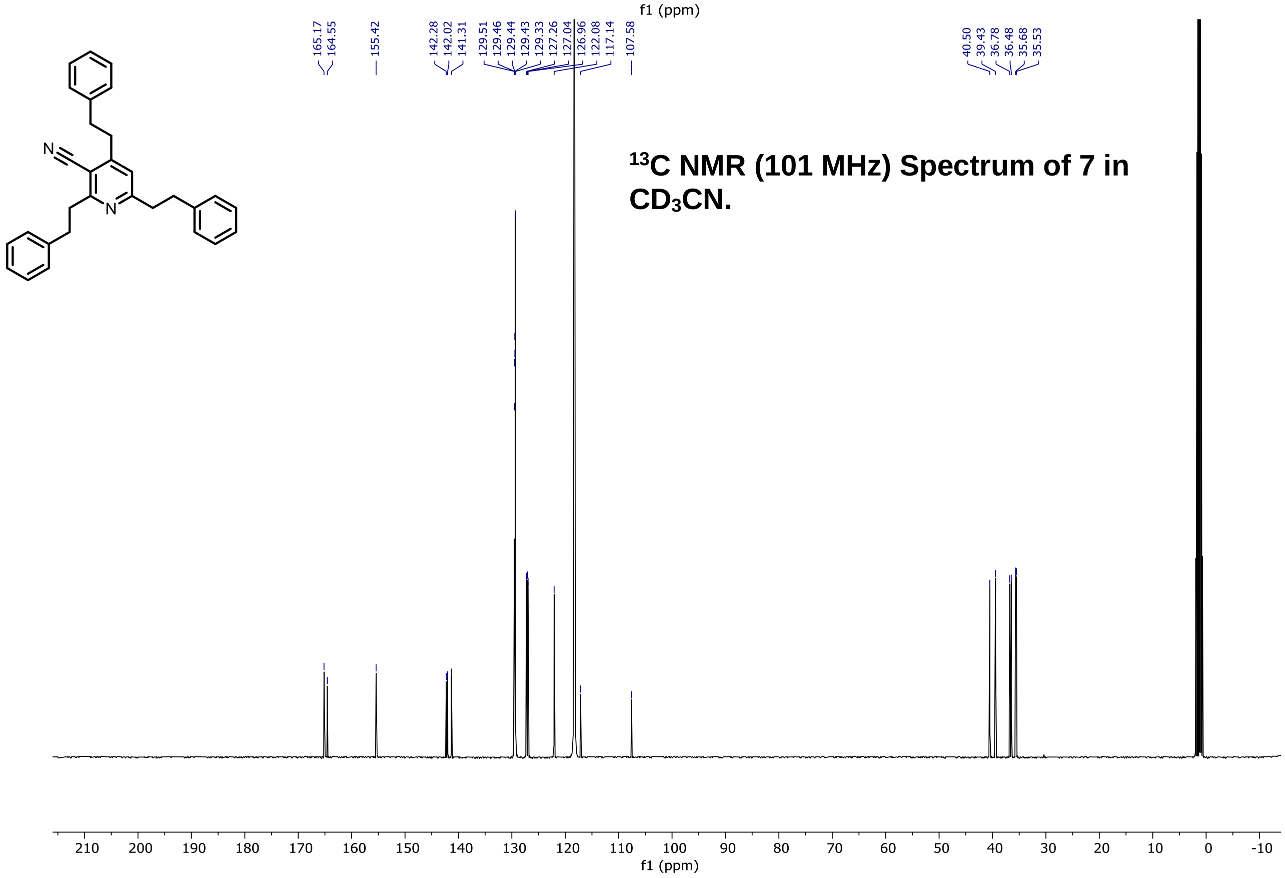

^{13}C NMR (101 MHz) Spectrum of 7 in CD_3CN.

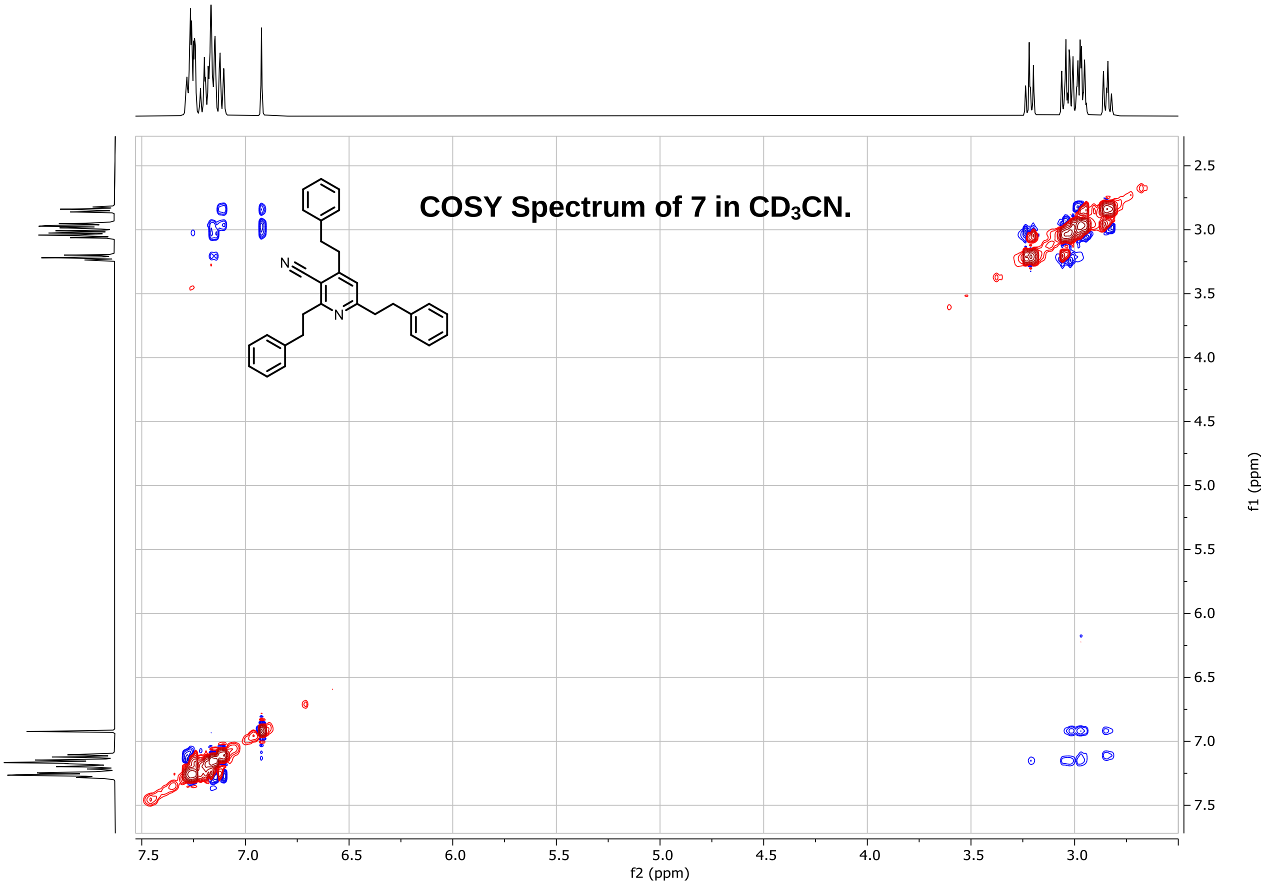
COSY Spectrum of 7 in CD$_3$CN.
N
N
f1 (ppm)
f2 (ppm)

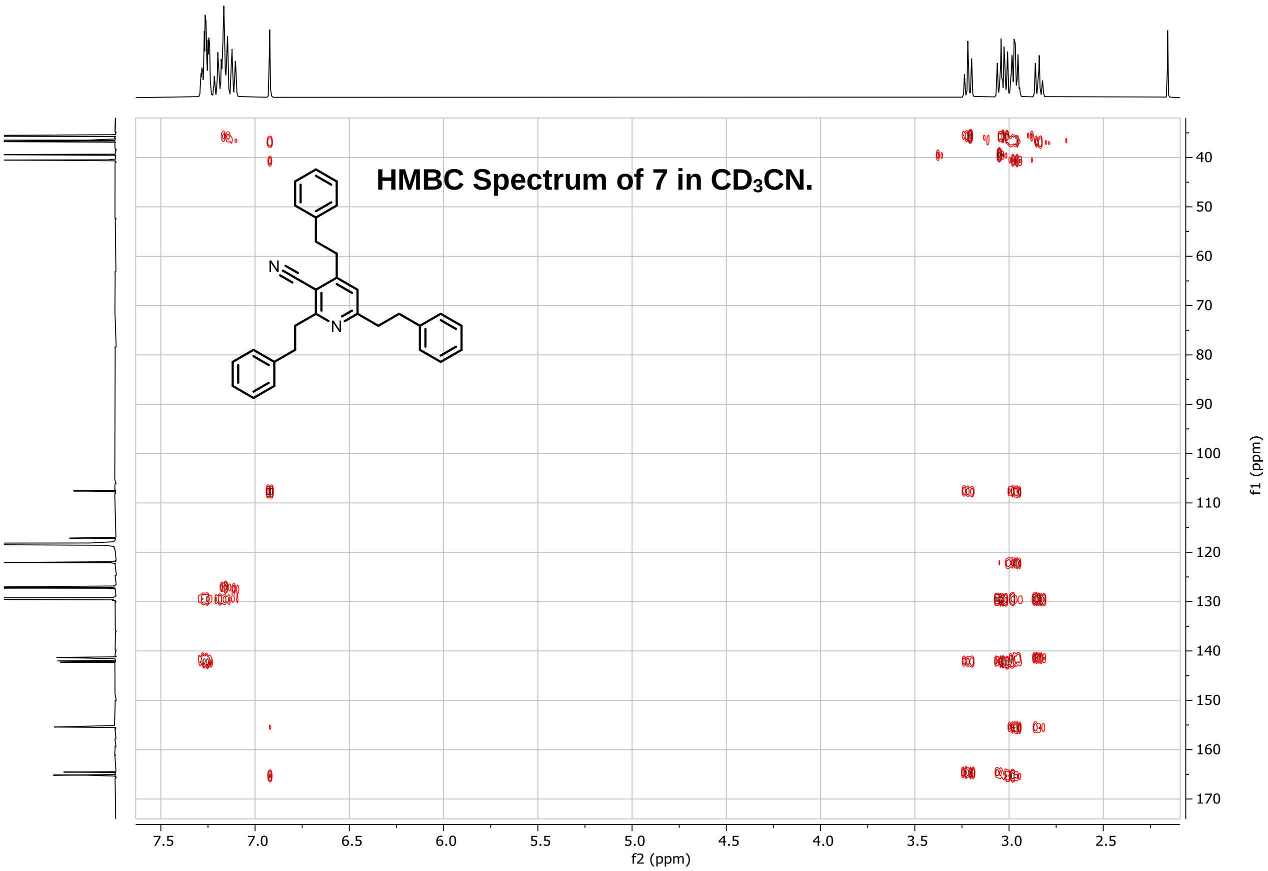
HMBC Spectrum of 7 in CD$_3$CN.
N
N
f1 (ppm)
f2 (ppm)

DEPT-135 Spectrum of 7 in CD_3CN.

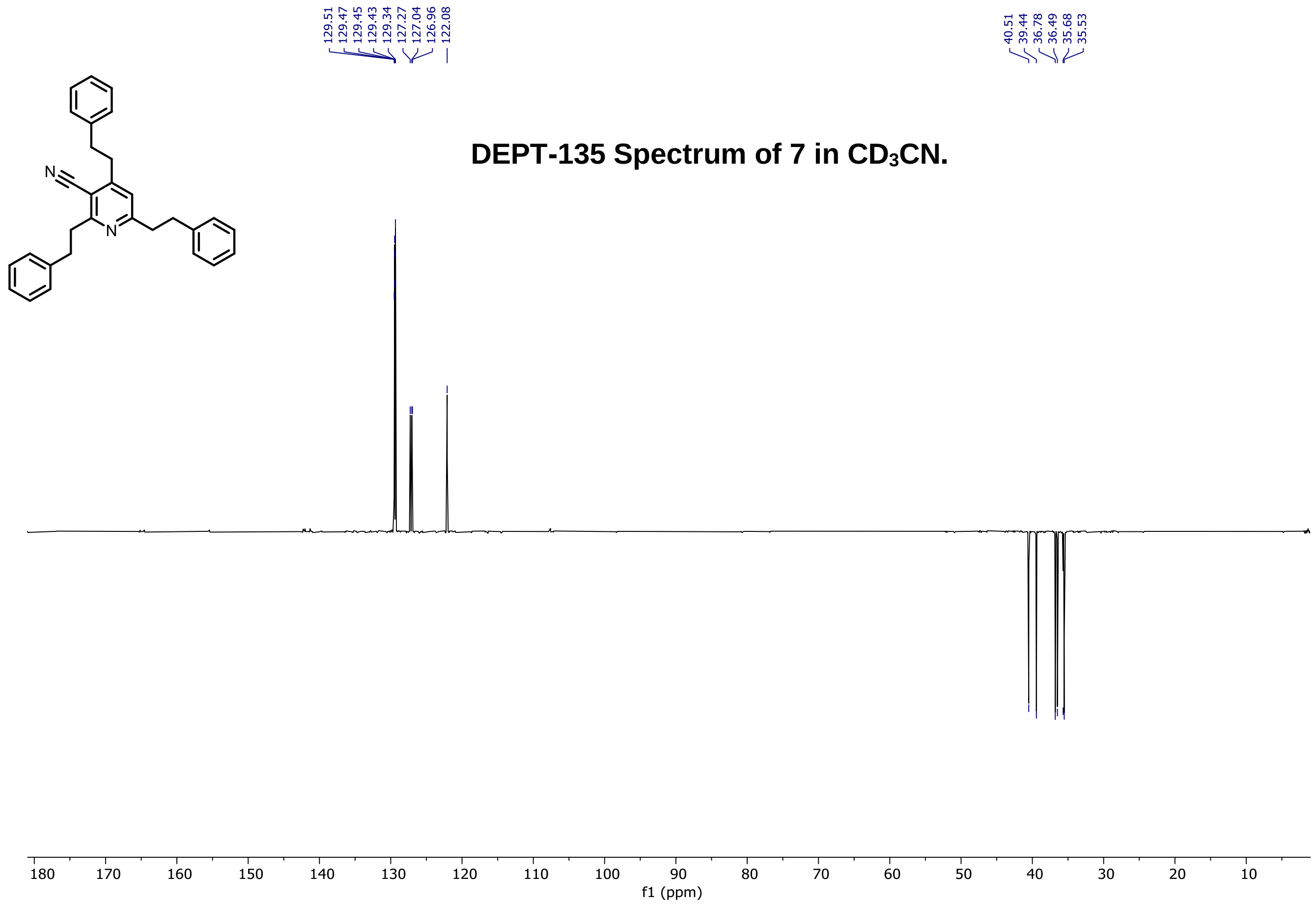

^{1}H NMR (400 MHz) Spectrum of 10 in CD_3CN.

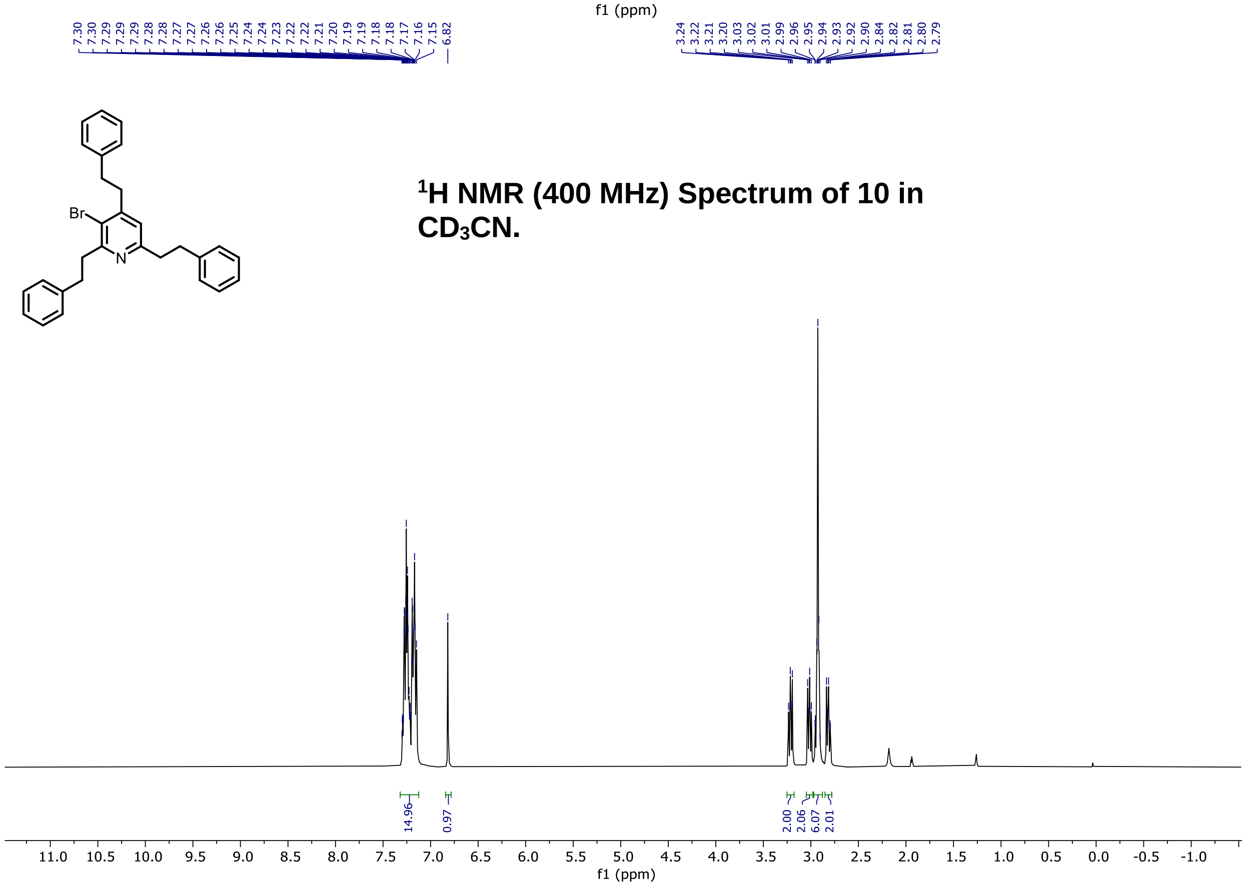

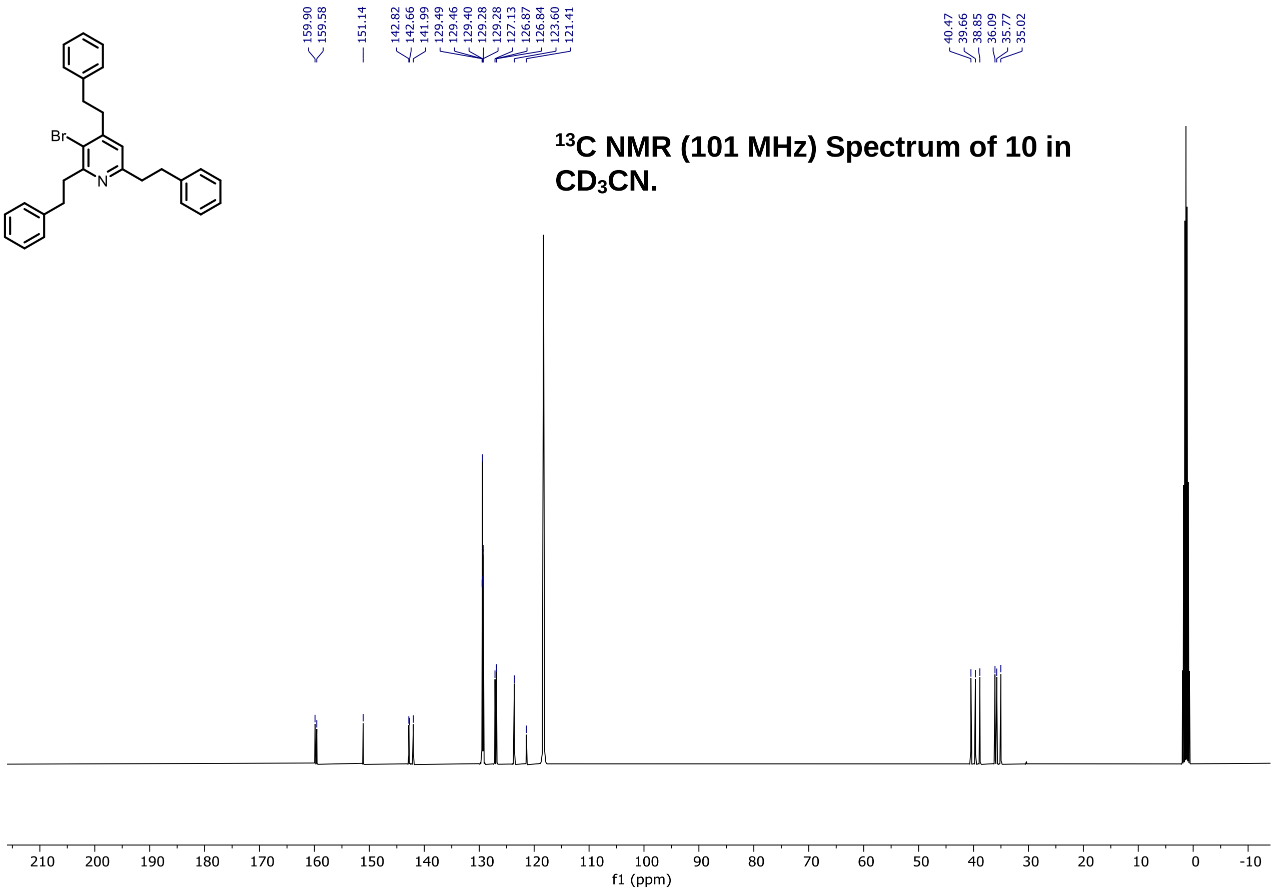
159.90
159.58
151.14
142.82
142.66
141.99
129.49
129.46
129.40
129.28
129.28
127.13
126.87
126.84
123.60
121.41
40.47
39.66
38.85
36.09
35.77
35.02
^{13}C NMR (101 MHz) Spectrum of 10 in CD_3CN.
f1 (ppm)

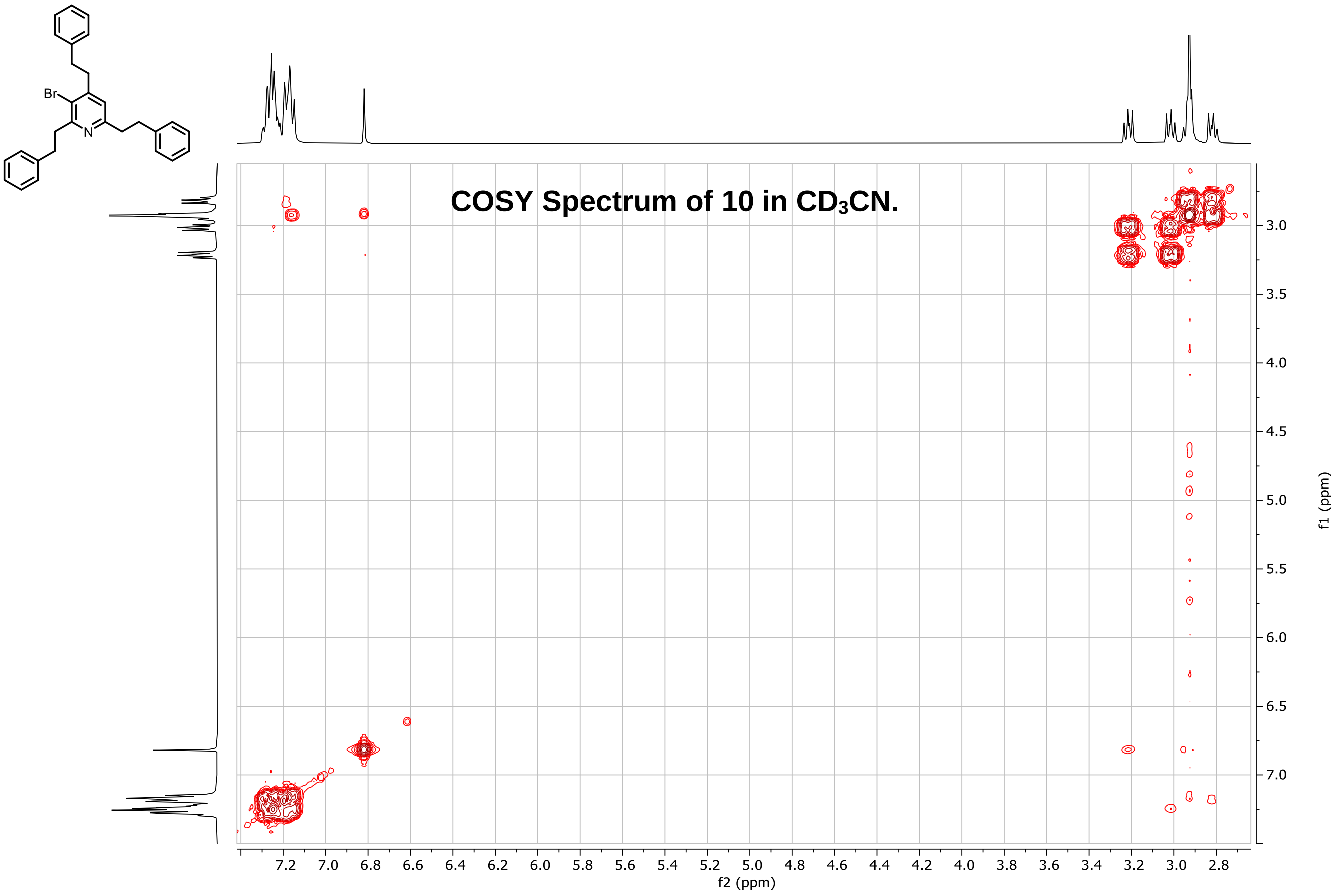
COSY Spectrum of 10 in CD_3CN.
f2 (ppm)
f1 (ppm)

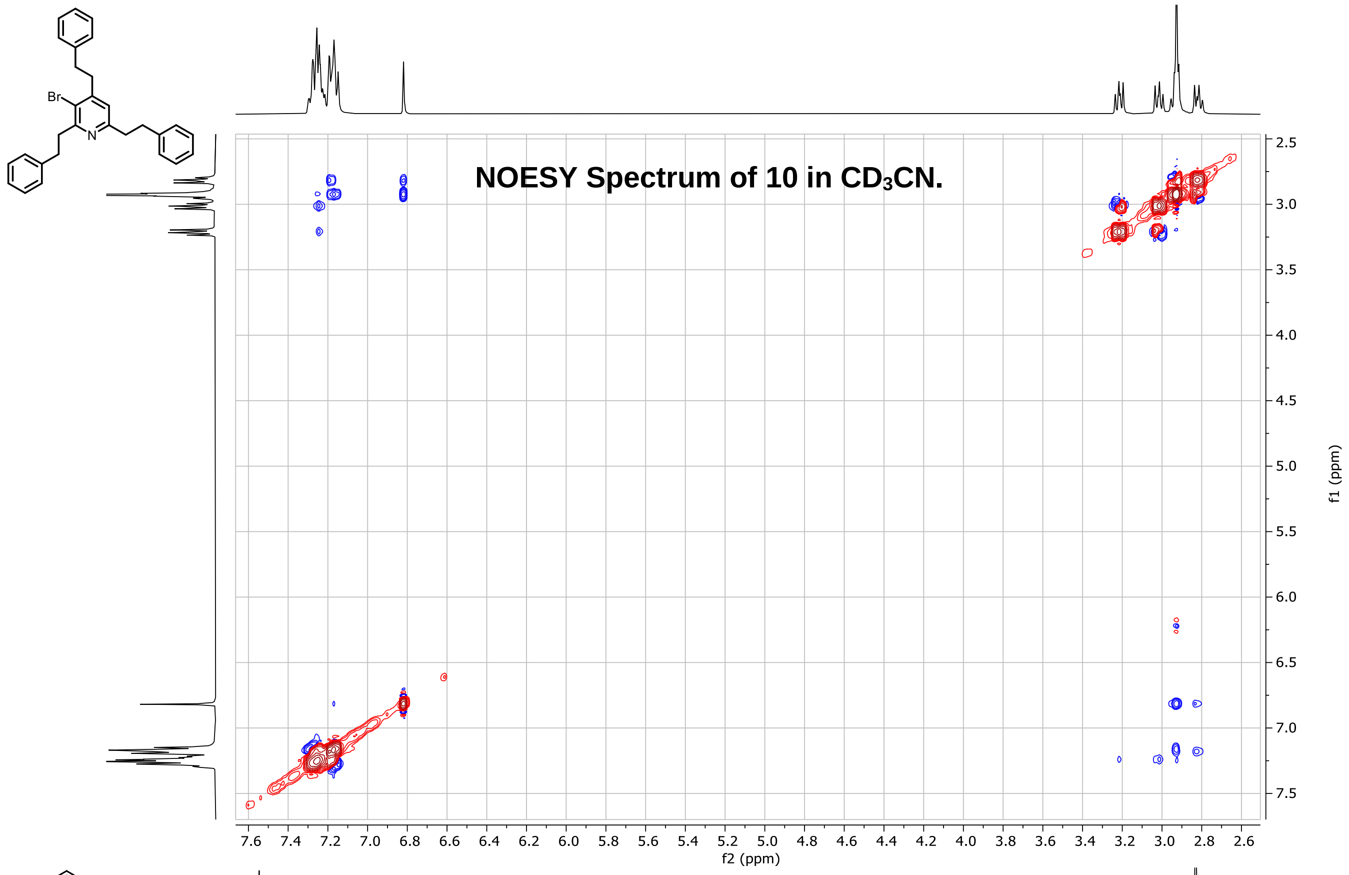
NOESY Spectrum of 10 in CD_3CN.
Br
N
f2 (ppm)
f1 (ppm)

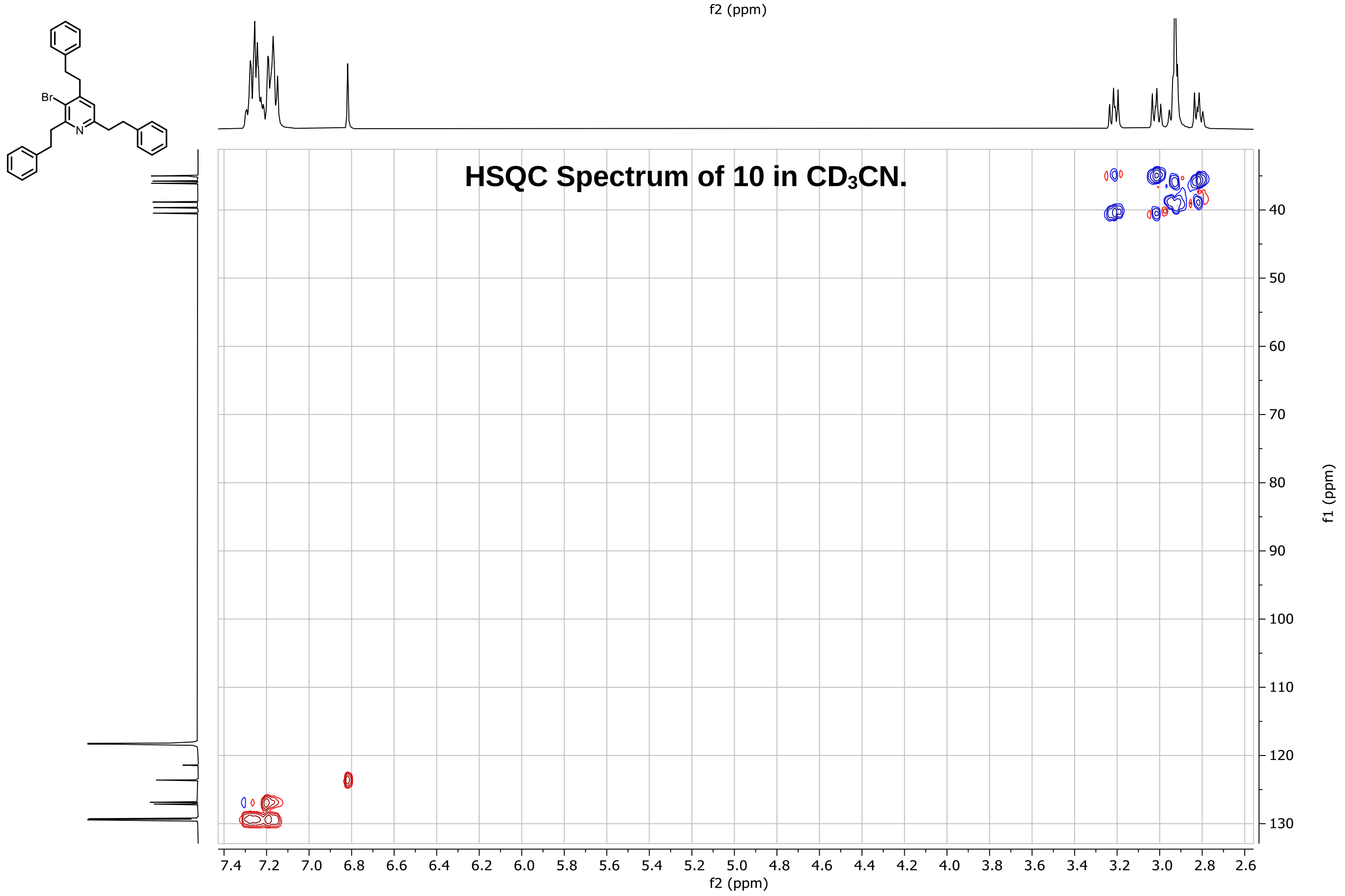
HSQC Spectrum of 10 in CD_3CN.
Br
N
f2 (ppm)
f1 (ppm)

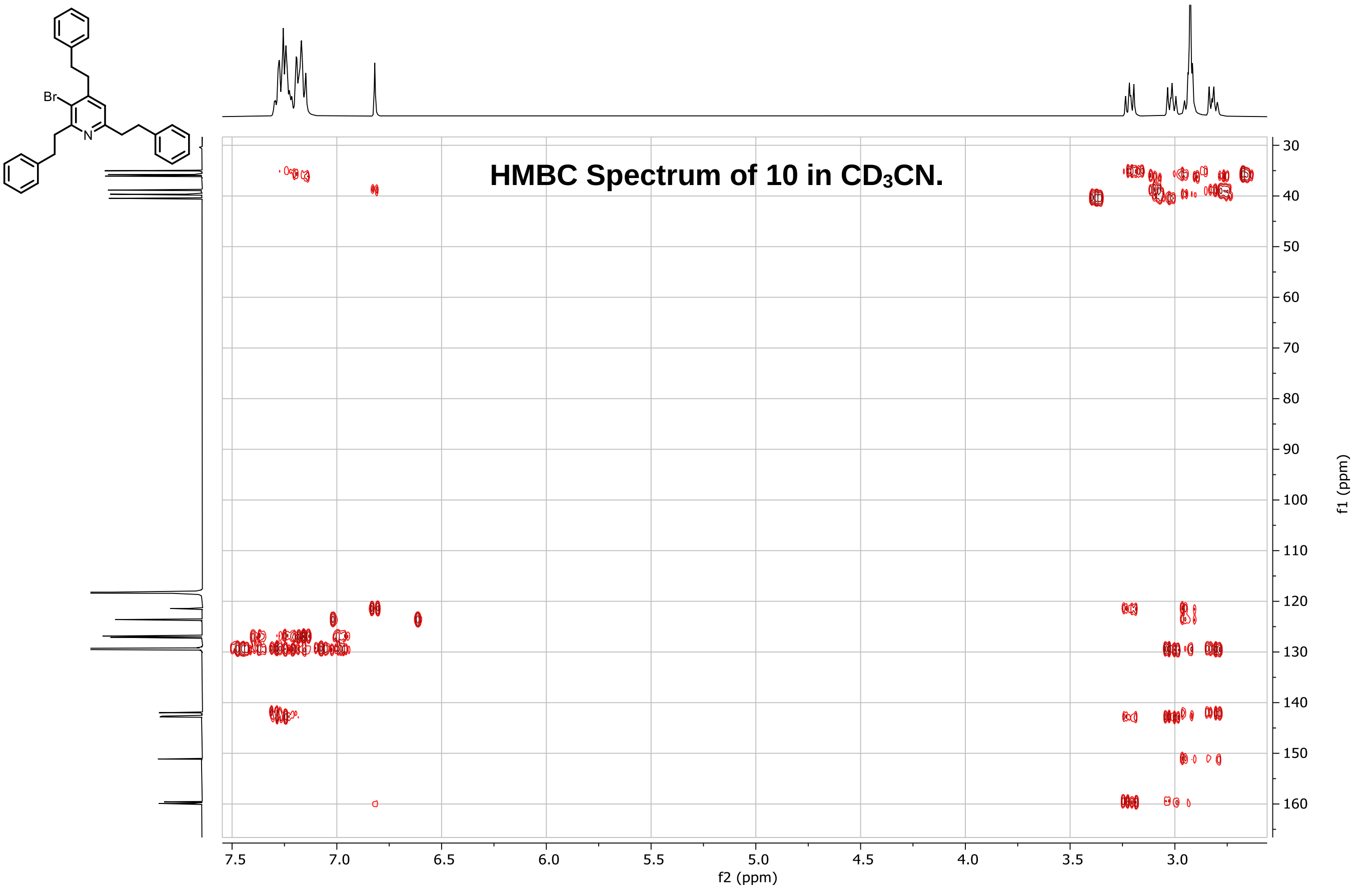
HMBC Spectrum of 10 in CD3CN.
Br
N
f2 (ppm)
f1 (ppm)

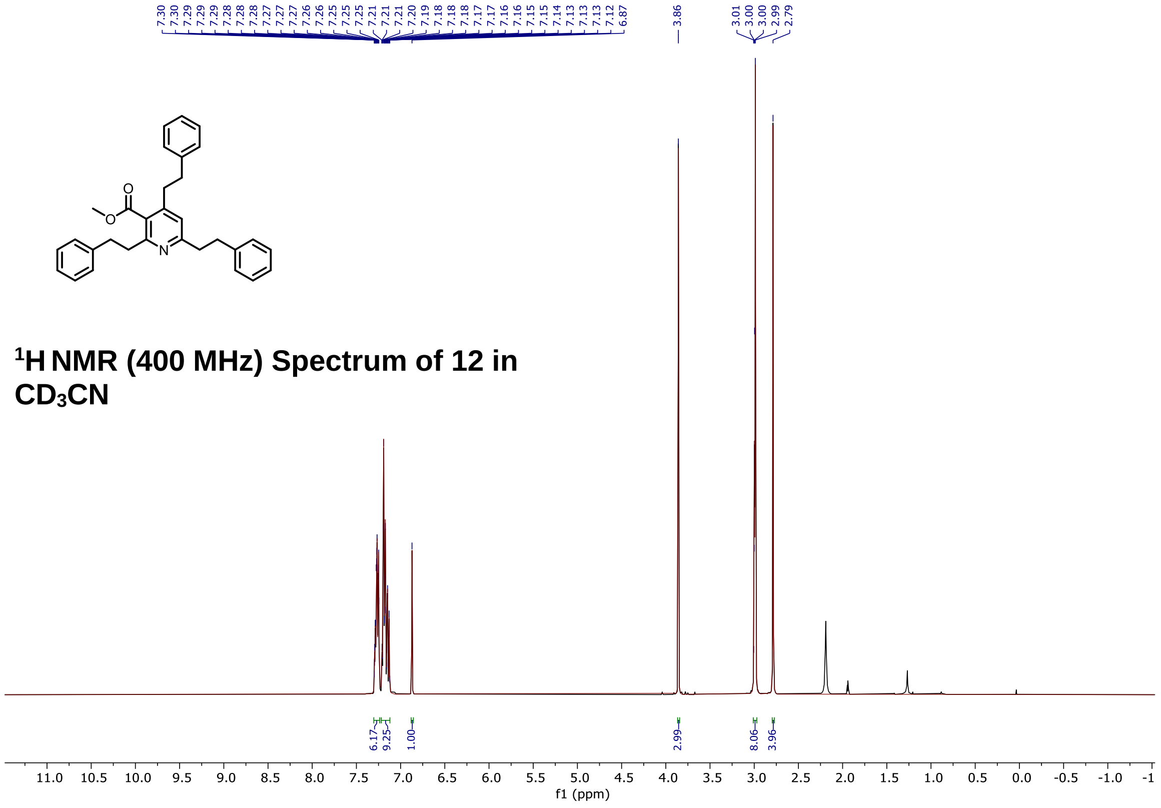
1H NMR (400 MHz) Spectrum of 12 in CD3CN
O
O
N
f1 (ppm)

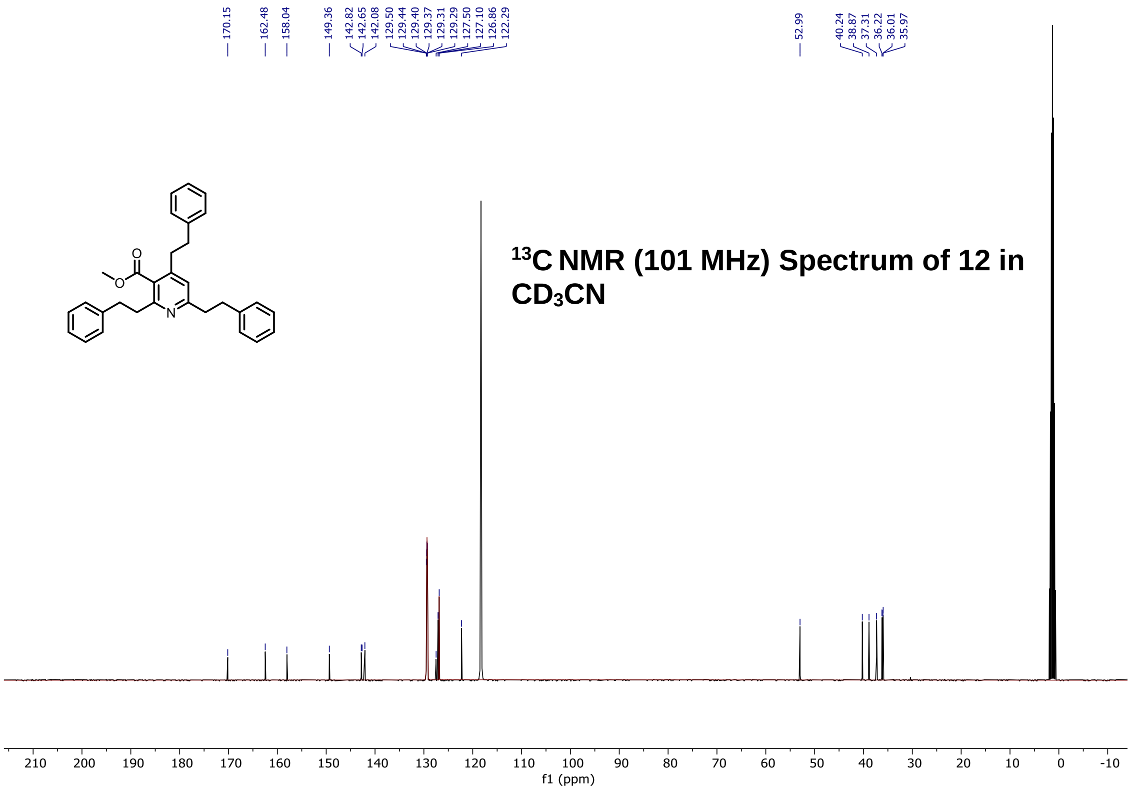

^{13}C NMR (101 MHz) Spectrum of 12 in CD_3CN
f1 (ppm)

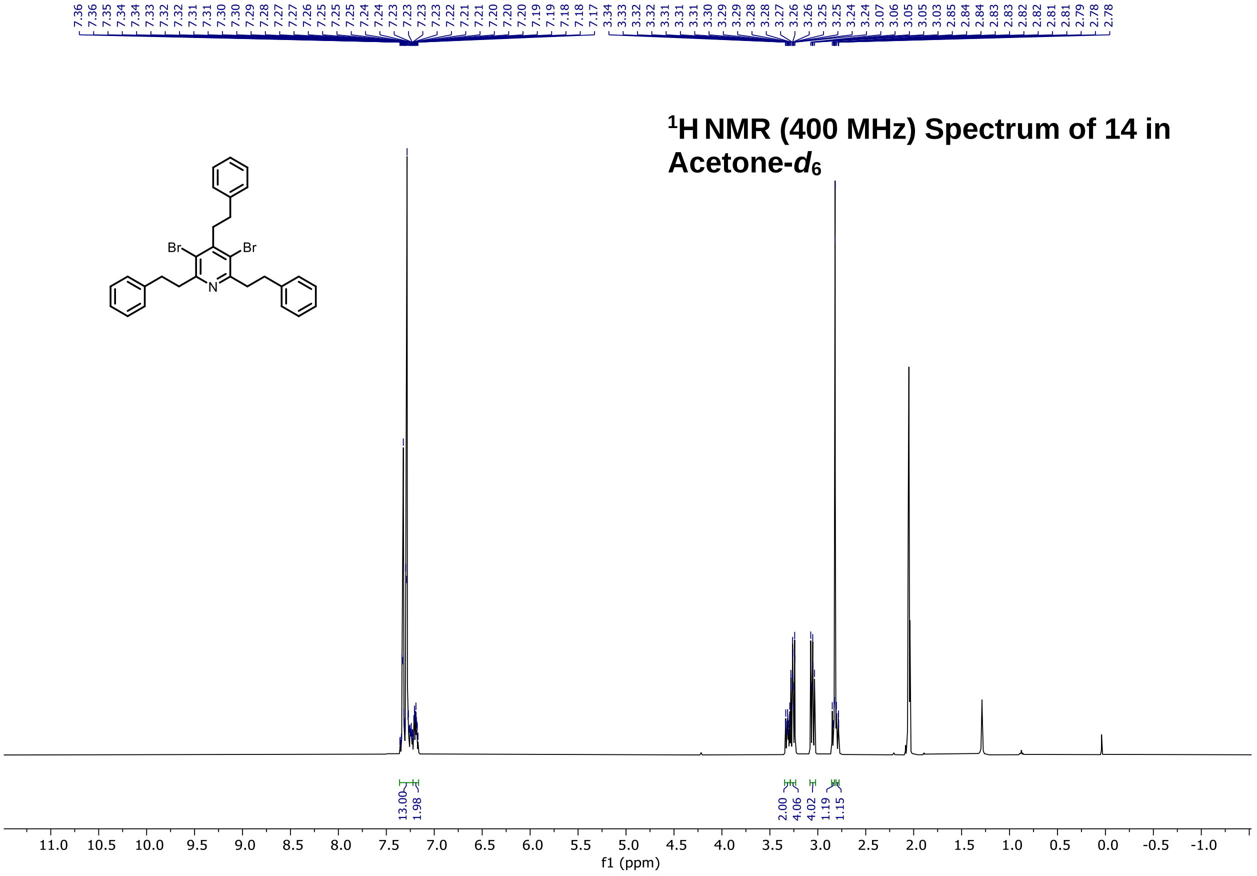

^{1}H NMR (400 MHz) Spectrum of 14 in Acetone-d_6
Br
N
f1 (ppm)

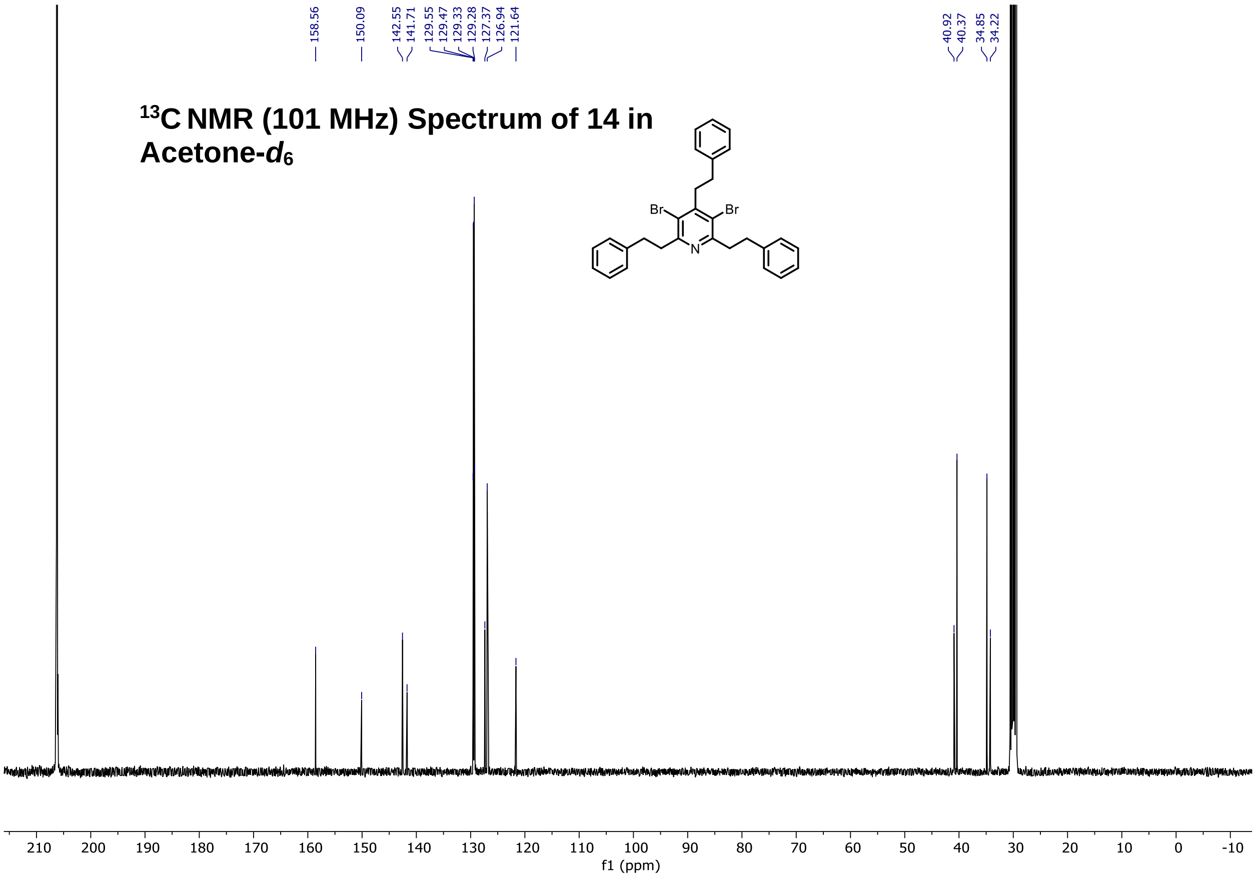
158.56
150.09
142.55
141.71
129.55
129.47
129.33
129.28
127.37
126.94
121.64
40.92
40.37
34.85
34.22
^{13}C NMR (101 MHz) Spectrum of 14 in Acetone-d_6
Br
Br
N
210 200 190 180 170 160 150 140 130 120 110 100 90 80 70 60 50 40 30 20 10 0 -10
f1 (ppm)

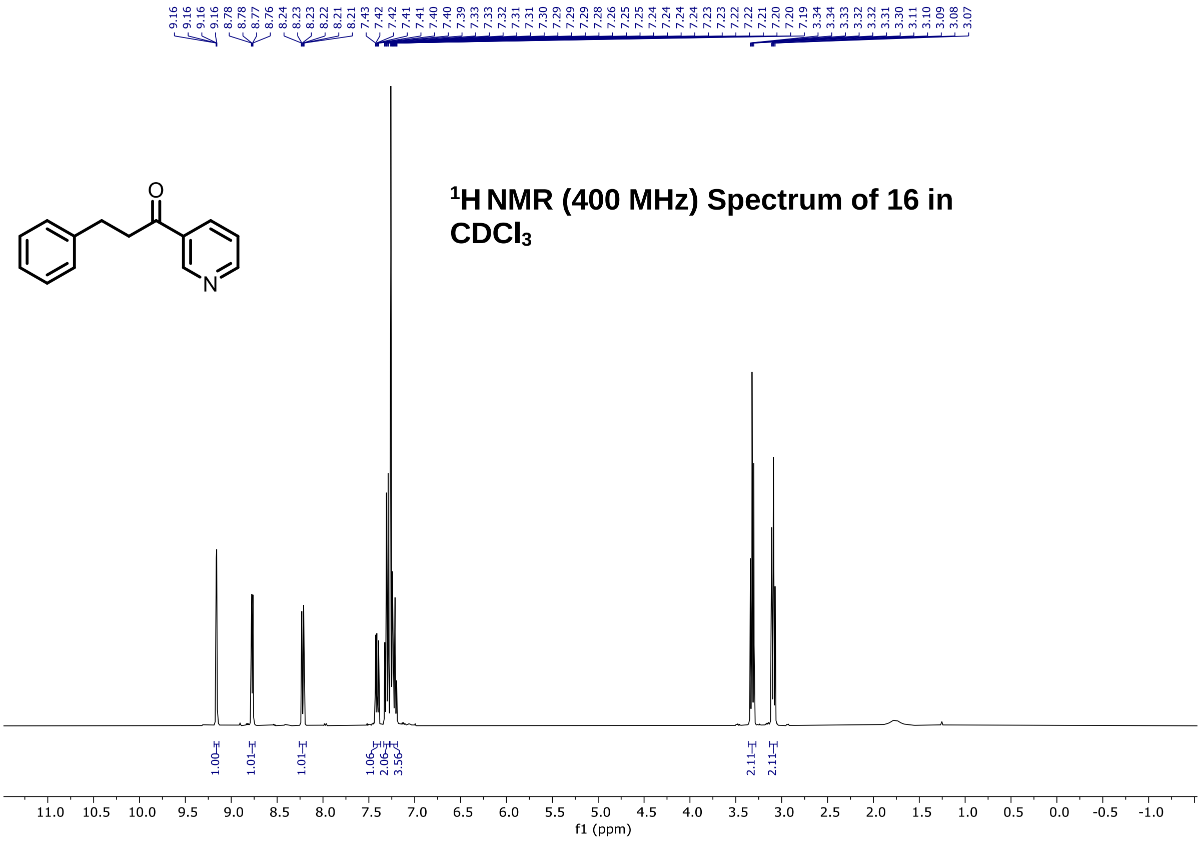
9.16
9.16
9.16
9.16
8.78
8.78
8.77
8.76
8.24
8.23
8.23
8.22
8.21
8.21
7.43
7.42
7.42
7.41
7.41
7.40
7.40
7.39
7.33
7.33
7.32
7.31
7.31
7.30
7.29
7.29
7.29
7.28
7.26
7.25
7.25
7.24
7.24
7.24
7.24
7.23
7.23
7.22
7.22
7.21
7.20
7.19
3.34
3.34
3.33
3.32
3.32
3.31
3.30
3.11
3.10
3.09
3.08
3.07
^{1}H NMR (400 MHz) Spectrum of 16 in $CDCl_3$
O
N
1.00
1.01
1.01
1.06
2.06
3.56
2.11
2.11
11.0 10.5 10.0 9.5 9.0 8.5 8.0 7.5 7.0 6.5 6.0 5.5 5.0 4.5 4.0 3.5 3.0 2.5 2.0 1.5 1.0 0.5 0.0 -0.5 -1.0
f1 (ppm)

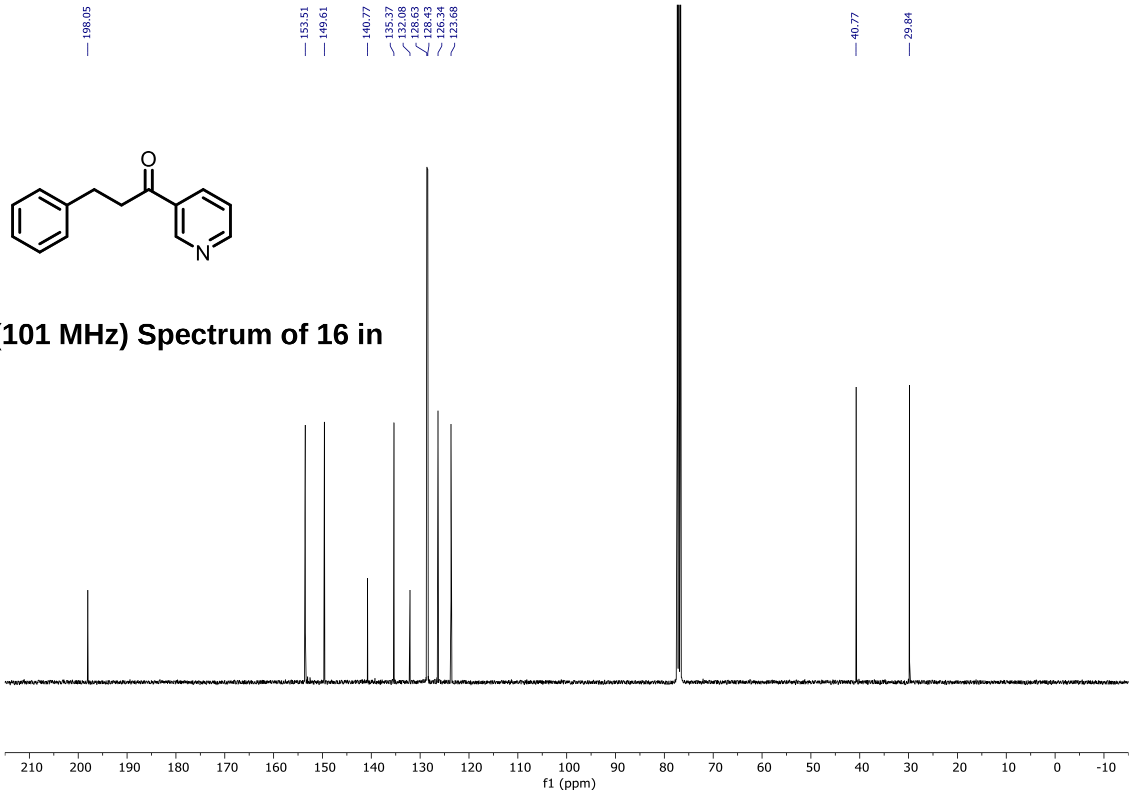

^{13}C NMR (101 MHz) Spectrum of 16 in $CDCl_3$

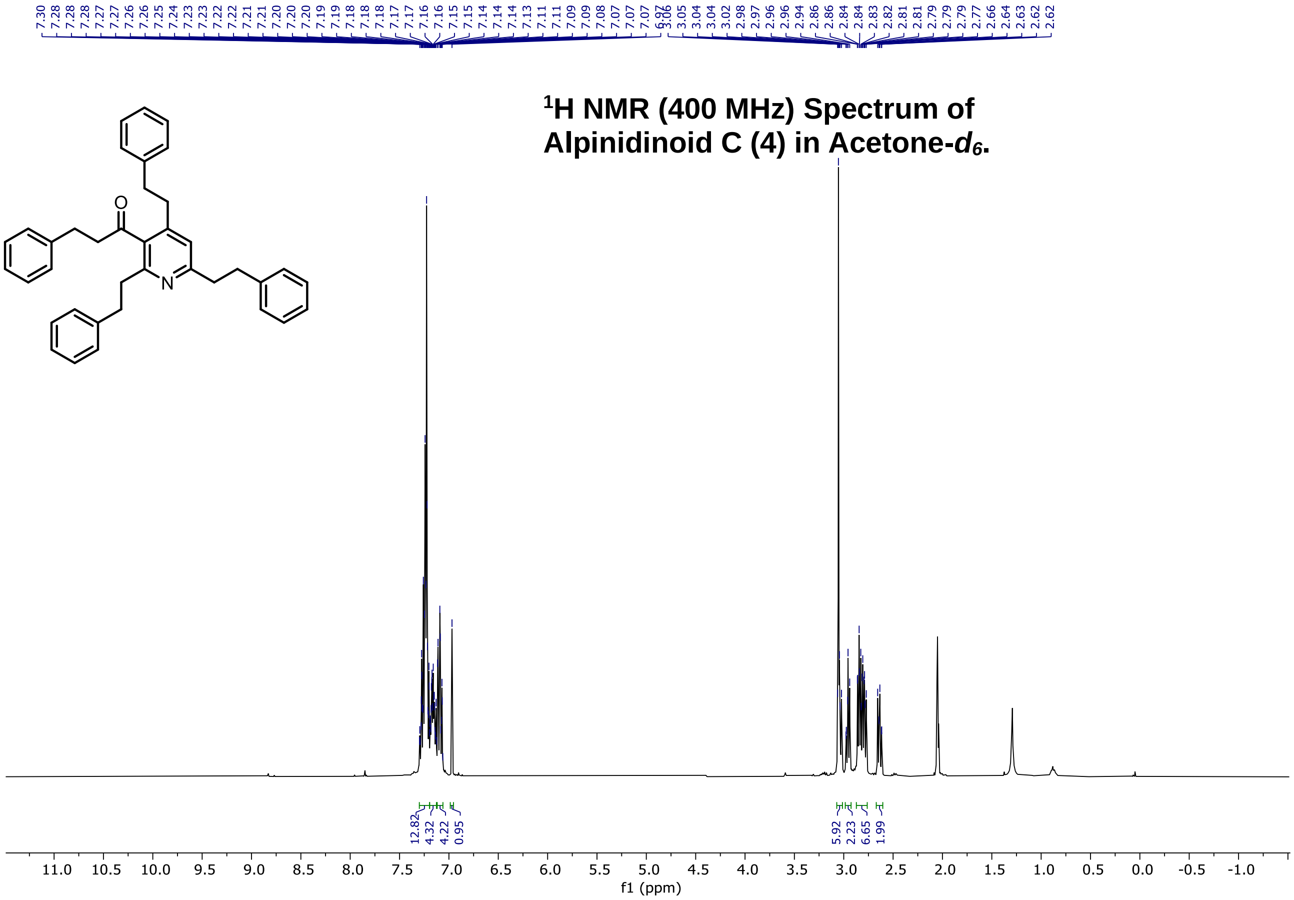

^{1}H NMR (400 MHz) Spectrum of Alpinidinoid C (4) in Acetone-d_6.

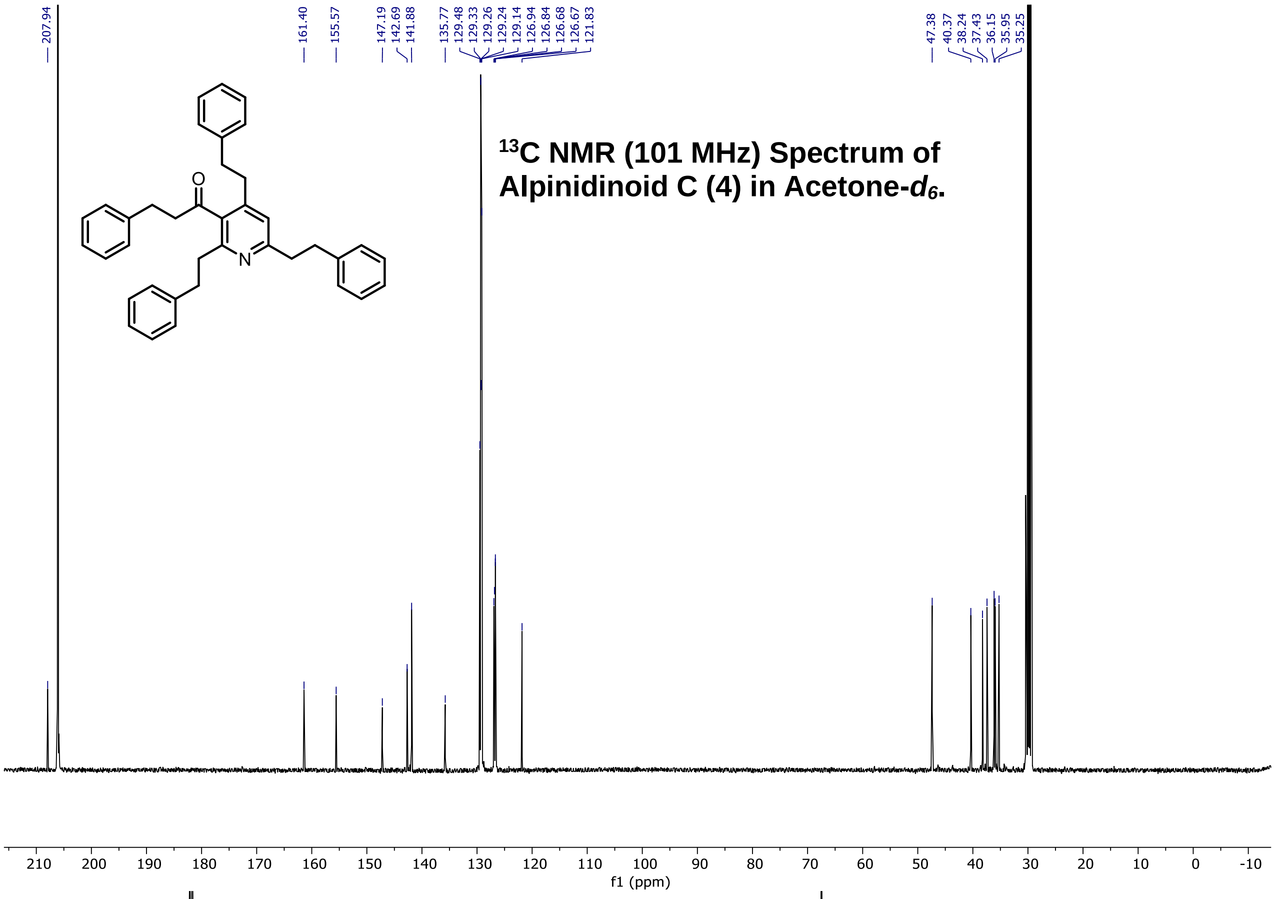

^{13}C NMR (101 MHz) Spectrum of Alpinidinoid C (4) in Acetone-d_6.

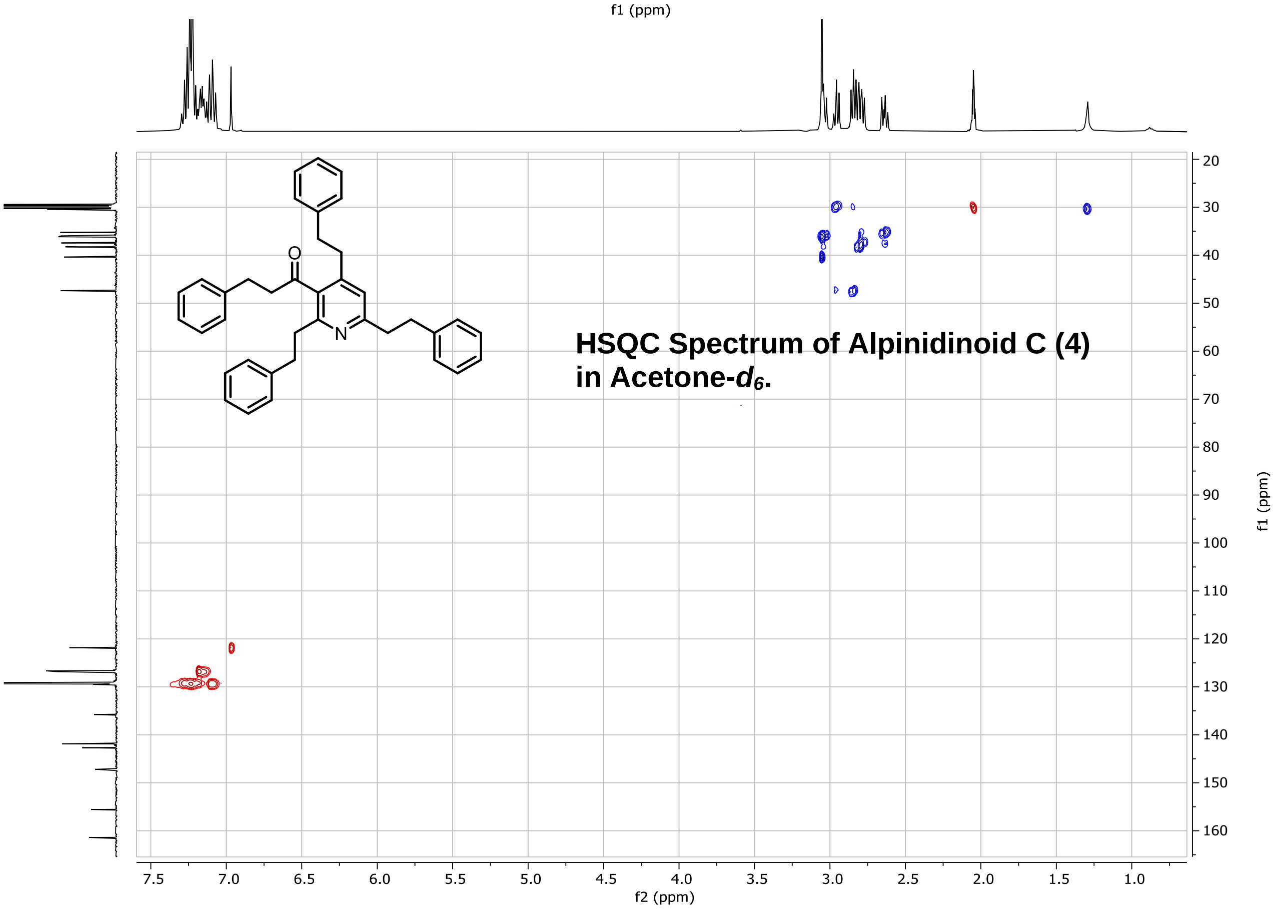

HSQC Spectrum of Alpinidinoid C (4) in Acetone-d_6.

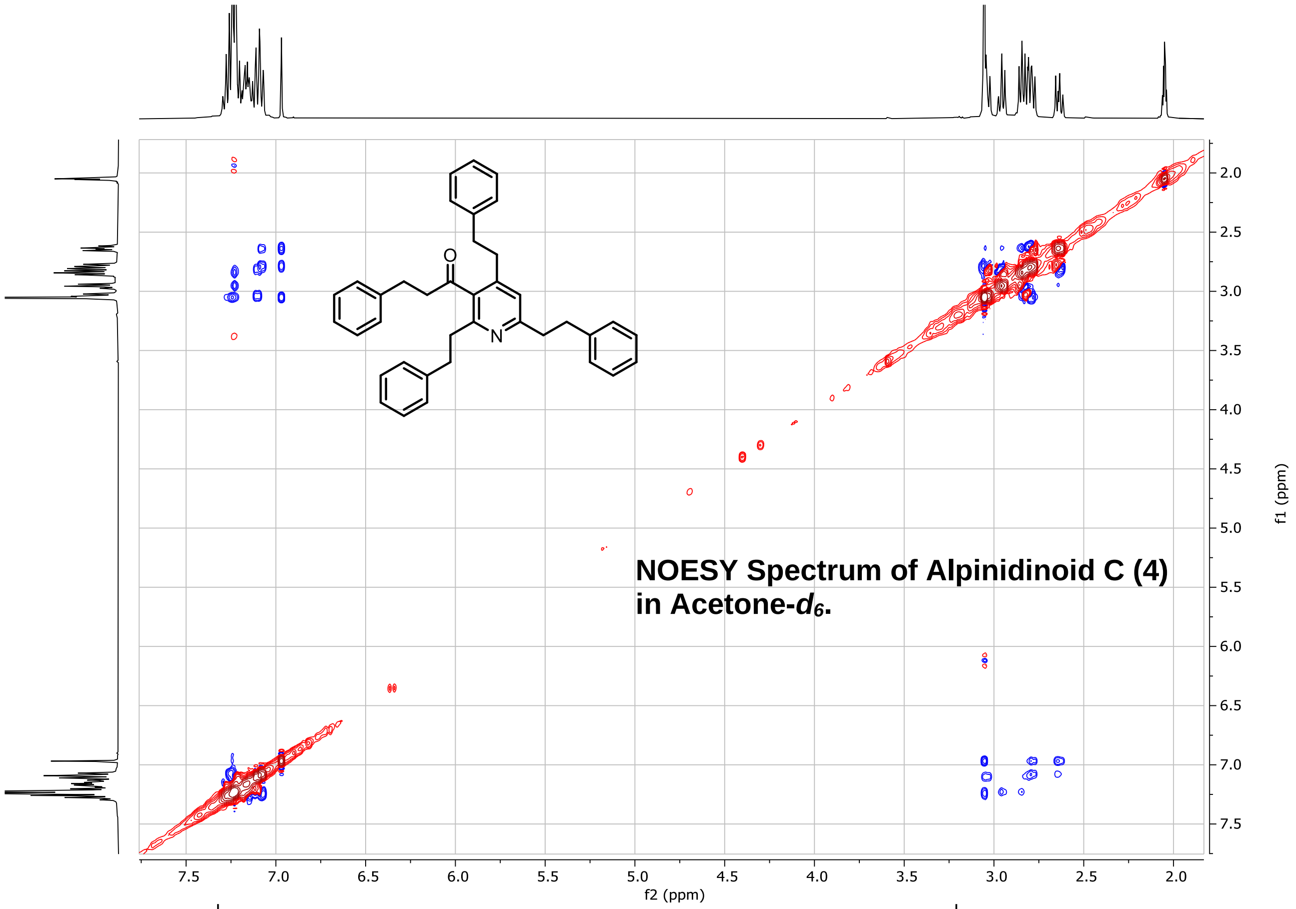
NOESY Spectrum of Alpinidinoid C (4) in Acetone-d_6.
f2 (ppm)
f1 (ppm)

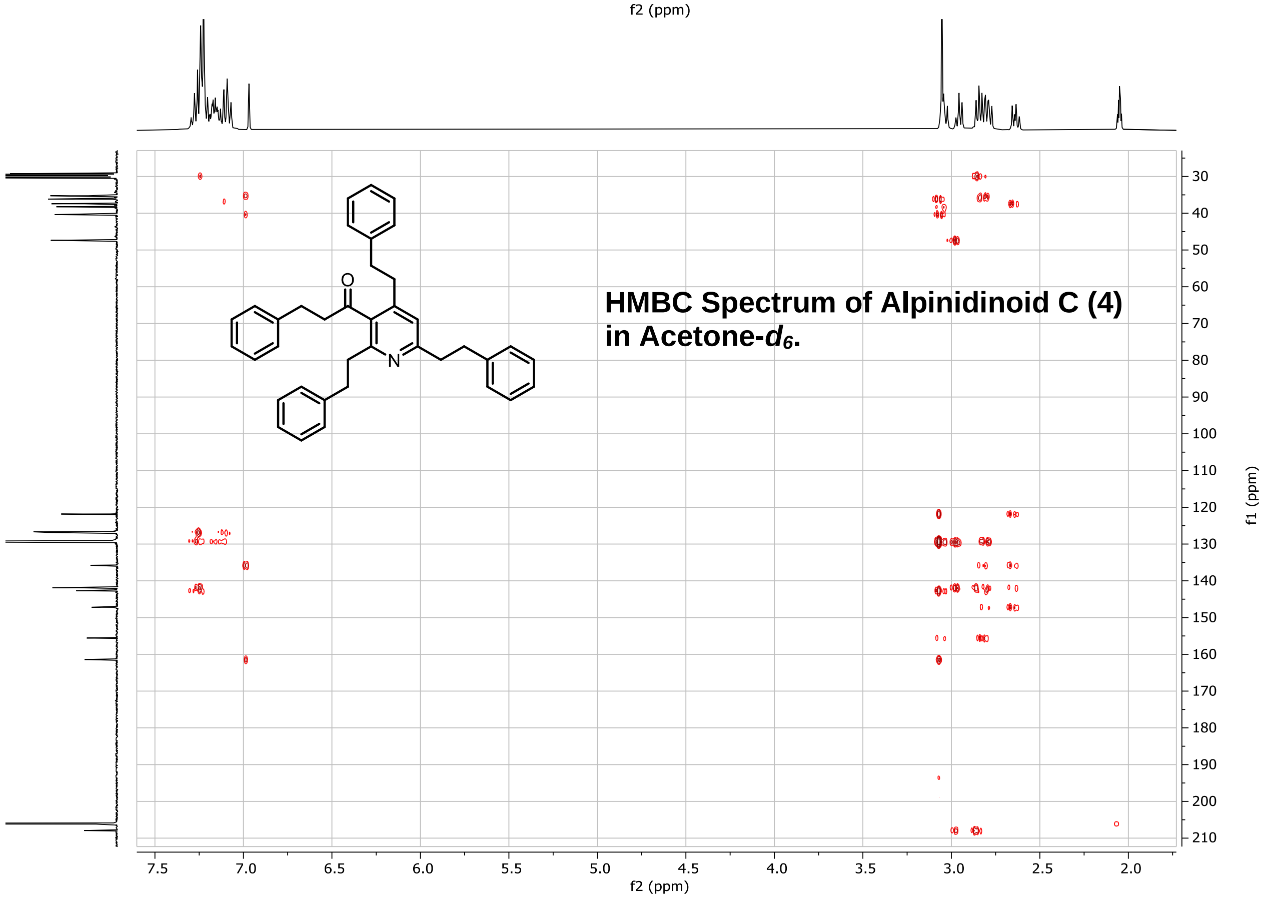
HMBC Spectrum of Alpinidinoid C (4) in Acetone-d_6.
f2 (ppm)
f1 (ppm)

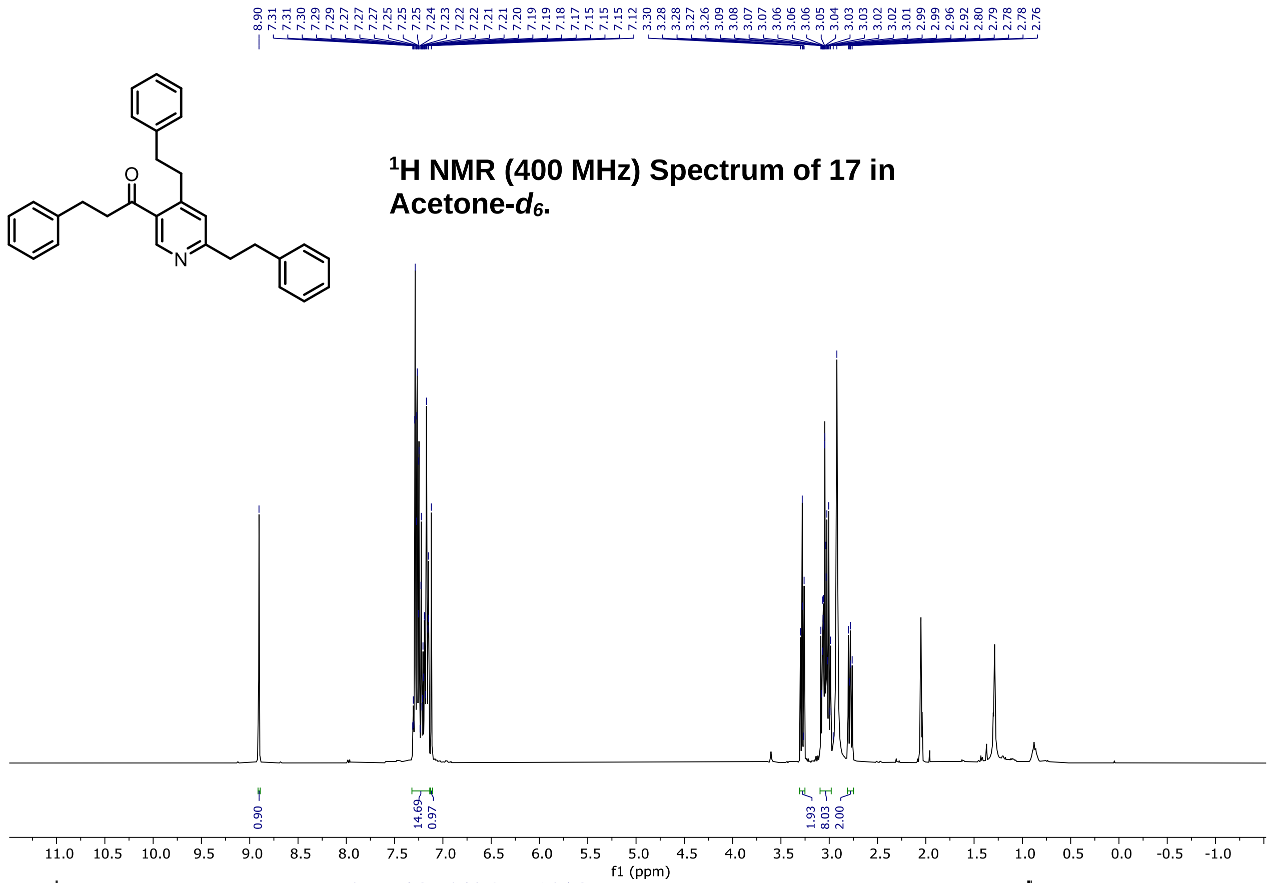

^{1}H NMR (400 MHz) Spectrum of 17 in Acetone-d_6.

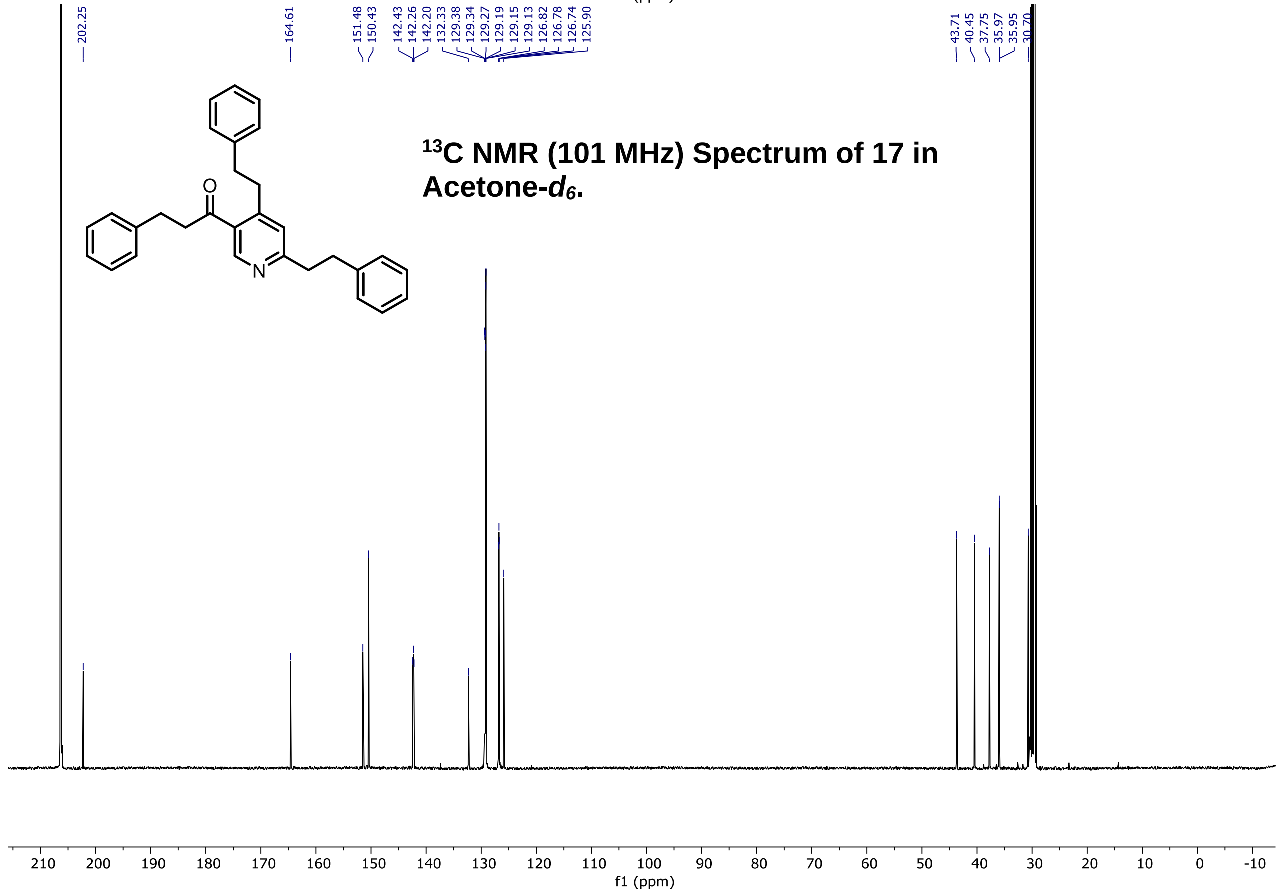

^{13}C NMR (101 MHz) Spectrum of 17 in Acetone-d_6.

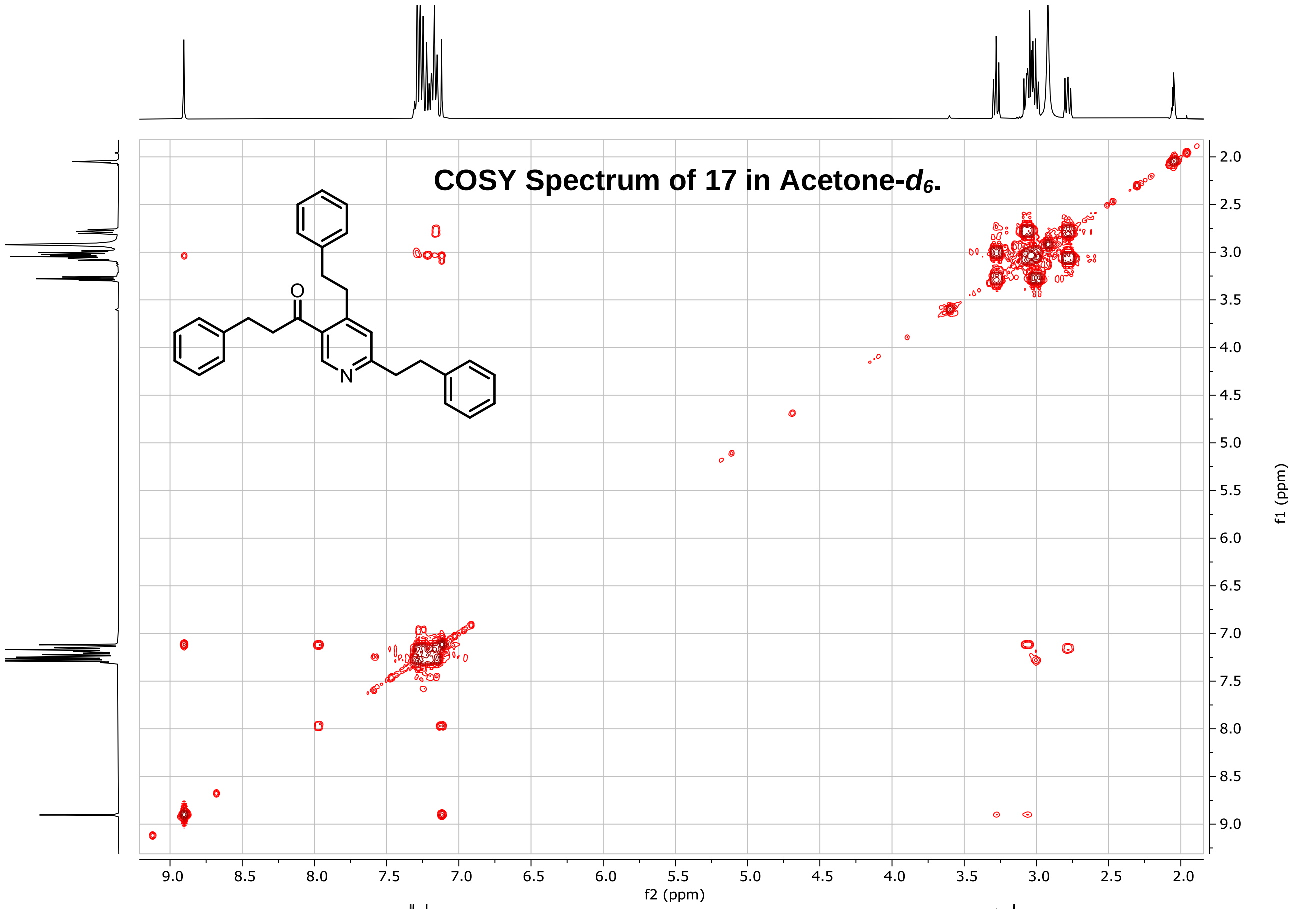
COSY Spectrum of 17 in Acetone-d_6.
O
N
f2 (ppm)
f1 (ppm)

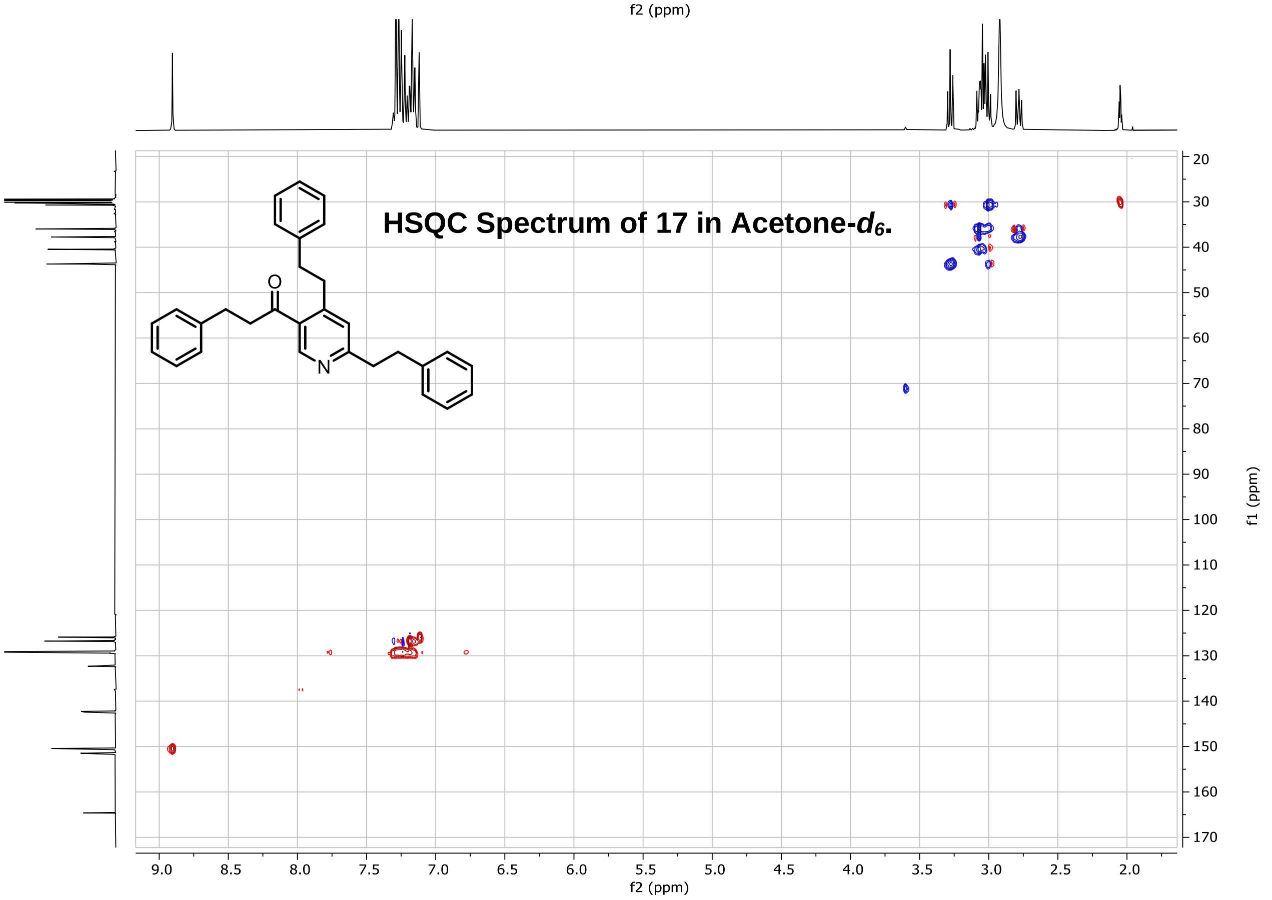
HSQC Spectrum of 17 in Acetone-d_6.
O
N
f2 (ppm)
f1 (ppm)

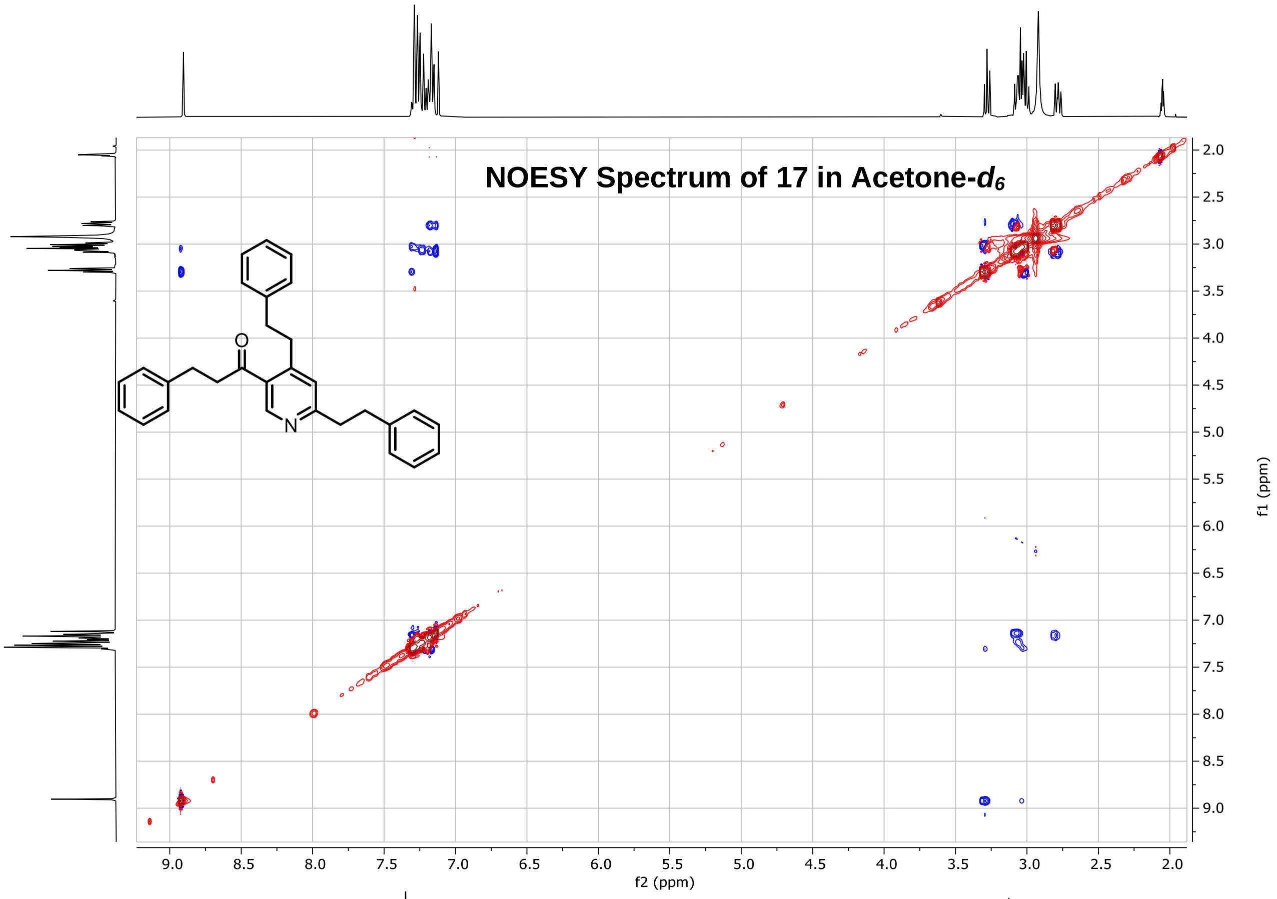
NOESY Spectrum of 17 in Acetone-d_6
O
N
f1 (ppm)
f2 (ppm)

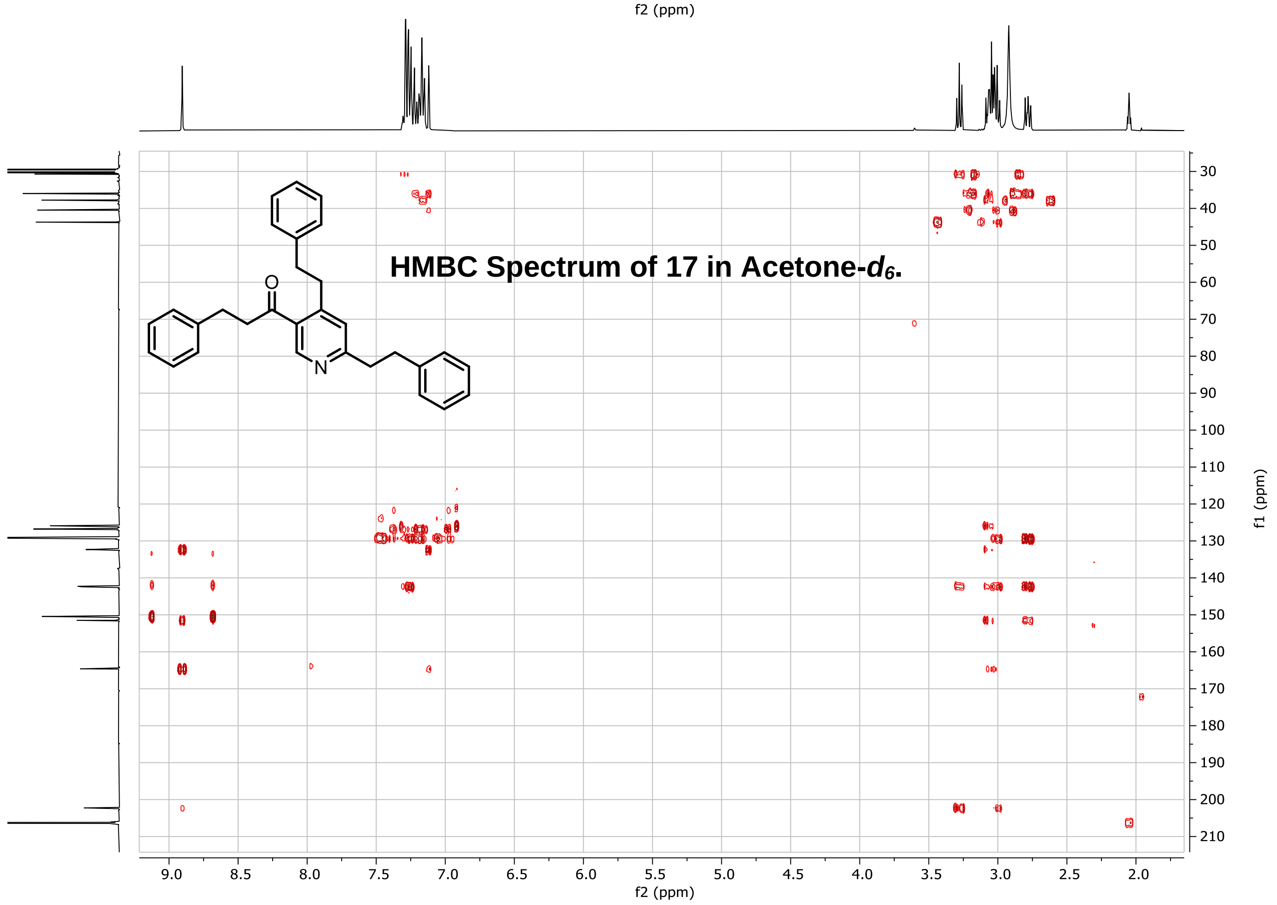
HMBC Spectrum of 17 in Acetone-d_6.
O
N
f1 (ppm)
f2 (ppm)

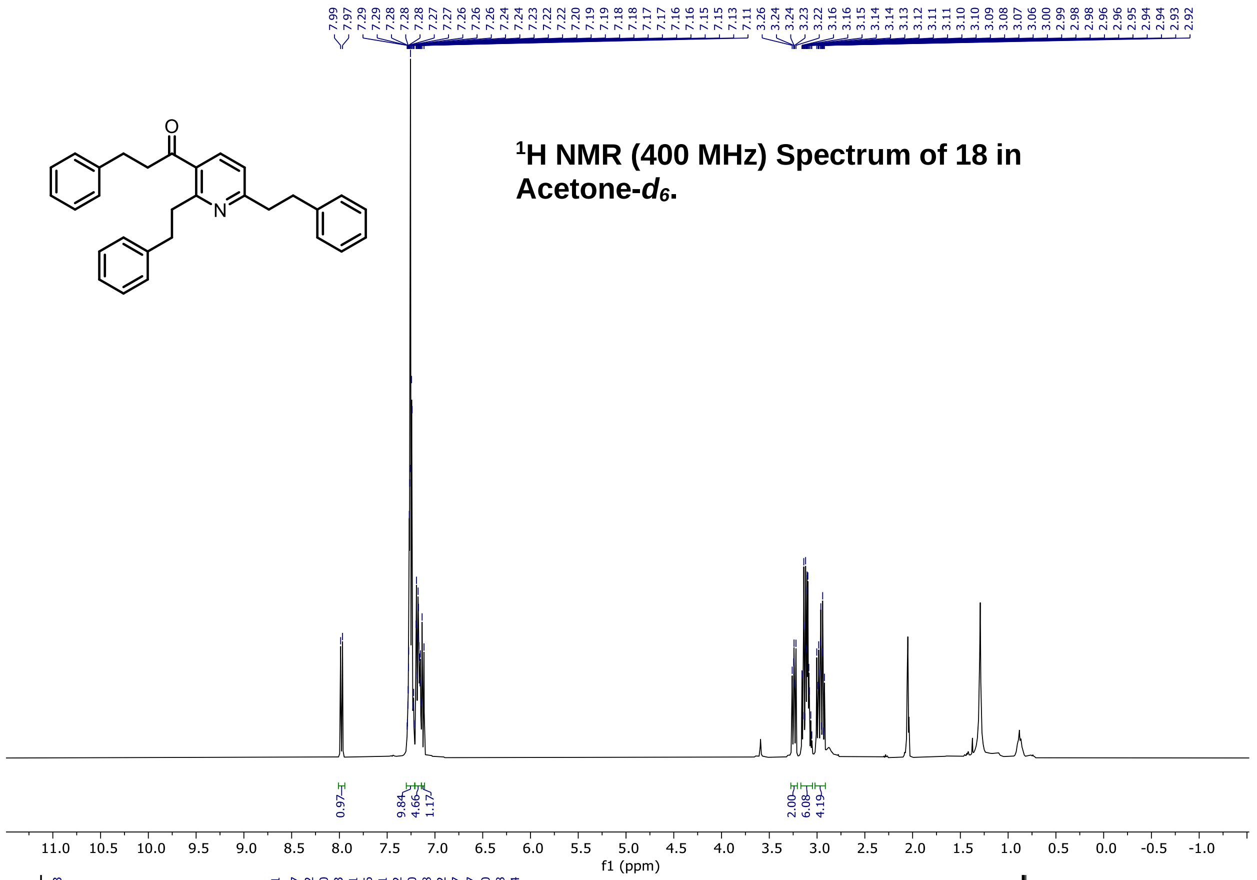
7.99 7.97 7.29 7.29 7.28 7.28 7.28 7.28 7.27 7.27 7.26 7.26 7.26 7.24 7.24 7.23 7.22 7.22 7.20 7.19 7.19 7.18 7.18 7.17 7.17 7.16 7.16 7.15 7.15 7.13 7.11 3.26 3.24 3.24 3.23 3.22 3.16 3.16 3.15 3.14 3.13 3.12 3.11 3.10 3.10 3.09 3.08 3.07 3.06 3.00 2.99 2.98 2.98 2.96 2.95 2.94 2.94 2.93 2.92
O
N
^{1}H NMR (400 MHz) Spectrum of 18 in Acetone-d_6.
0.97 9.84 4.66 1.17 2.00 6.08 4.19
11.0 10.5 10.0 9.5 9.0 8.5 8.0 7.5 7.0 6.5 6.0 5.5 5.0 4.5 4.0 3.5 3.0 2.5 2.0 1.5 1.0 0.5 0.0 -0.5 -1.0
f1 (ppm)

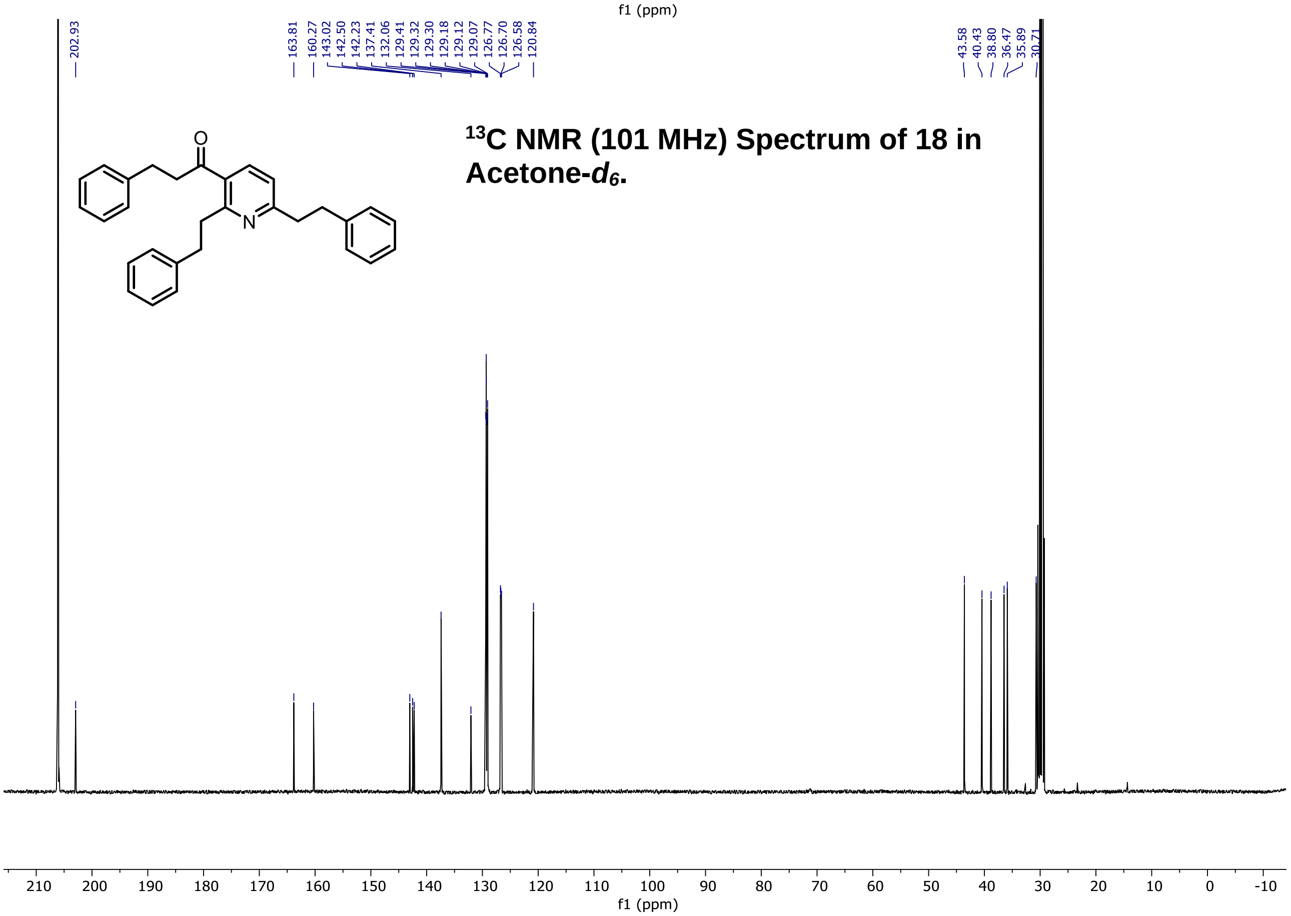
202.93 163.81 160.27 143.02 142.50 142.23 137.41 132.06 129.41 129.32 129.30 129.18 129.12 129.07 126.77 126.70 126.58 120.84 43.58 40.43 38.80 36.47 35.89 30.71
O
N
^{13}C NMR (101 MHz) Spectrum of 18 in Acetone-d_6.
210 200 190 180 170 160 150 140 130 120 110 100 90 80 70 60 50 40 30 20 10 0 -10
f1 (ppm)

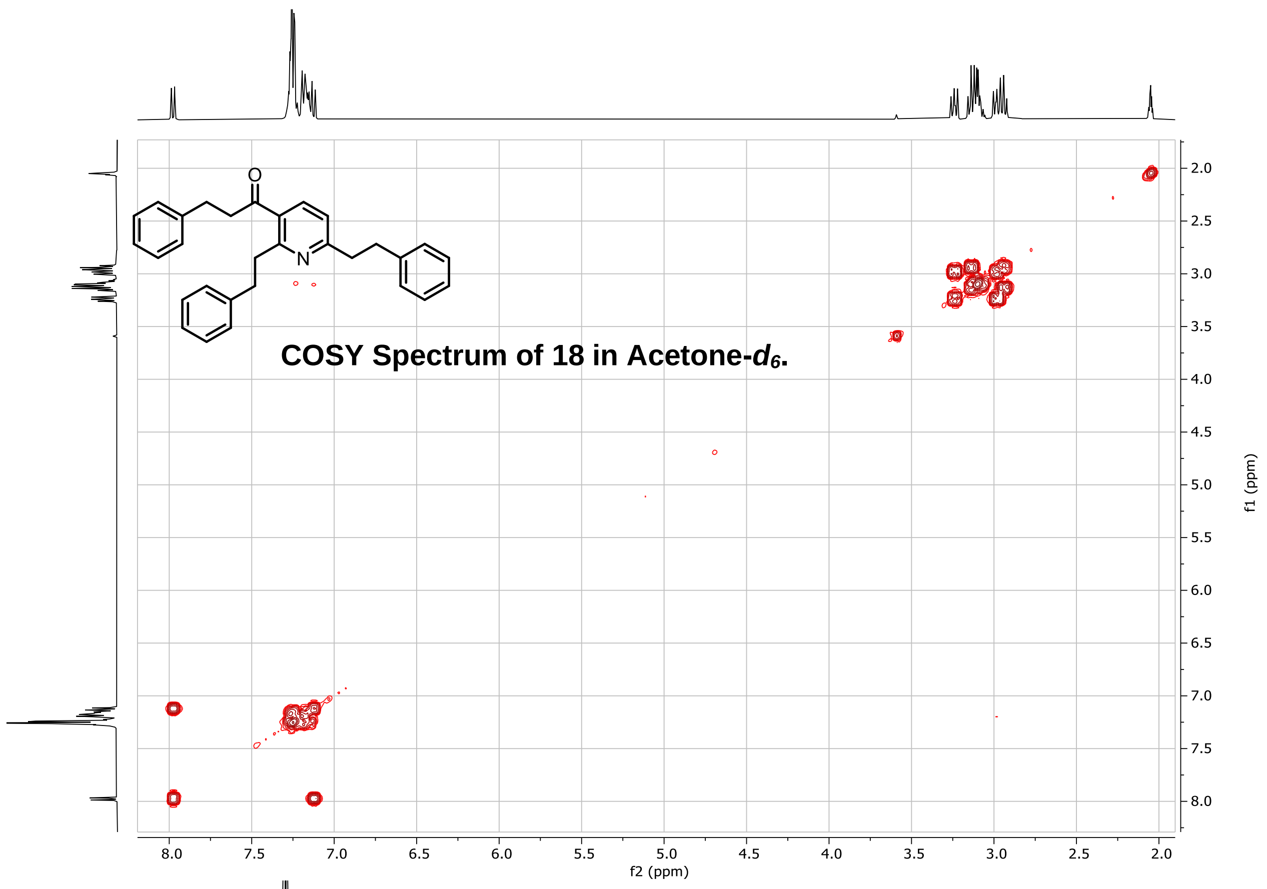

O
N
COSY Spectrum of 18 in Acetone-d_6.
f1 (ppm)
f2 (ppm)

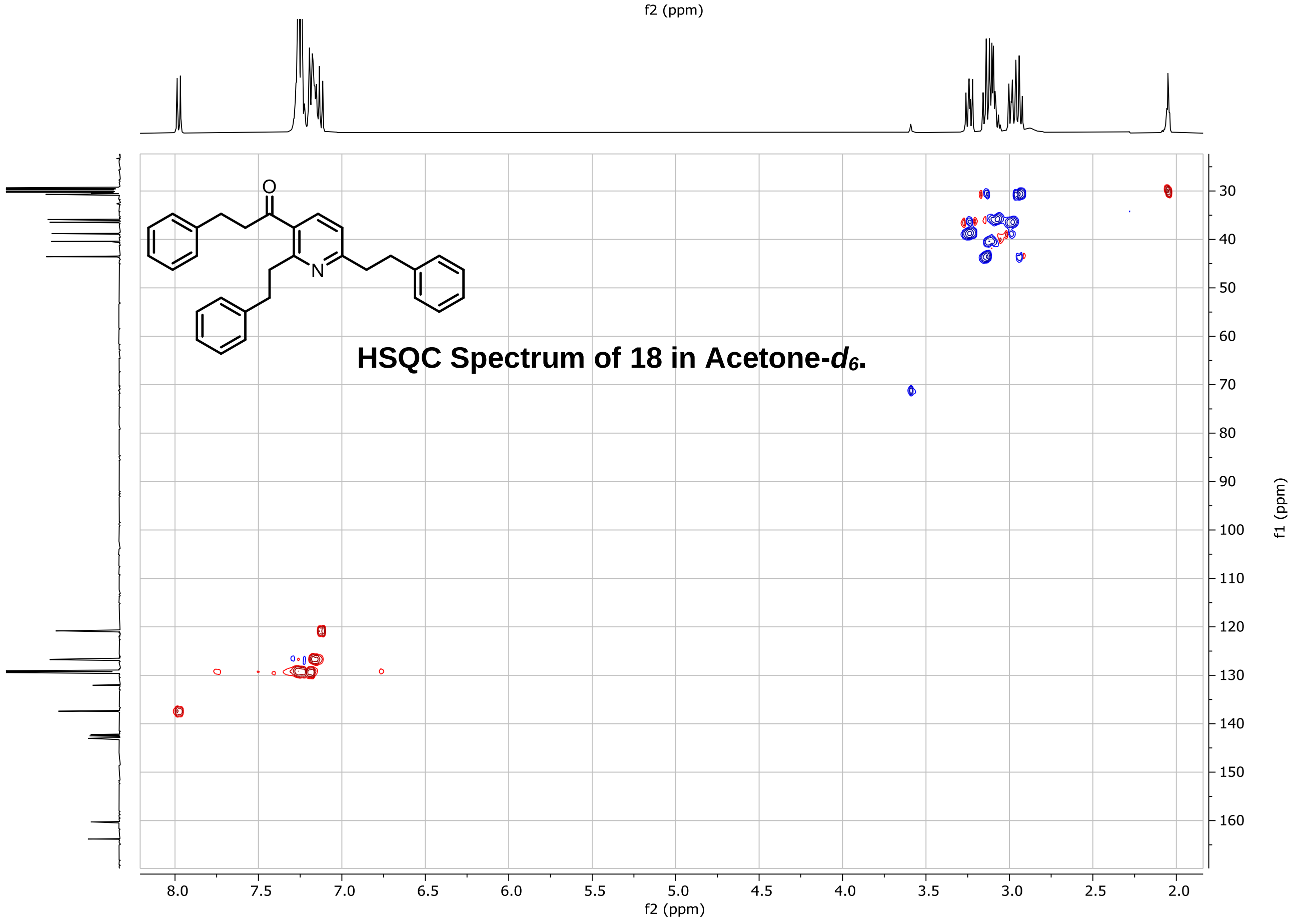

O
N
HSQC Spectrum of 18 in Acetone-d_6.
f1 (ppm)
f2 (ppm)

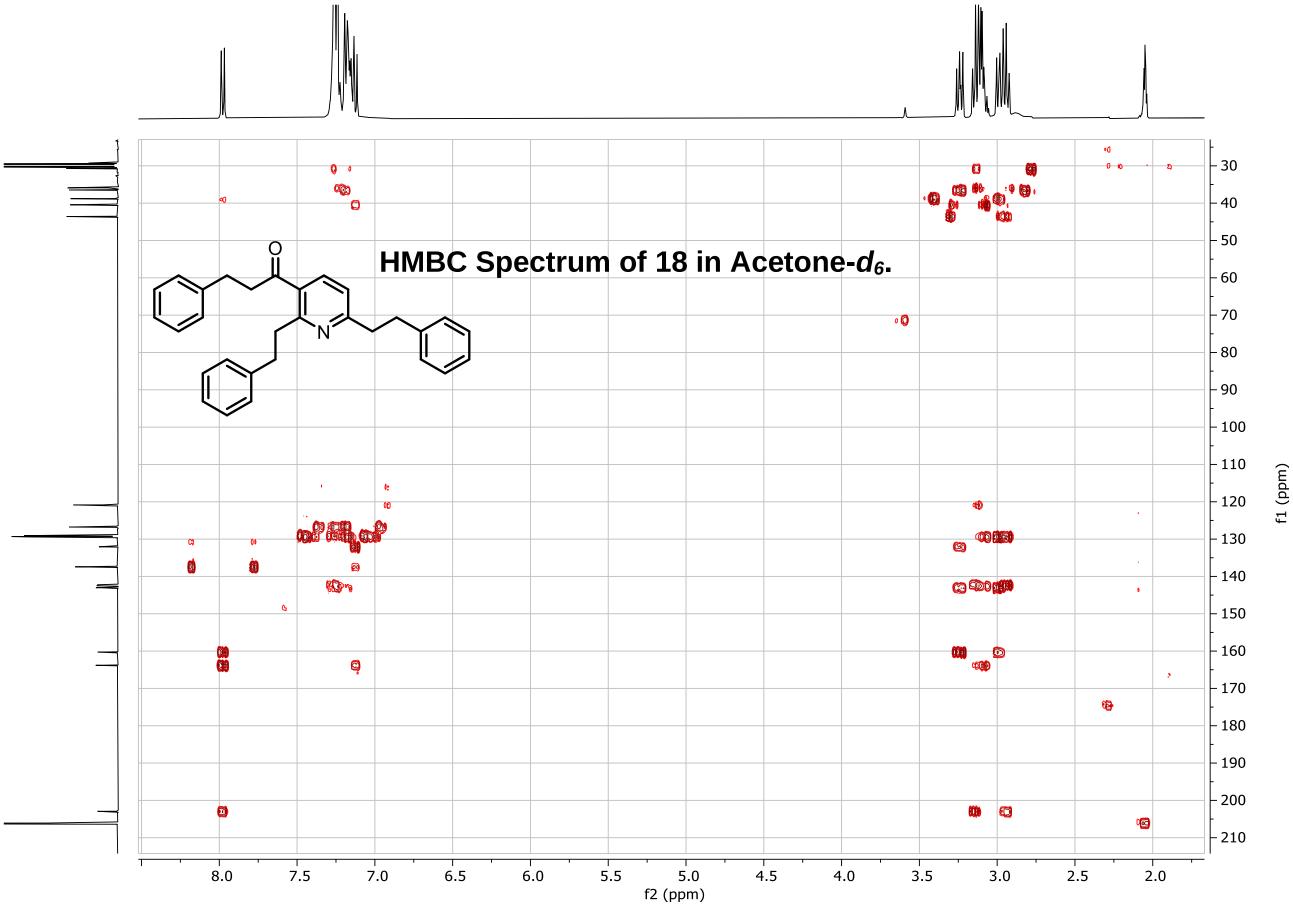
HMBC Spectrum of 18 in Acetone-d_6.
N
O
f2 (ppm)
f1 (ppm)

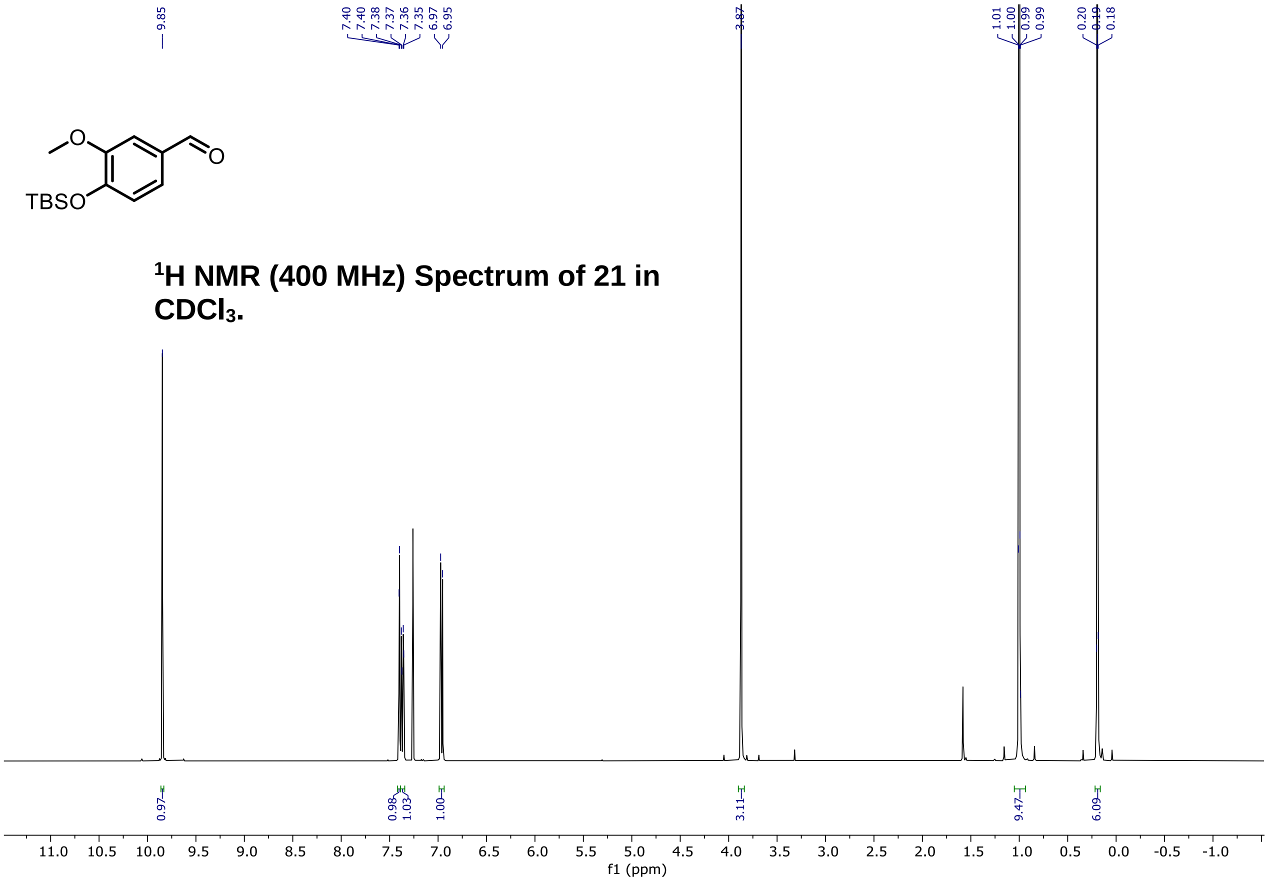
^{1}H NMR (400 MHz) Spectrum of 21 in CDCl$_3$.
TBSO
O
O
9.85
7.40
7.40
7.38
7.37
7.36
7.35
6.97
6.95
3.87
1.01
1.00
0.99
0.99
0.20
0.19
0.18
0.97
0.98
1.03
1.00
3.11
9.47
6.09
f1 (ppm)

^{13}C NMR (101 MHz) Spectrum of 21 in CDCl$_3$.

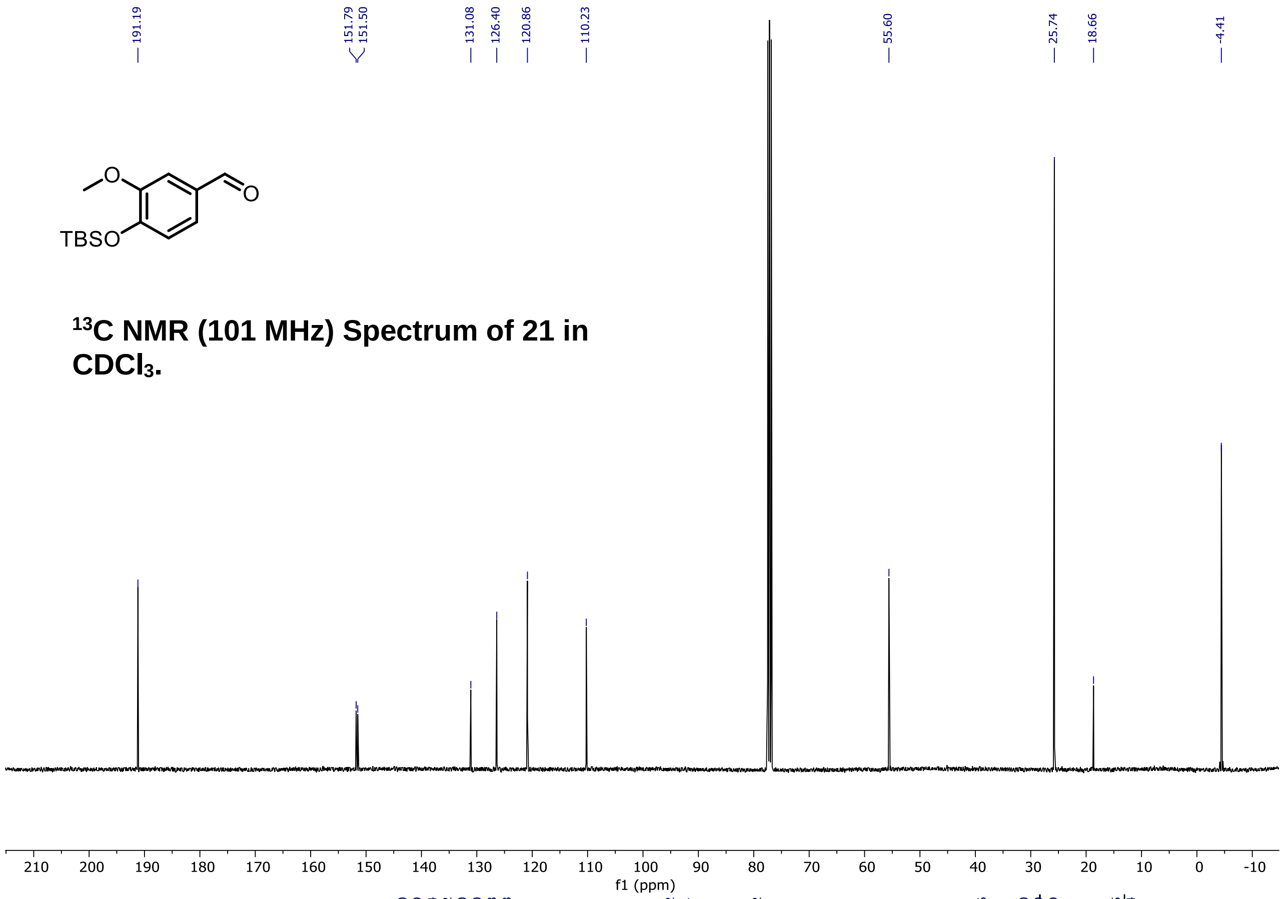

^{1}H NMR (400 MHz) Spectrum of 22 in CDCl$_3$.

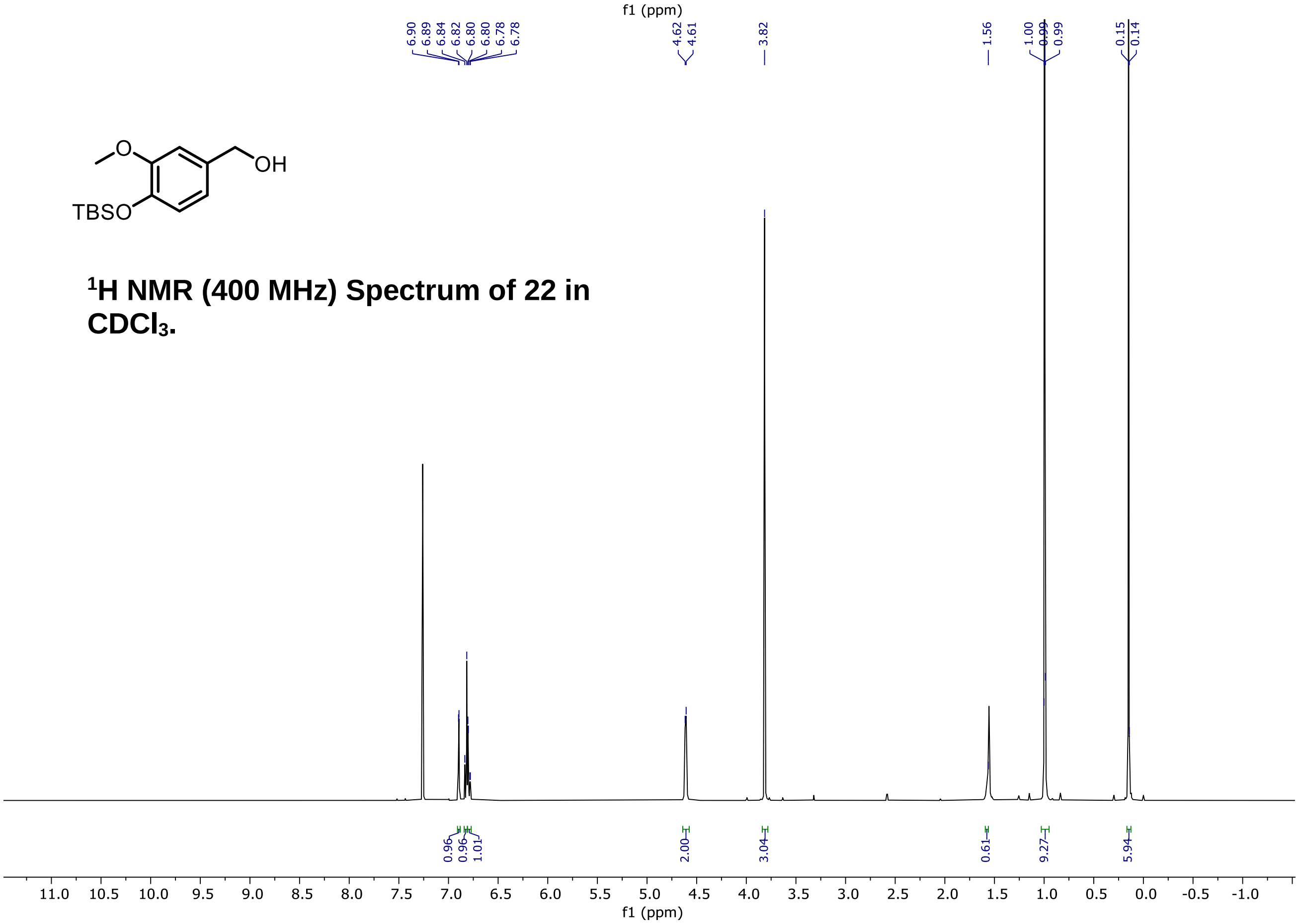

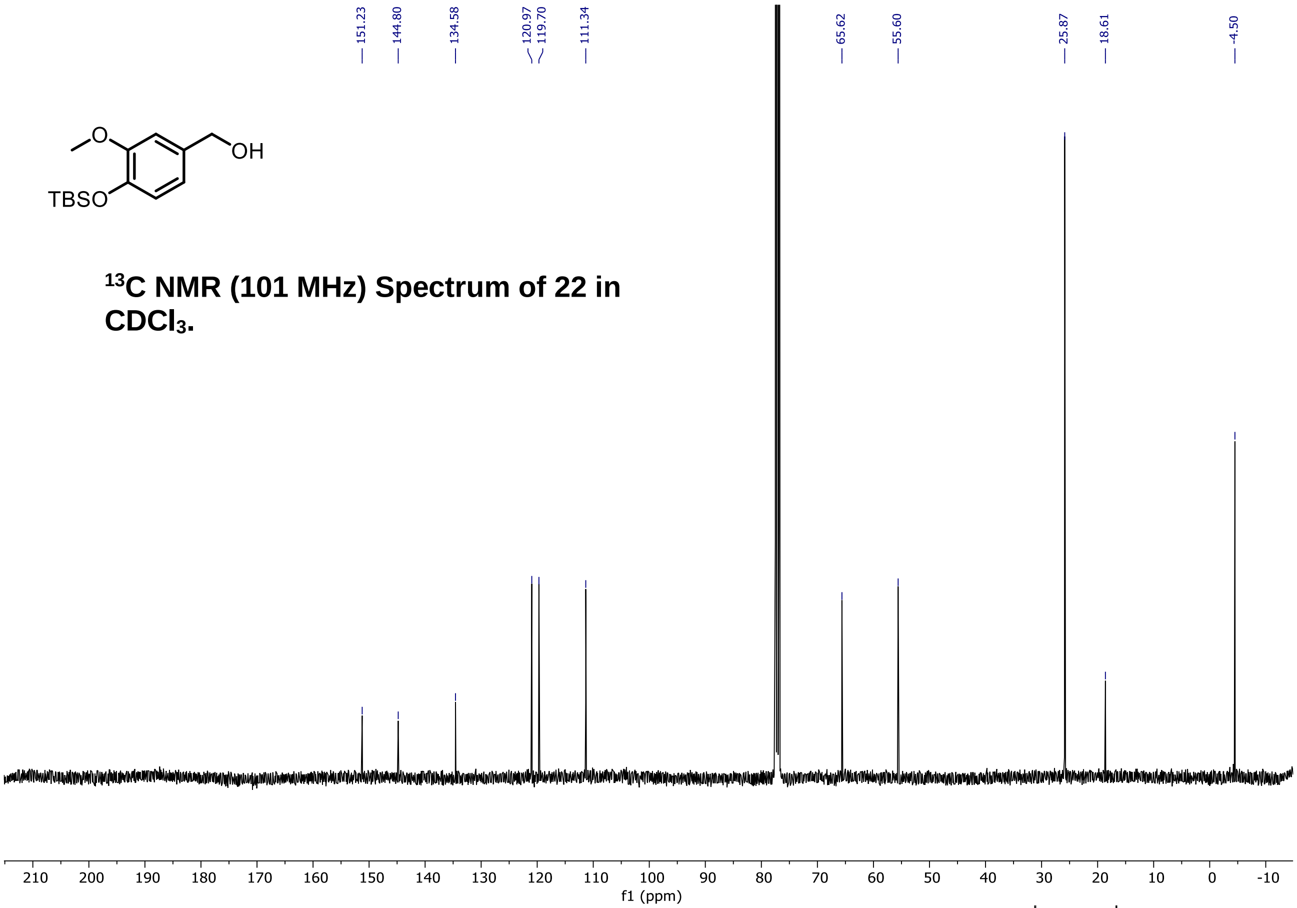

^{13}C NMR (101 MHz) Spectrum of 22 in $CDCl_3$.

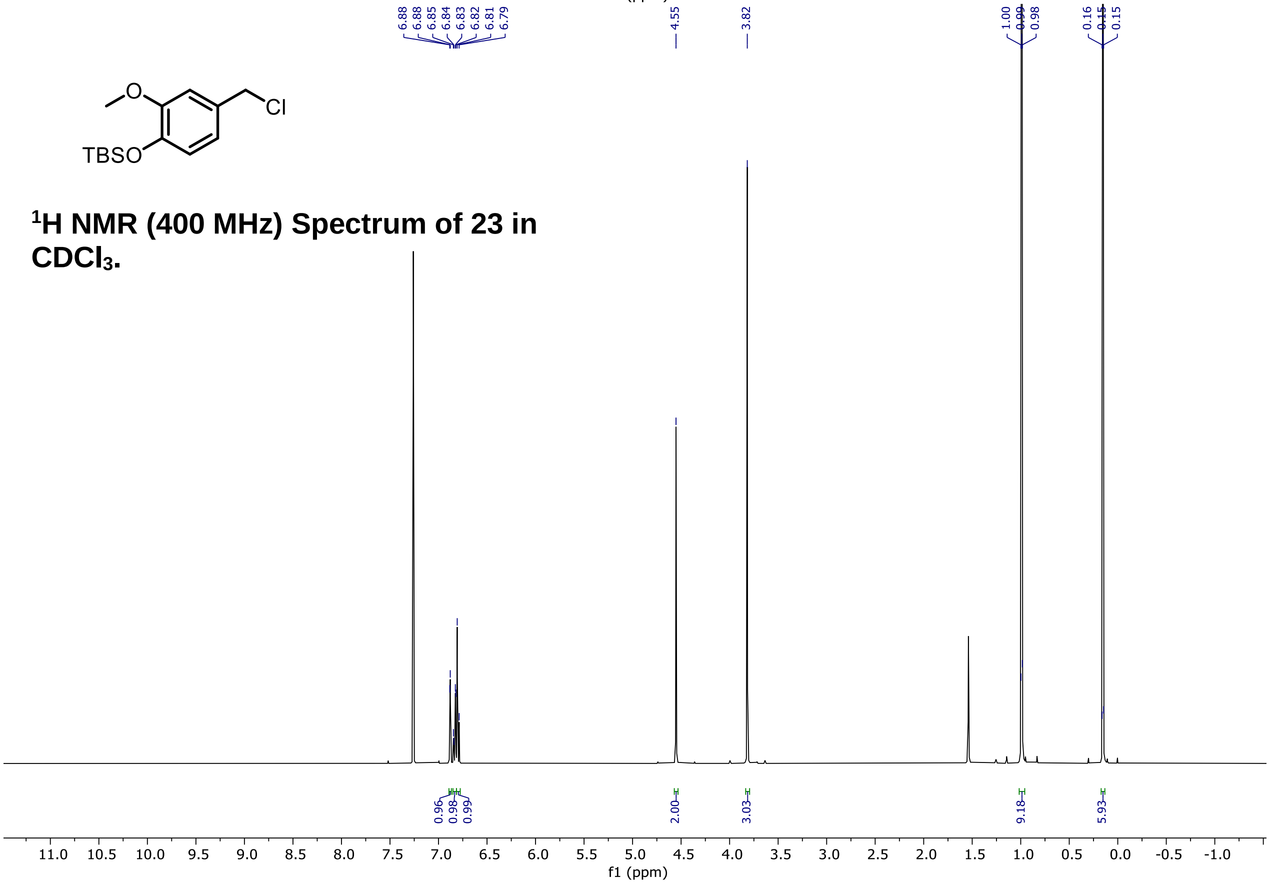

^{1}H NMR (400 MHz) Spectrum of 23 in $CDCl_3$.

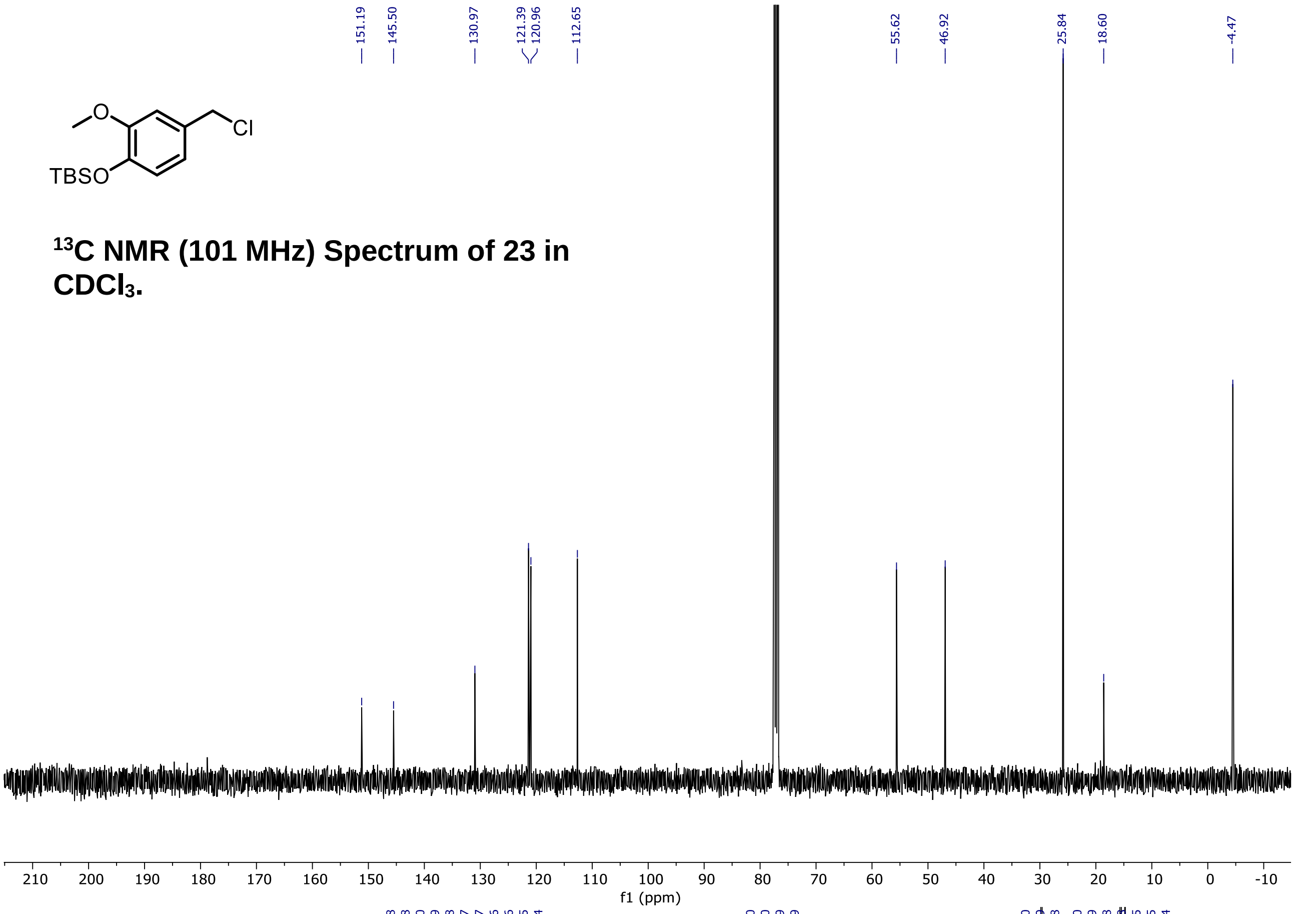
151.19
145.50
130.97
121.39
120.96
112.65
55.62
46.92
25.84
18.60
-4.47
TBSO
Cl
13C NMR (101 MHz) Spectrum of 23 in CDCl3.
f1 (ppm)

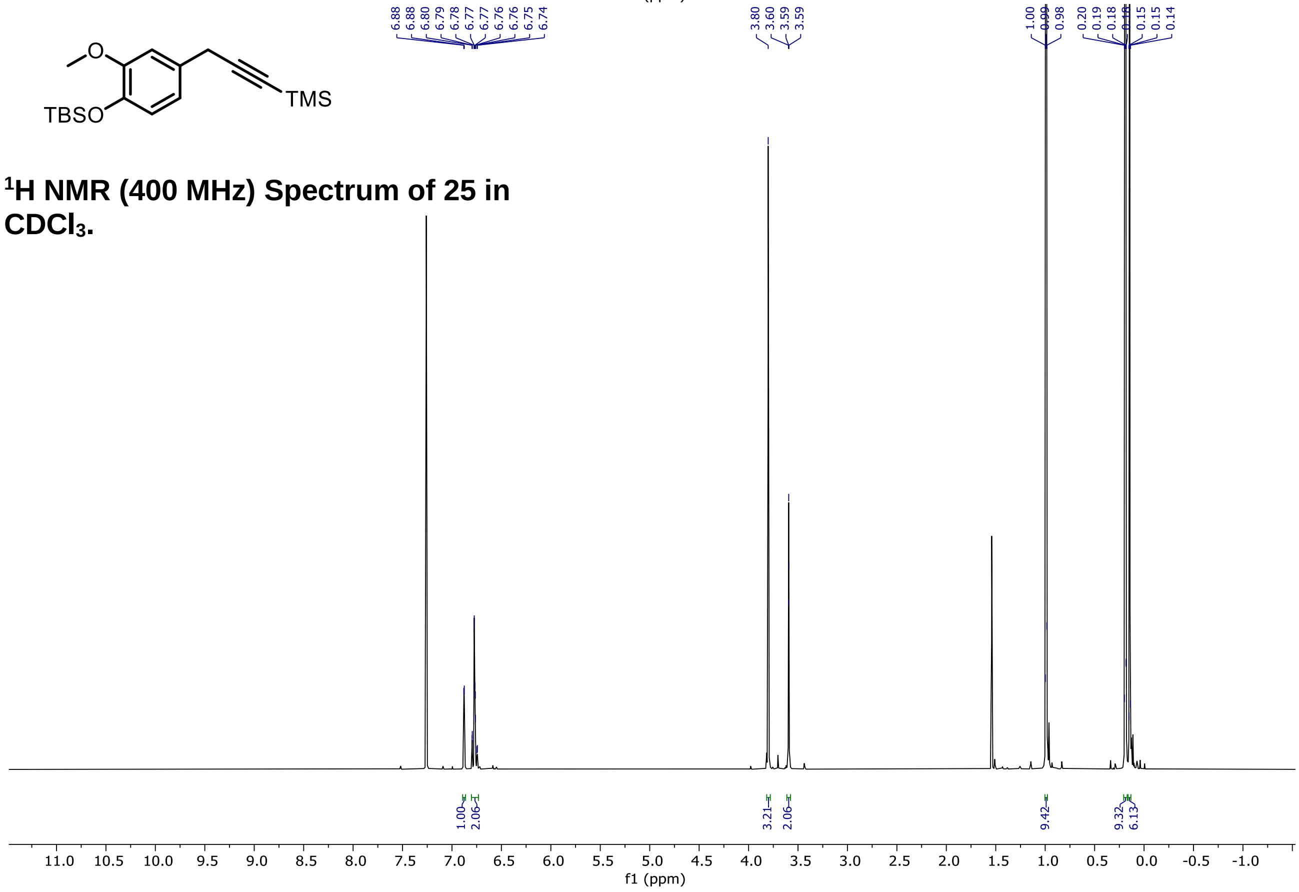
6.88
6.88
6.80
6.79
6.78
6.77
6.77
6.76
6.76
6.75
6.74
3.80
3.60
3.59
3.59
1.00
0.99
0.98
0.20
0.19
0.18
0.16
0.15
0.15
0.14
TBSO
TMS
1H NMR (400 MHz) Spectrum of 25 in CDCl3.
1.00
2.06
3.21
2.06
9.42
9.32
6.13
f1 (ppm)

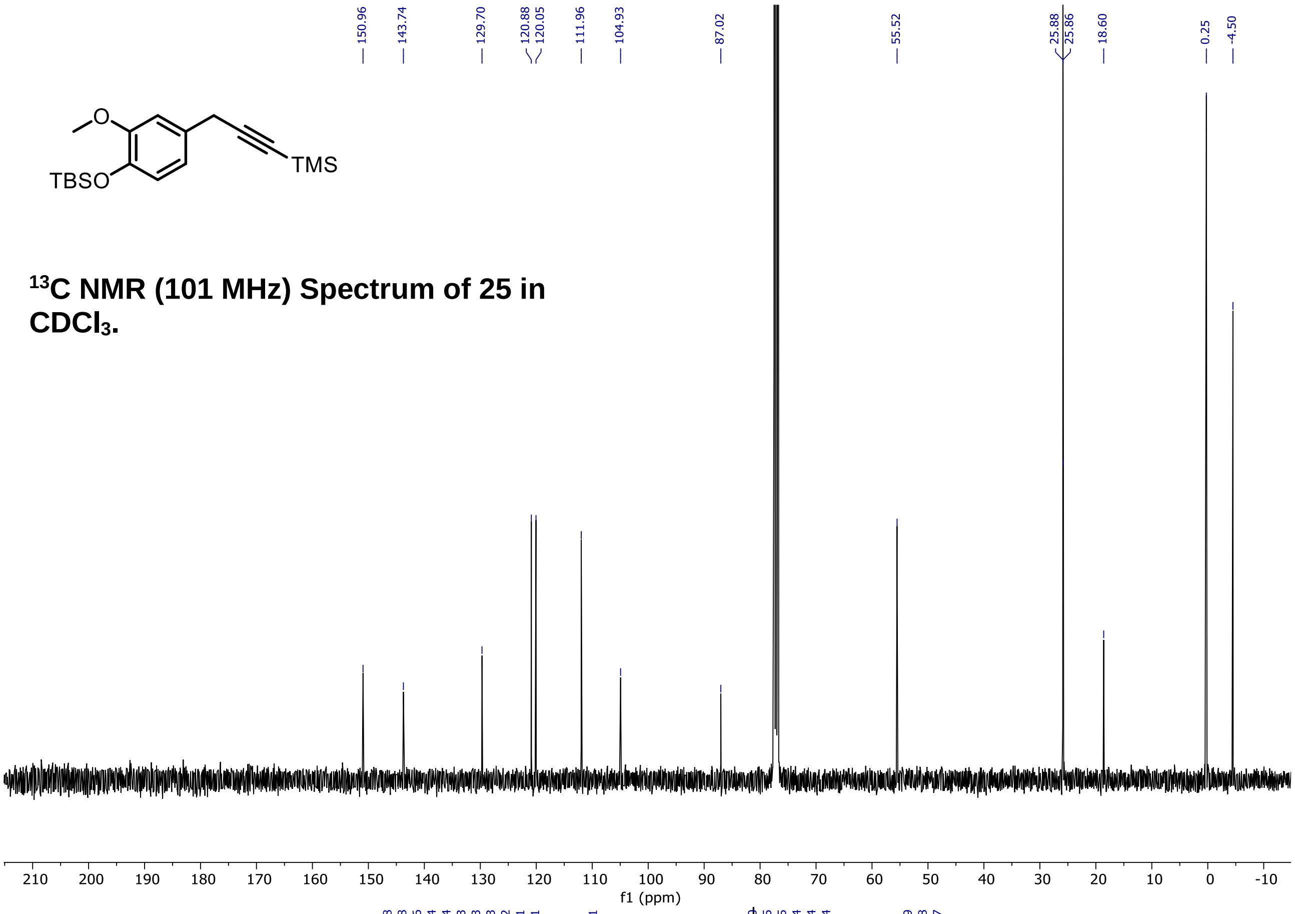

^{13}C NMR (101 MHz) Spectrum of 25 in $CDCl_3$.

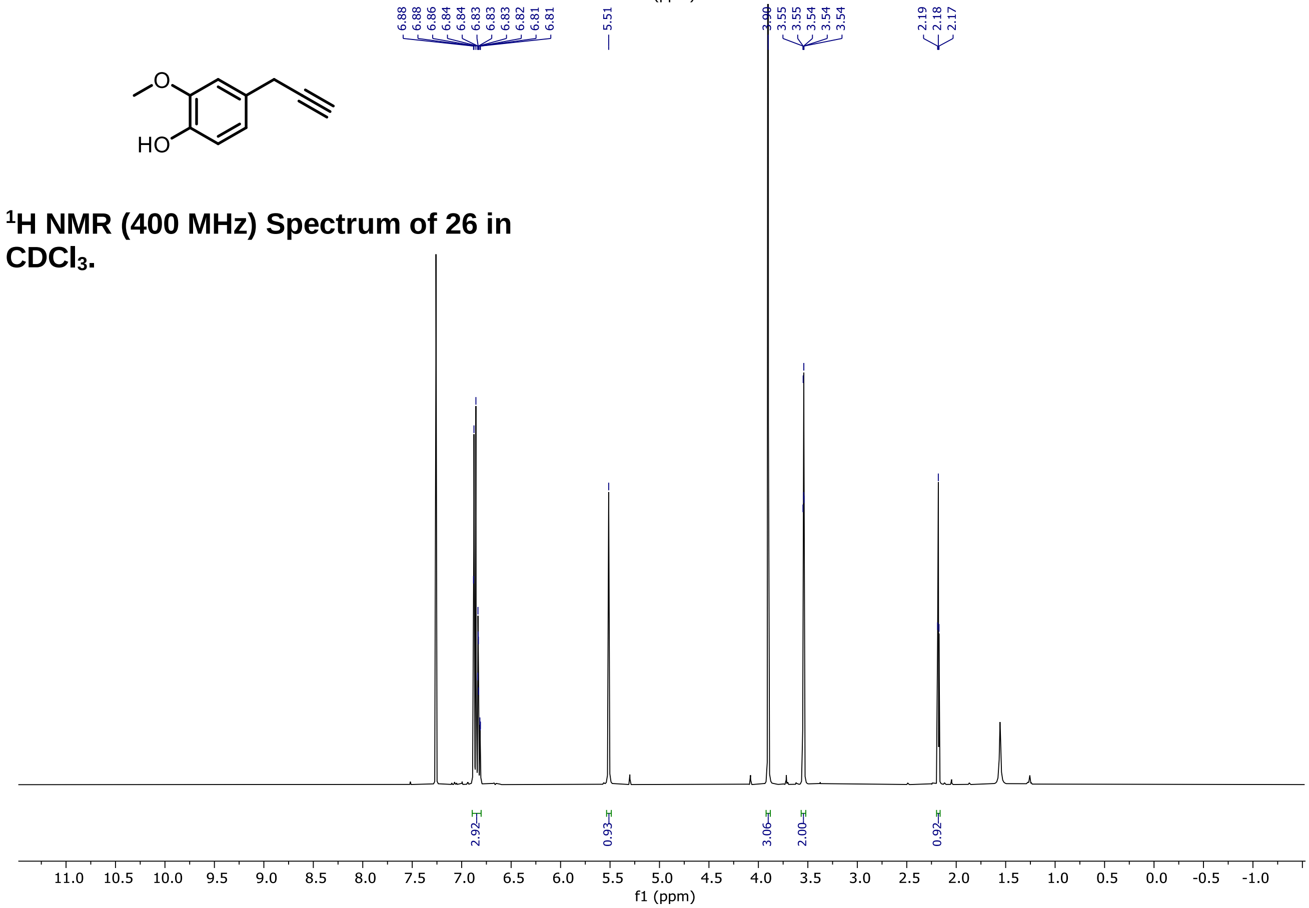

^{1}H NMR (400 MHz) Spectrum of 26 in $CDCl_3$.

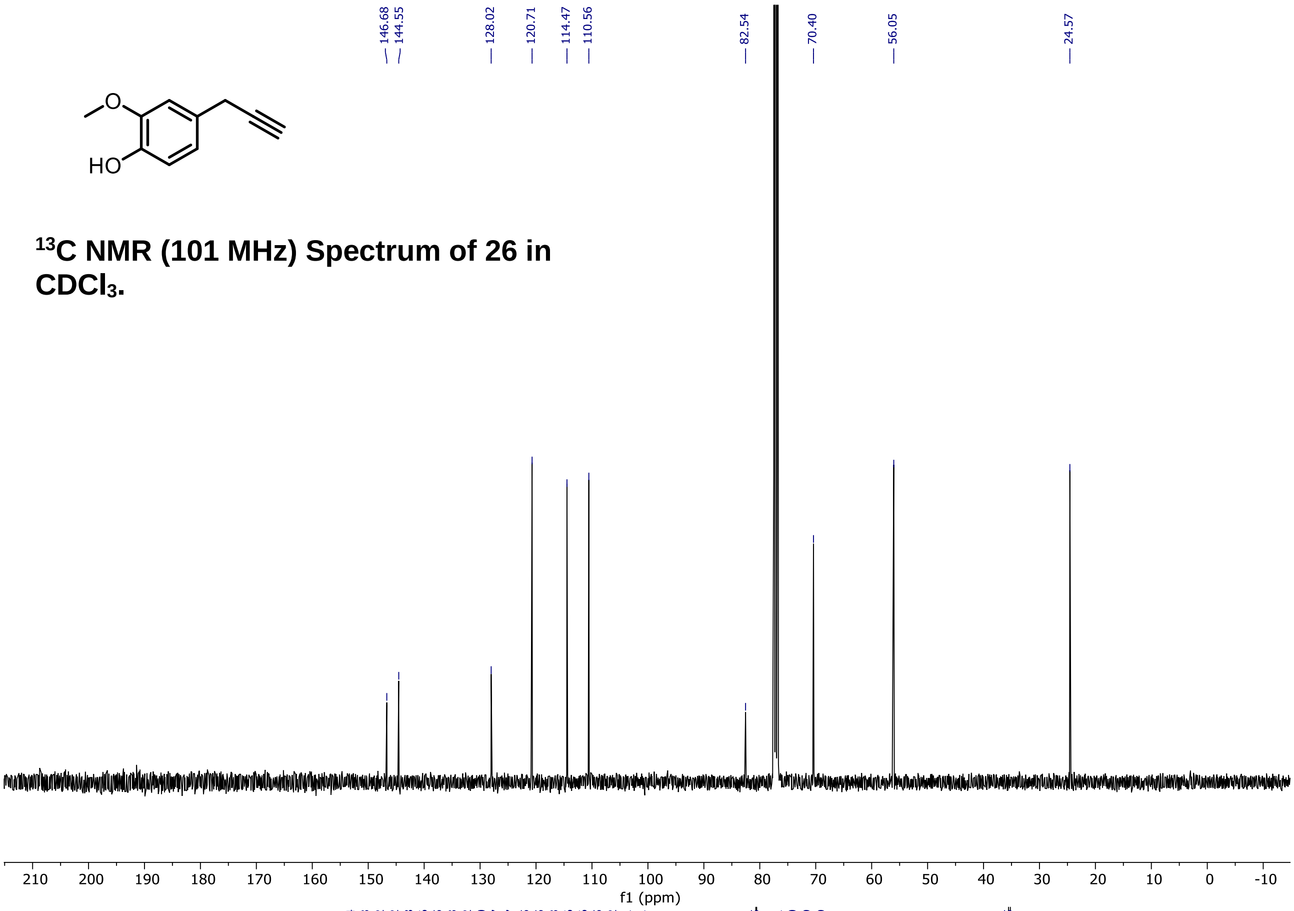

^{13}C NMR (101 MHz) Spectrum of 26 in $CDCl_3$.

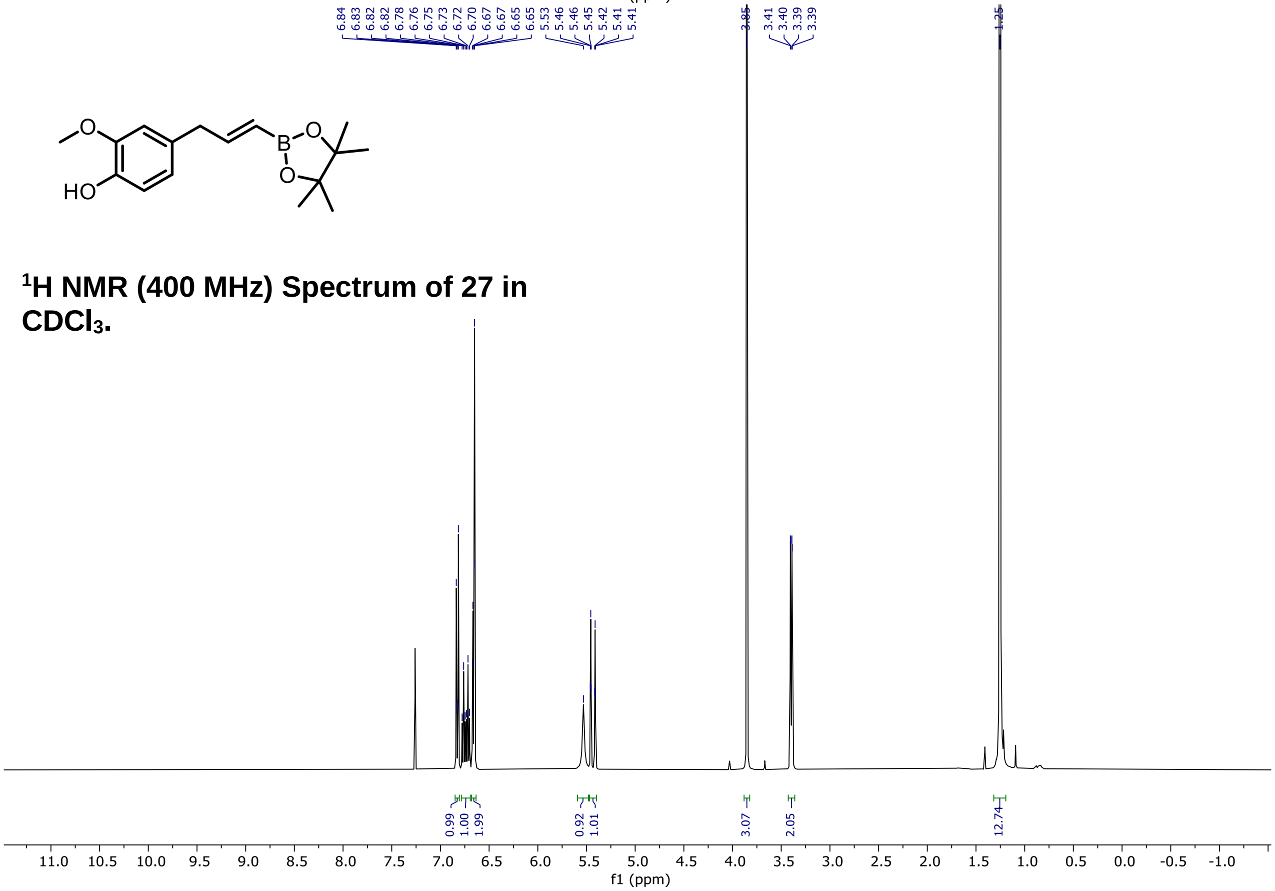

^{1}H NMR (400 MHz) Spectrum of 27 in $CDCl_3$.

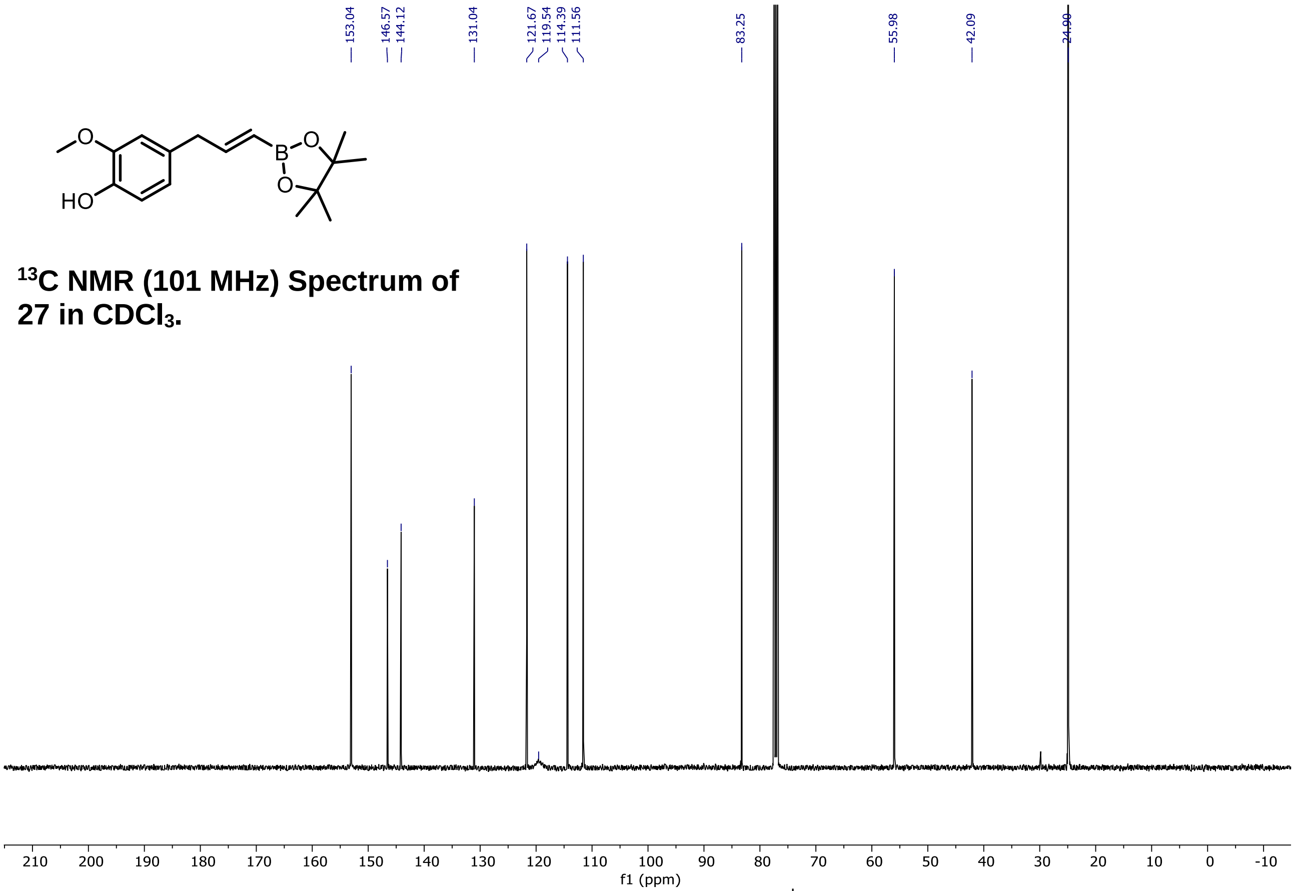
^{13}C NMR (101 MHz) Spectrum of
27 in $CDCl_3$.
f1 (ppm)

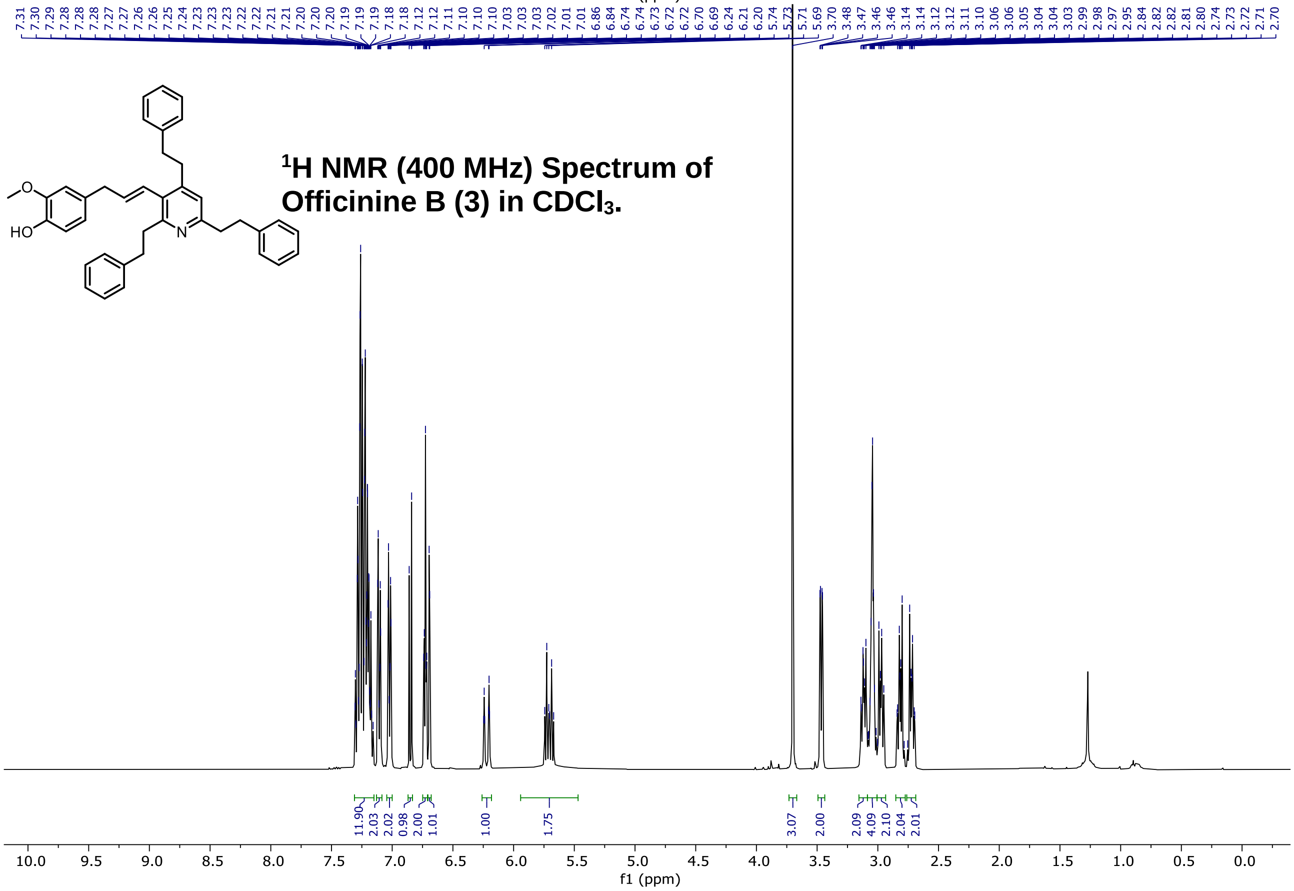
^{1}H NMR (400 MHz) Spectrum of
Officinine B (3) in $CDCl_3$.
f1 (ppm)

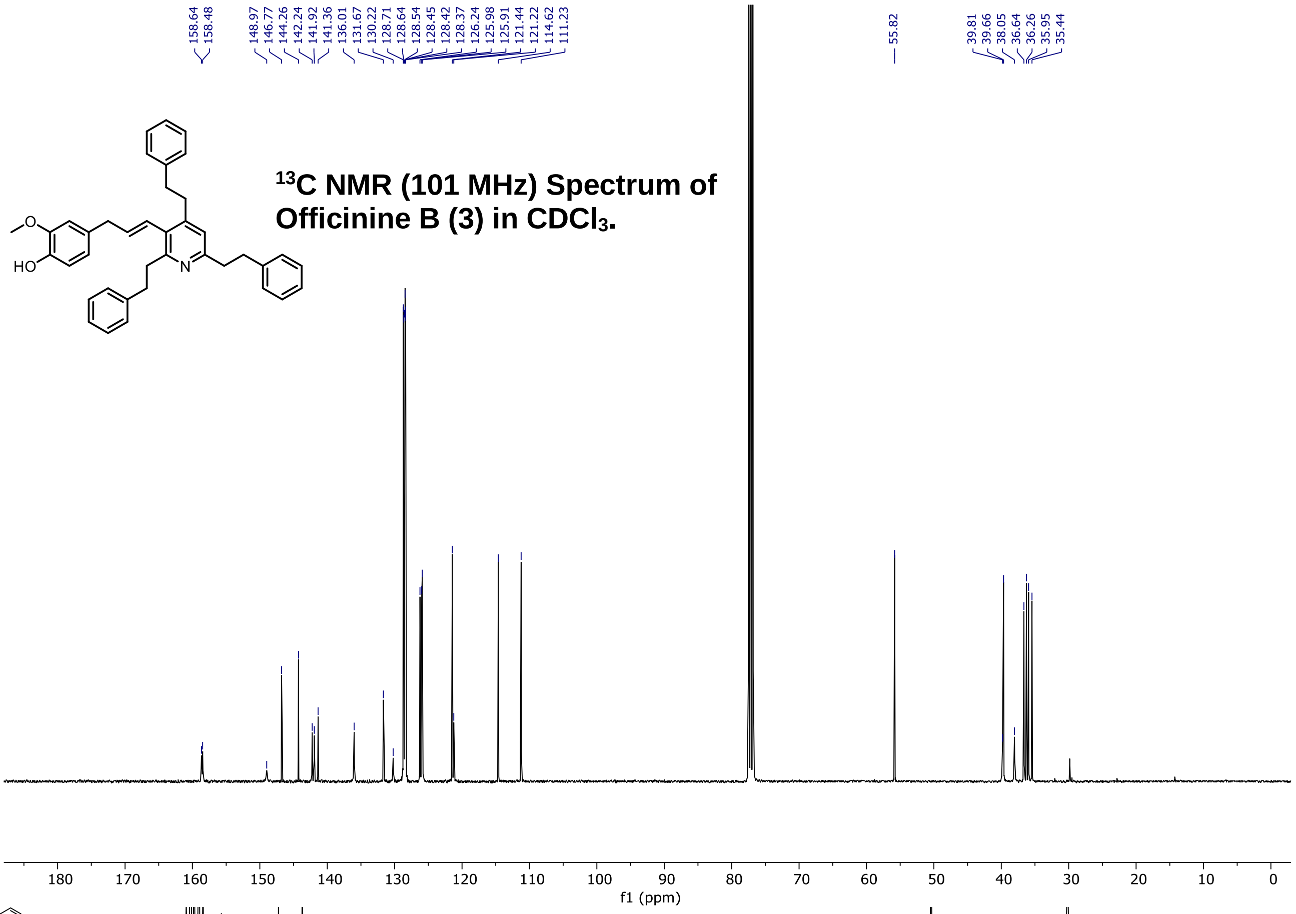

158.64
158.48
148.97
146.77
144.26
142.24
141.92
141.36
136.01
131.67
130.22
128.71
128.64
128.54
128.45
128.42
128.37
126.24
125.98
125.91
121.44
121.22
114.62
111.23
55.82
39.81
39.66
38.05
36.64
36.26
35.95
35.44
O
HO
N
13C NMR (101 MHz) Spectrum of Officinine B (3) in CDCl3.
f1 (ppm)

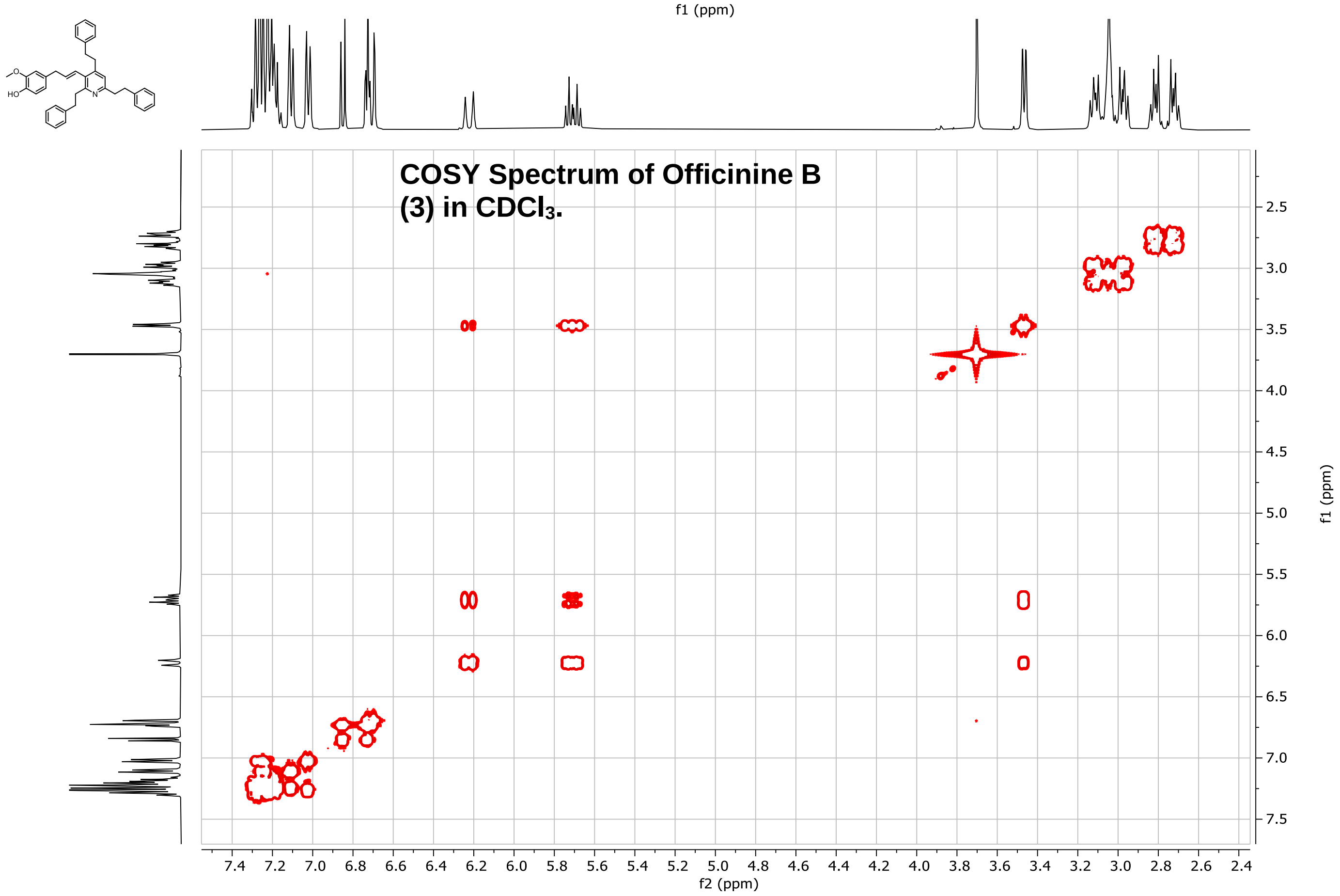

O
HO
N
COSY Spectrum of Officinine B (3) in CDCl3.
f2 (ppm)
f1 (ppm)

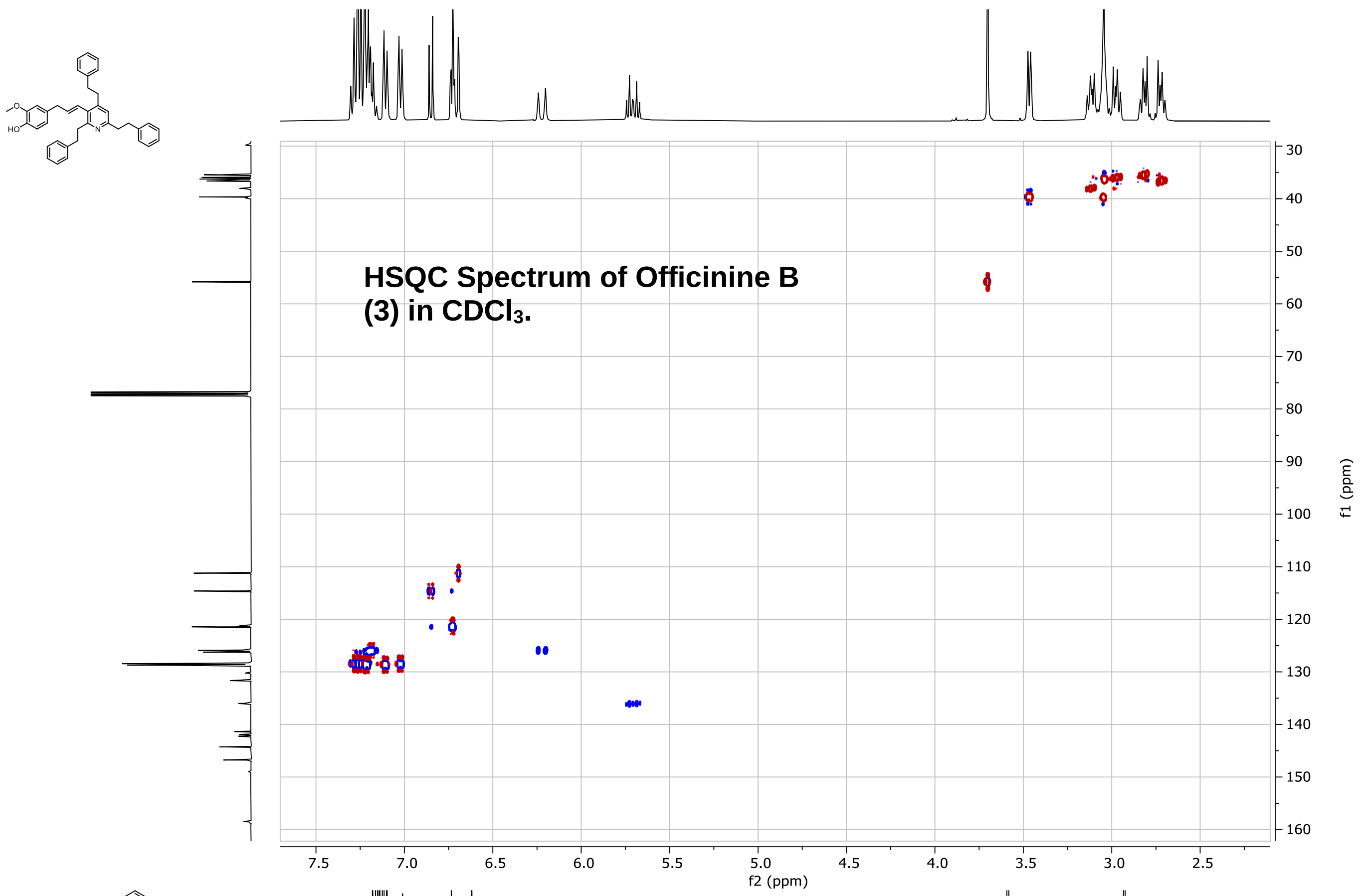

HSQC Spectrum of Officinine B
(3) in $CDCl_3$.
f2 (ppm)
f1 (ppm)

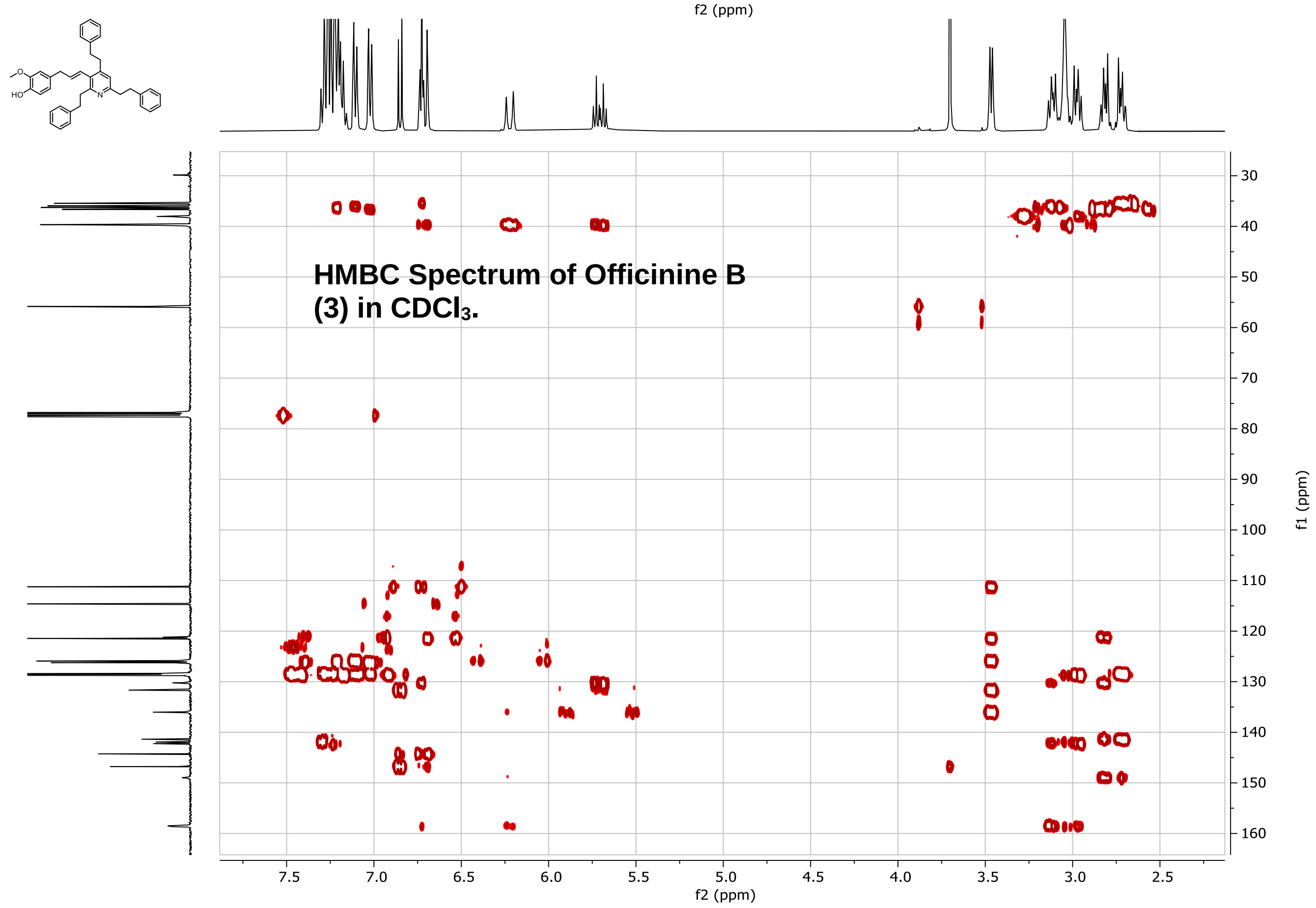

HMBC Spectrum of Officinine B
(3) in $CDCl_3$.
f2 (ppm)
f1 (ppm)

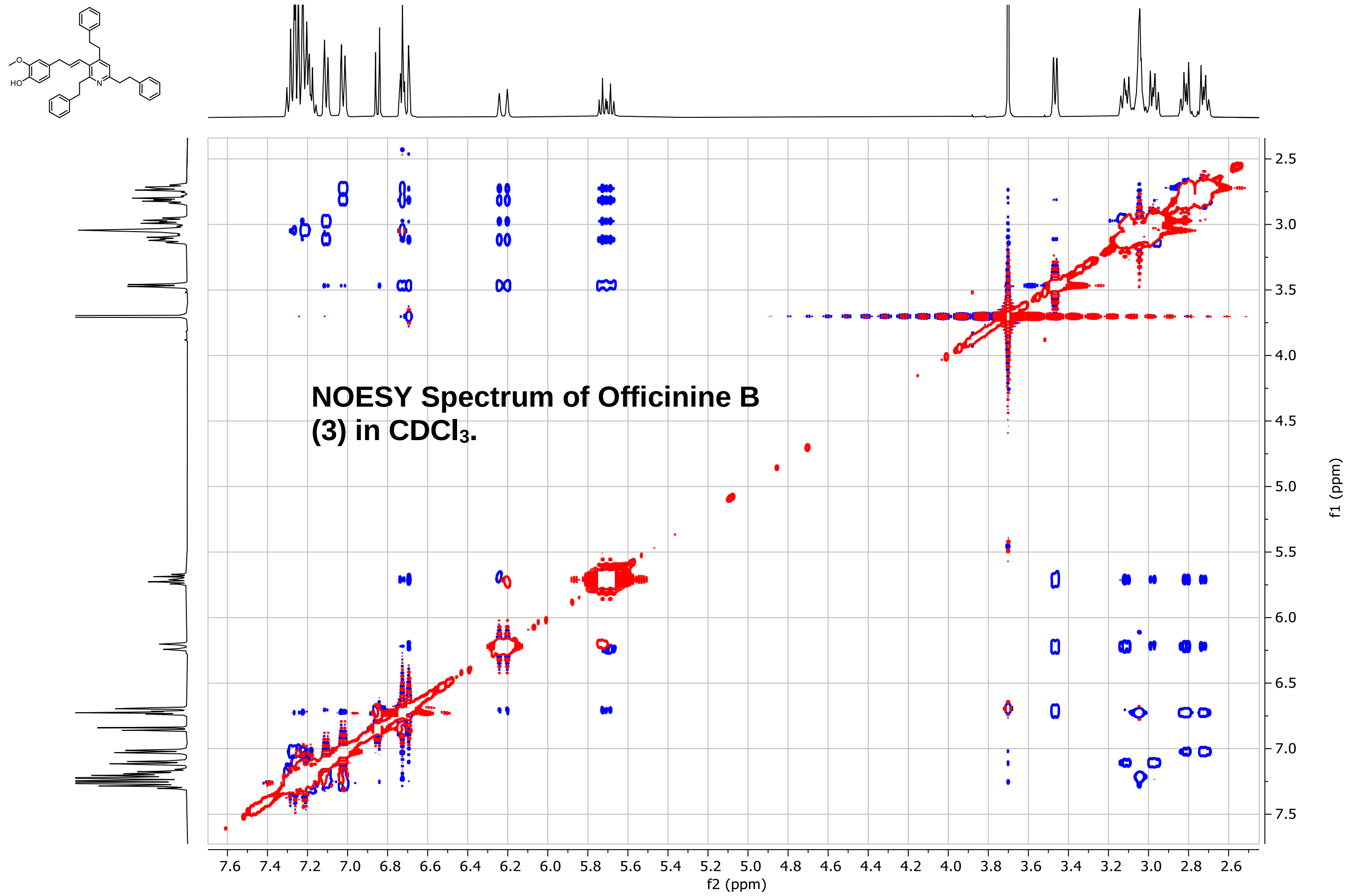

NOESY Spectrum of Officinine B (3) in $CDCl_3$.

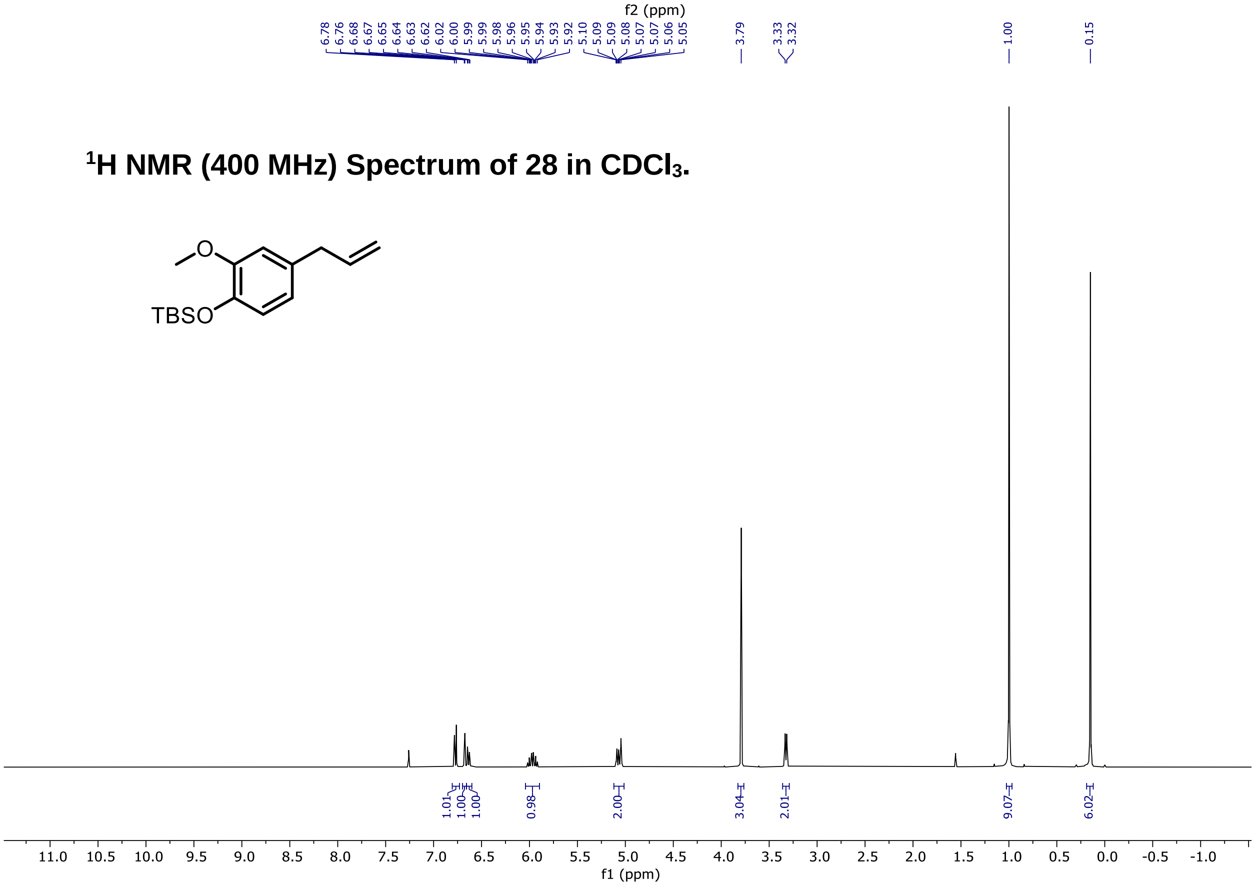

^{1}H NMR (400 MHz) Spectrum of 28 in $CDCl_3$.

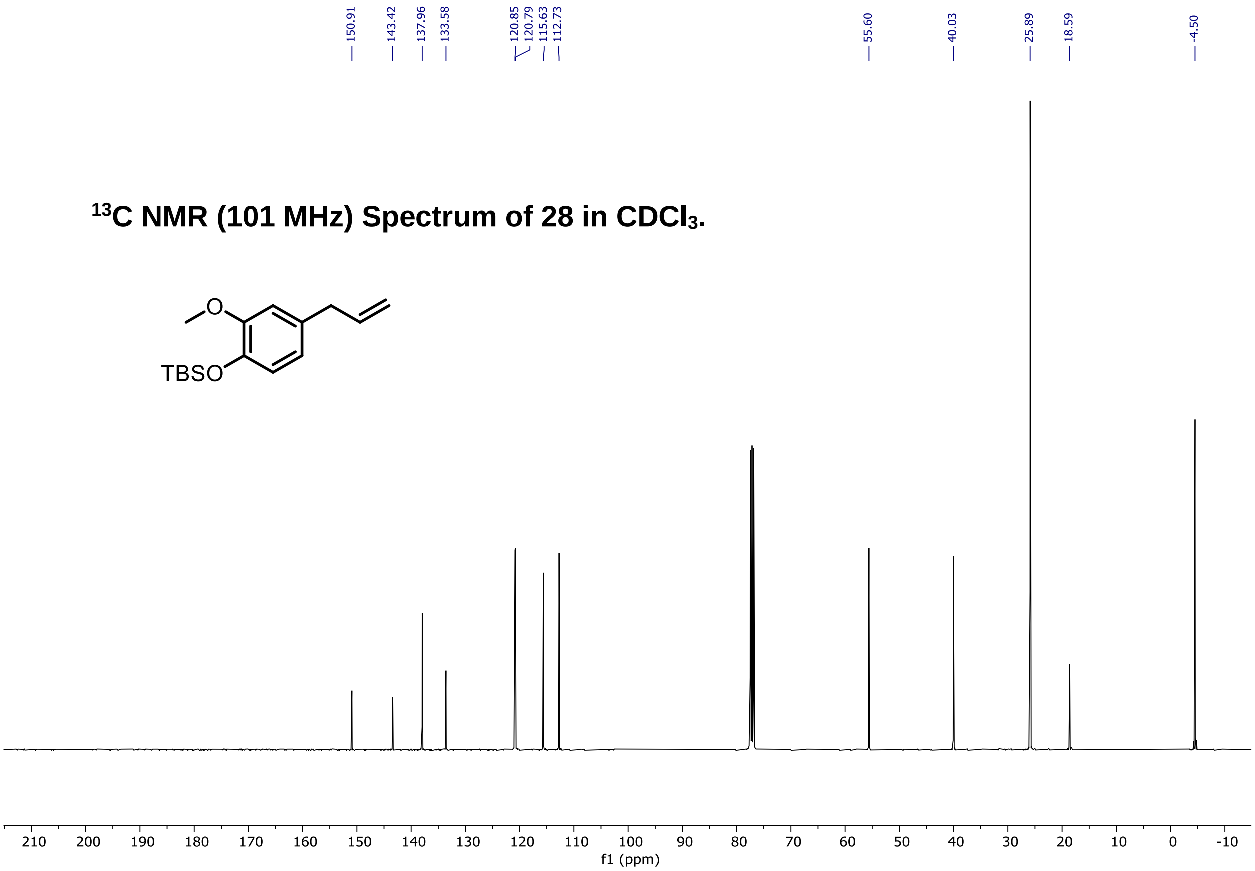
150.91
143.42
137.96
133.58
120.85
120.79
115.63
112.73
55.60
40.03
25.89
18.59
-4.50
13C NMR (101 MHz) Spectrum of 28 in CDCl3.
TBSO
f1 (ppm)

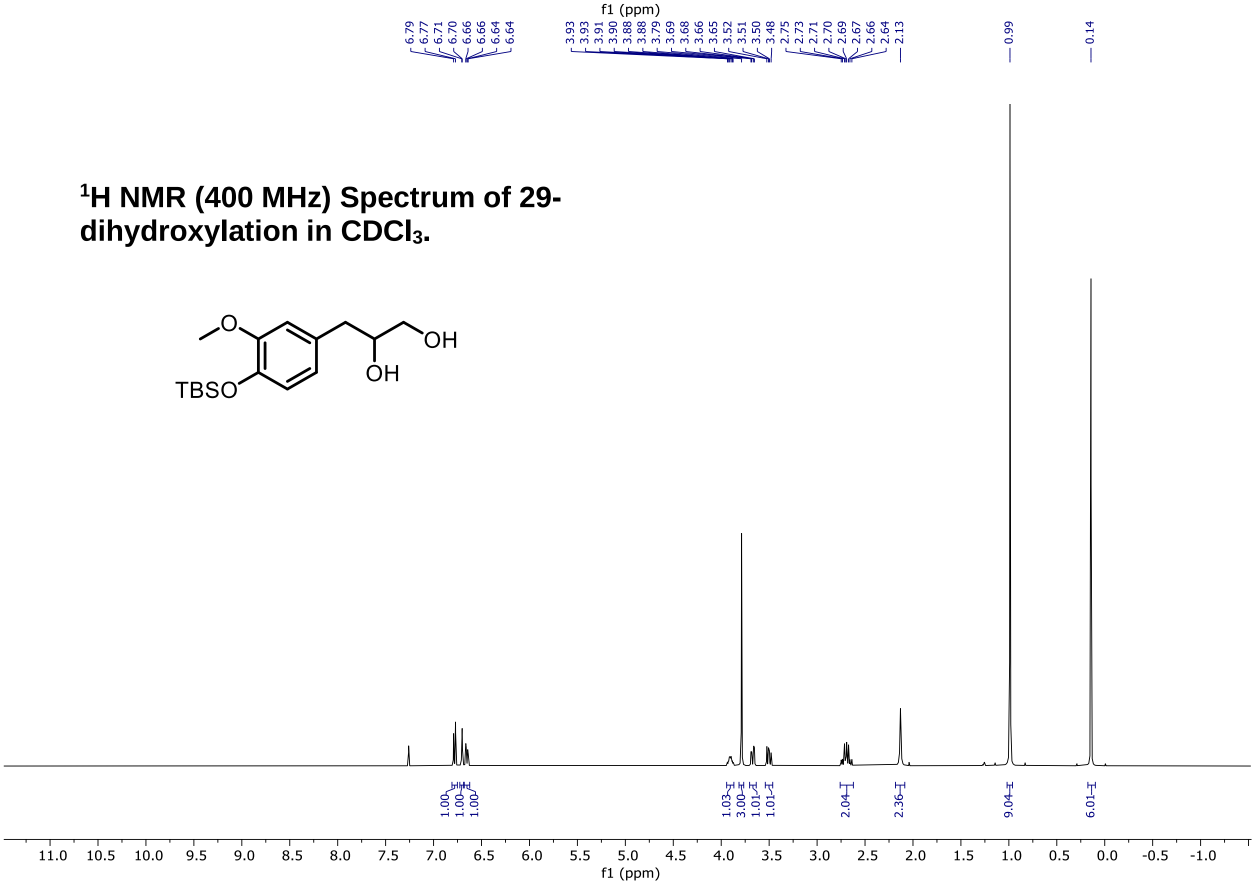
1H NMR (400 MHz) Spectrum of 29-dihydroxylation in CDCl3.
TBSO
OH
OH
0.99
0.14
2.13
f1 (ppm)

^{13}C NMR (101 MHz) Spectrum of 29-dihydroxylation in $CDCl_3$.

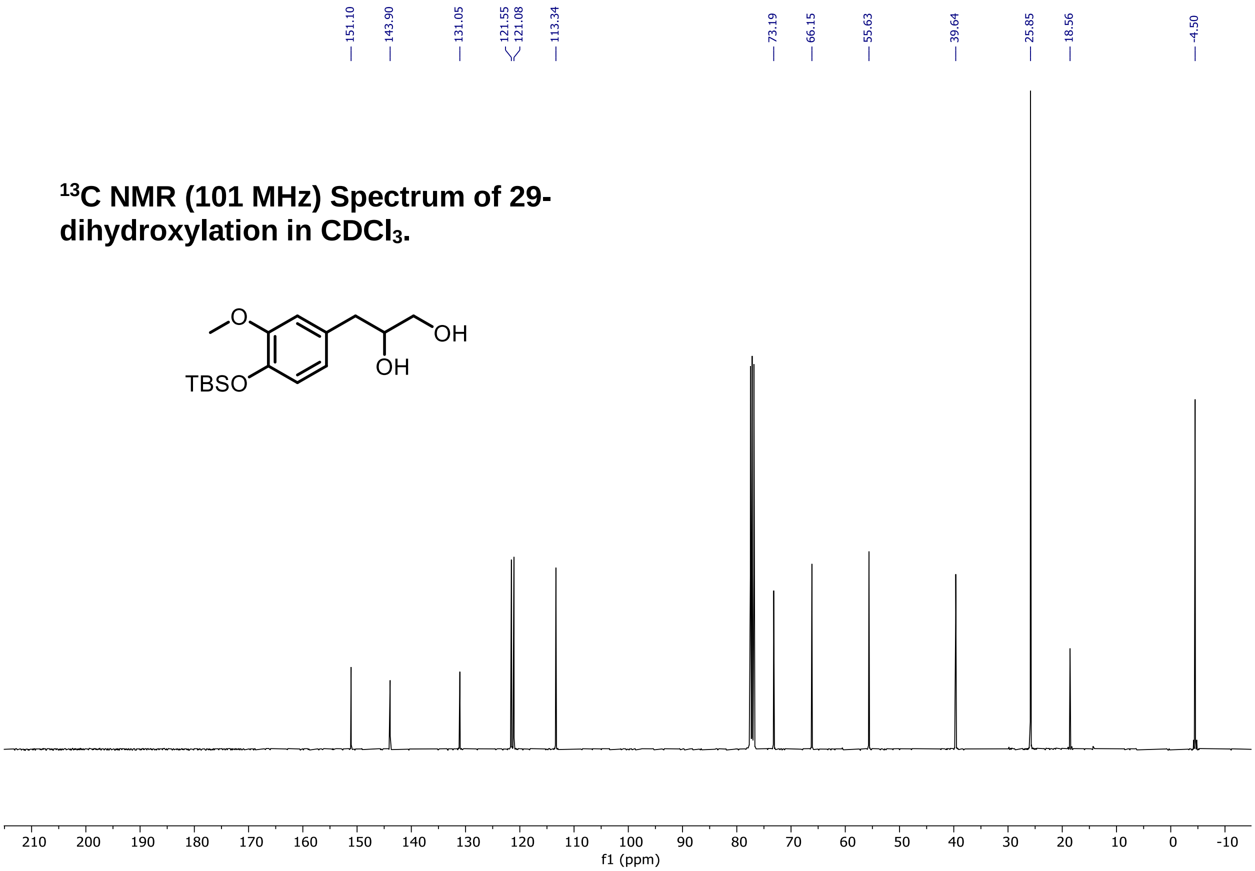

^{1}H NMR (400 MHz) Spectrum of 30 in $CDCl_3$.

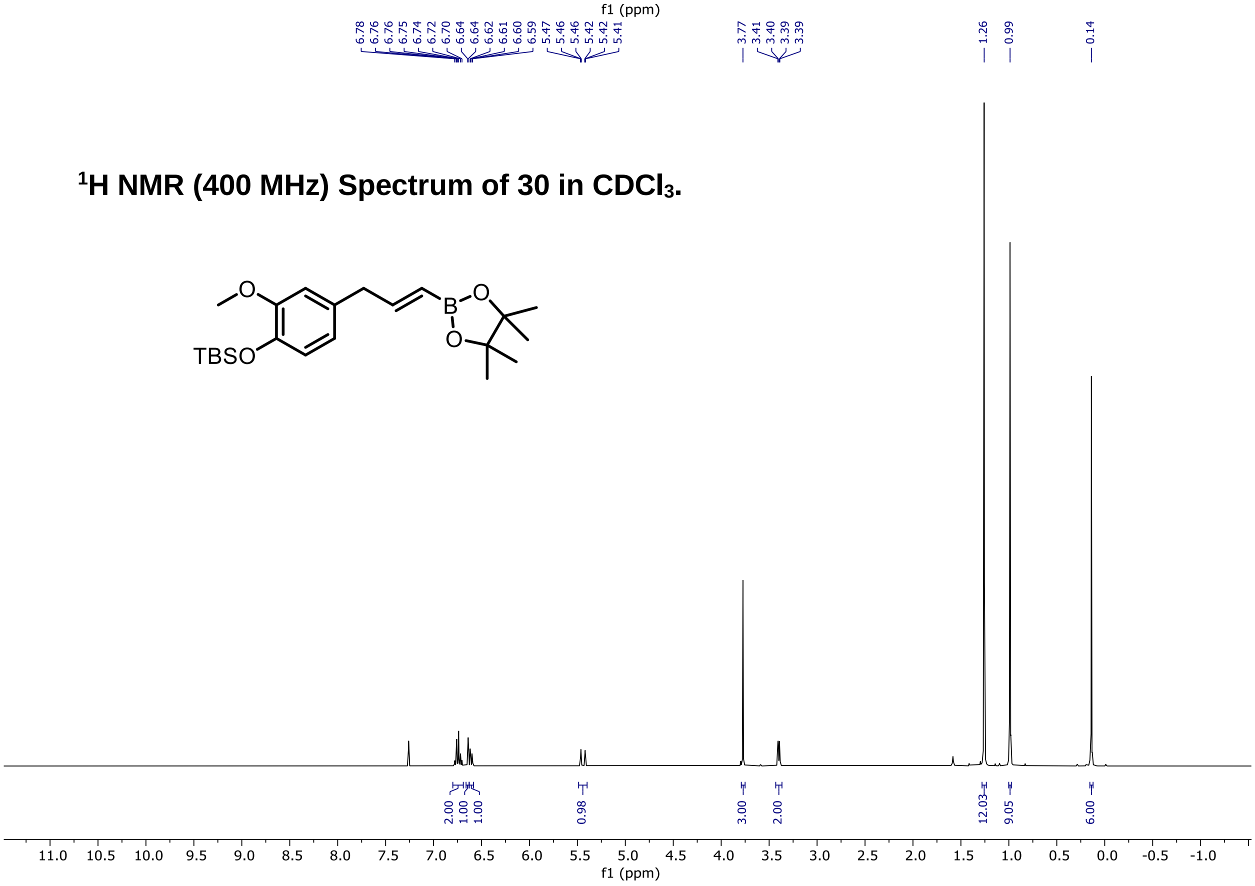

^{13}C NMR (101 MHz) Spectrum of 30 in $CDCl_3$.

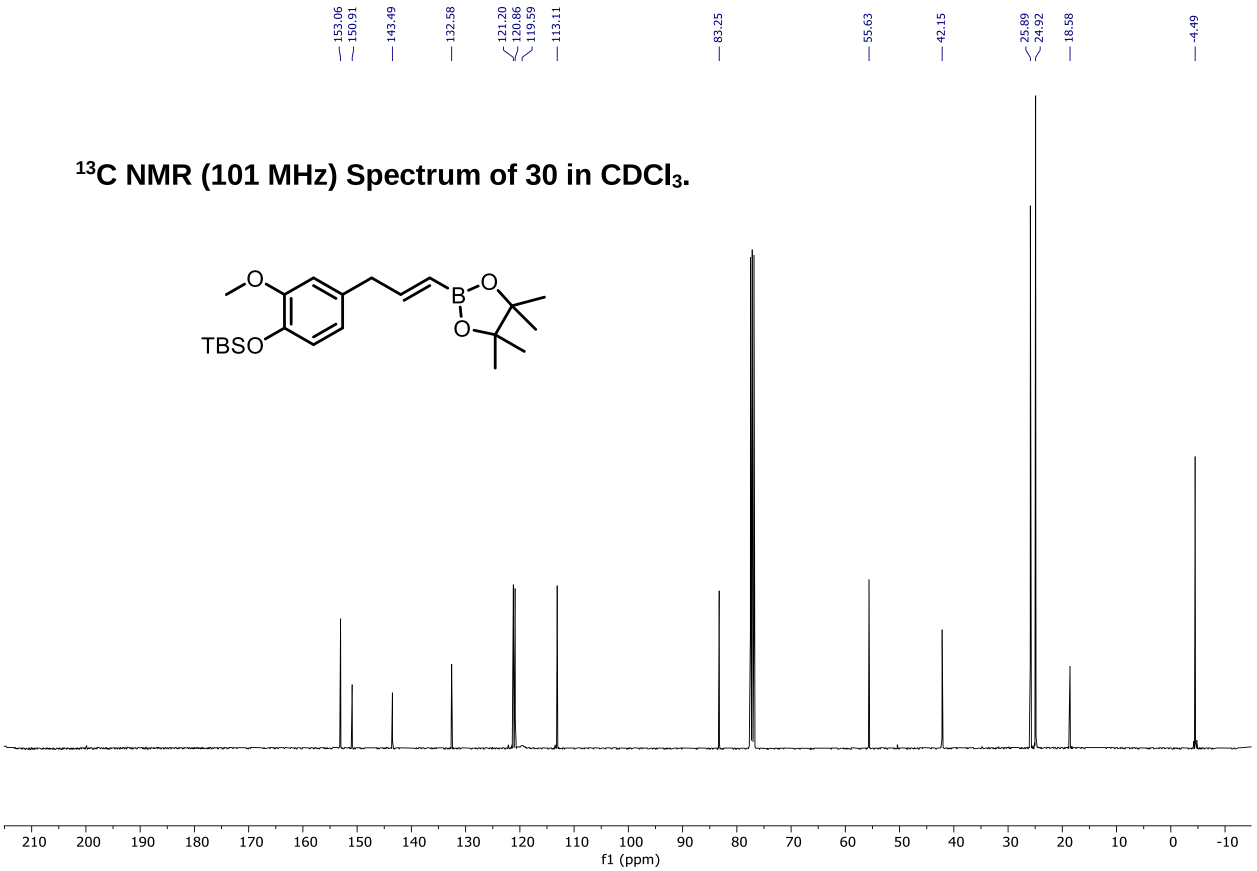

^{1}H NMR (400 MHz) Spectrum of 31 in $CDCl_3$.

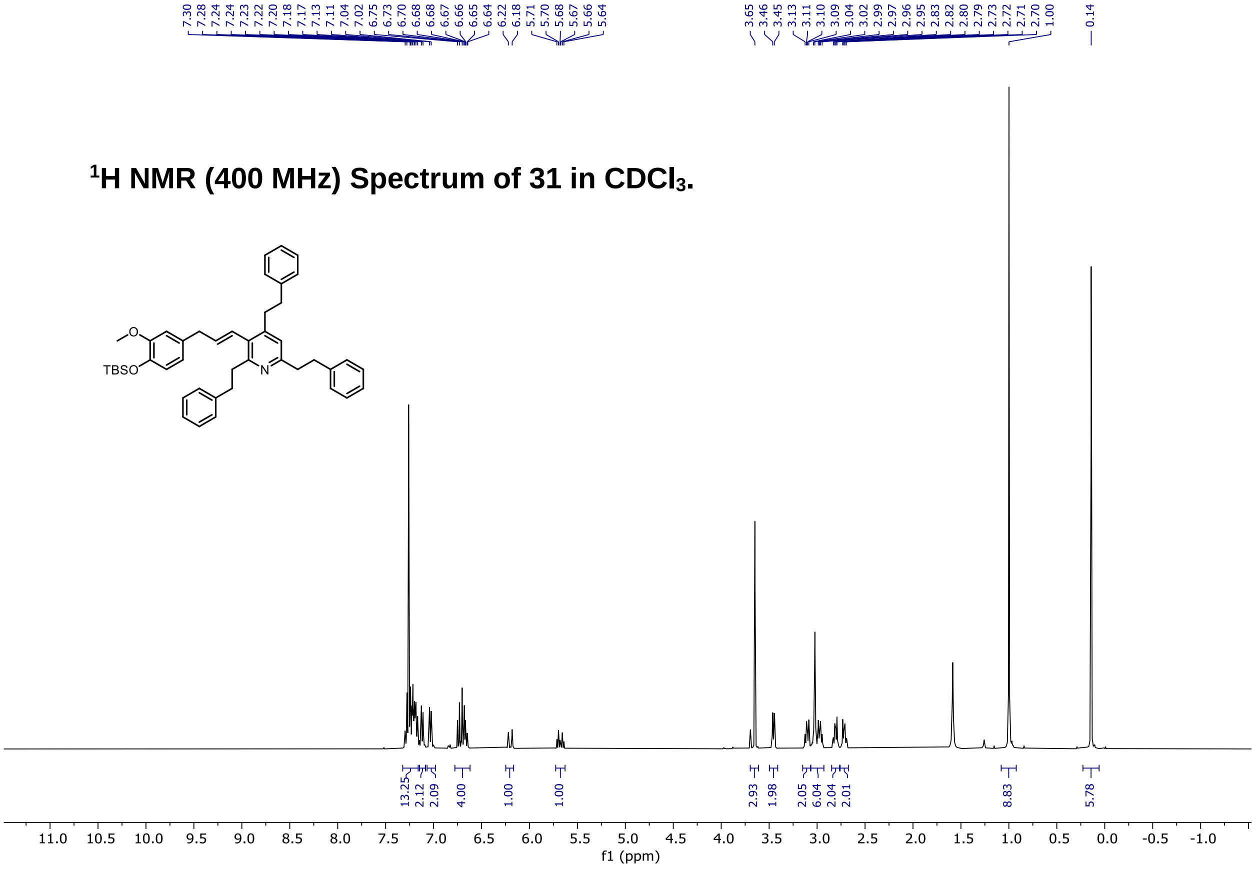

^{13}C NMR (101 MHz) Spectrum of 31 in $CDCl_3$.

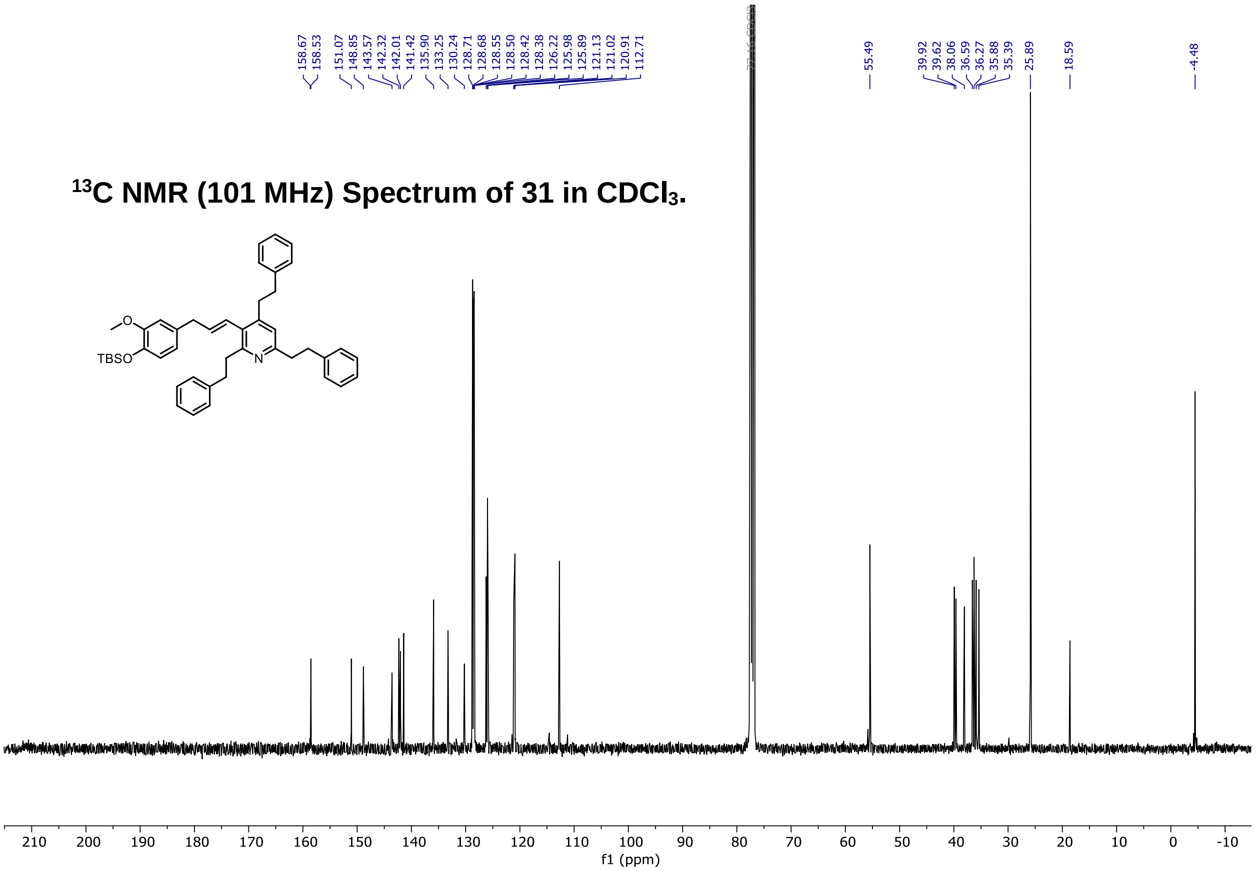

Supplement: Supplementary file 1 — ol4c03227_si_001.pdf [file ol4c03227_si_001.pdf]
